# Assessing and managing wounds of Buruli ulcer patients at the primary and secondary health care levels in Ghana

**DOI:** 10.1371/journal.pntd.0005331

**Published:** 2017-02-28

**Authors:** Naa Okaikor Addison, Stefanie Pfau, Eric Koka, Samuel Yaw Aboagye, Grace Kpeli, Gerd Pluschke, Dorothy Yeboah-Manu, Thomas Junghanss

**Affiliations:** 1 Noguchi Memorial Institute for Medical Research, University of Ghana, Legon, Ghana; 2 Section Clinical Tropical Medicine, Heidelberg University Hospital, Heidelberg, Germany; 3 Department of Microbiology, Korle—Bu Teaching Hospital, Accra, Ghana; 4 Department of Sociology and Anthropology, University of Cape–Coast, Cape-Coast, Ghana; 5 Division of Molecular Immunology, Swiss Tropical and Public Health Institute, Basel, Switzerland; 6 University of Basel, Basel, Switzerland; Kwame Nkrumah University of Science and Technology, GHANA

## Abstract

**Background:**

Beyond *Mycobacterium ulcerans*—specific therapy, sound general wound management is required for successful management of Buruli ulcer (BU) patients which places them among the large and diverse group of patients in poor countries with a broken skin barrier.

**Methods:**

Clinically BU suspicious patients were enrolled between October 2013 and August 2015 at a primary health care (PHC) center and a municipal hospital, secondary health care (SHC) center in Ghana. All patients were IS*2404* PCR tested and divided into IS*2404* PCR positive and negative groups. The course of wound healing was prospectively investigated including predictors of wound closure and assessment of infrastructure, supply and health staff performance.

**Results:**

53 IS*2404* PCR positive patients—31 at the PHC center and 22 at the SHC center were enrolled—and additionally, 80 clinically BU suspicious, IS*2404* PCR negative patients at the PHC center. The majority of the skin ulcers at the PHC center closed, without the need for surgical intervention (86.7%) compared to 40% at the SHC center, where the majority required split-skin grafting (75%) or excision (12.5%). Only 9% of wounds at the PHC center, but 50% at the SHC center were complicated by bacterial infection. The majority of patients, 54.8% at the PHC center and 68.4% at the SHC center, experienced wound pain, mostly severe and associated with wound dressing. Failure of ulcers to heal was reliably predicted by wound area reduction between week 2 and 4 after initiation of treatment in 75% at the PHC center, and 90% at the SHC center. Obvious reasons for arrested wound healing or deterioration of wound were missed additional severe pathology; at the PHC center (chronic osteomyelitis, chronic lymphedema, squamous cell carcinoma) and at the SHC center (malignant ulceration, chronic lymphedema) in addition to hygiene and wound care deficiencies.

When clinically suspicious, but IS*2404* PCR negative patients were recaptured in the community, 76/77 (98.7%) of analyzed wounds were either completely closed (85.7%) or almost closed (13%). Five percent were found to have important missed severe pathology (chronic osteomyelitis, ossified fibroma and suspected malignancy).

**Conclusion:**

The wounds of most BU patients attending the primary health care level can be adequately managed. Additionally, the patients are closer to their families and means of livelihood. Non-healing wounds can be predicted by wound area reduction between 2 to 4 weeks after initiation of treatment. Patients with clinically BU suspicious, but PCR negative ulcers need to be followed up to capture missed diagnoses.

## Author summary

Buruli ulcer (BU), currently endemic in more than 30 countries, particularly in West and Central Africa, causes chronic necrotising disease of the skin and subcutaneous soft tissue. The task is twofold, specific therapy directed against the causative agent *Mycobacterium ulcerans* and general wound management to get the often very large skin defects closed. Irrespective of wound etiology, healing requires favorable systemic conditions such as balanced nutrition, protection from trauma and infection, moist wound environment, and pain control. The authors compared wound healing in BU patients and the infrastructure and wound care practices at two levels of the healthcare system in Ghana. Our results indicate that with the basic infrastructure at the primary health care level, equipment and supplies at appropriate quality standards, well-trained health staff and adherence to established Buruli ulcer treatment and wound management guidelines, most wounds can be adequately managed. We further determined the outcome of clinically BU suspicious but not laboratory confirmed wounds and found that careful follow-up is needed to not miss important diagnoses requiring specific therapy. Patient centered care needs a horizontal approach to wound management.

## Introduction

Buruli ulcer (BU) is a chronic necrotising disease of the skin and subcutaneous soft tissue. It is currently endemic in more than 30 countries, particularly in West and Central Africa, where it is predominantly found in children [1]. The causative organism, *Mycobacterium ulcerans*, produces a macrolide toxin, mycolactone, which causes tissue destruction and inhibits local immune responses [2]. The disease commonly starts as a papule, nodule or a plaque and progresses to ulceration and often permanent disability in advanced disease [3, 4]. Current World Health Organization (WHO) guidelines for BU recommend antimycobacterial drug treatment with rifampicin in combination with streptomycin (“standard antibiotic treatment”) and refer to”growing evidence of the efficacy of some rifampicin-based oral therapies”(e.g. clarithromycin) for 8 weeks [4]. Thermotherapy shows promise, as recently demonstrated in a proof-of-principle study and a clinical trial [5, 6].

Specific antimycobacterial therapy and general wound management are equally challenging tasks to cure BU patients. Irrespective of wound etiology, healing requires favorable systemic conditions such as balanced nutrition, protection from trauma and infection, moist wound environment, and peri-lesional edema and pain control [7, 8].

Of concern in the treatment of BU wounds is that the time point of transition from mycobacterial cure to the phase where only general wound management is needed, can currently not be determined with certainty. Persistent *M*. *ulcerans* infection, relapse, immune reconstitution-associated so called paradoxical reactions and secondary infections by other pathogens are all possible differential diagnoses of failing wound healing [9–11]. Mycolactone-based point-of care tests may solve this problem in the future. Awareness for this problem comes from clinical monitoring of wounds in the context of clinical trials [6, 12–14].

We are not aware of large prospective wound management studies conducted outside clinical BU trials capturing real life condition in the health care system. In most health care centers of countries with limited resources, wound management guidelines are not strictly implemented. Additionally, vertical programs select disease-specifically leaving wounds which are not in the focus unattended.

In the present study, patients with IS*2404* PCR positive and negative ulcers were prospectively observed, comparing wound management at a center of the primary and the secondary health care levels. Additionally, patients with ulcers clinically diagnosed as BU at the PHC center but not confirmed by IS*2404* PCR were recaptured in the community to verify the correctness of the classification as non-BU cases, to assess the course of wound healing, to make a final diagnosis and to provide treatment, if wounds persisted.

## Materials and methods

The study was conducted at the Obom Health Center, a primary health care (PHC) community-based, outpatient facility in the Ga South Municipality and the Ga West Municipal Hospital, Amasaman, which is the main referral hospital at the secondary health care (SHC) level providing in- and outpatient health services in the Ga West Municipality of the Greater Accra Region. Both institutions are the main health care facilities that offer BU case management services in the respective districts. The two districts, which were previously joined as the Ga-District, report the second highest number of BU cases in Ghana and receive the worst cases nationwide.

A prospective observational study was conducted on patients with clinically suspicious IS*2404* PCR positive and negative ulcers enrolled and followed-up from October to December 2013 at the SHC center and October 2013 to August 2015 at the PHC center (see S1 Study protocol). Written consent of patients willing to participate, or their representatives was obtained before enrolment.

For the IS*2404* PCR positive cases, complete medical and wound histories were taken and thorough physical examination, wound assessment, photo-documentation and baseline investigations performed and entered in case report forms (CRF—see S1 Case Report Form (CRF) for enrolment (Day 0) and S2 Case Report Form (CRF) Follow-up). Ulcers were assigned to the WHO BU categories: Category I—single, small lesion less than 5 cm in diameter; Category II—single large lesion 5–15 cm in diameter; Category III—multiple lesions, extensive lesion >15 cm, lesions in the head and neck region, disseminated and mixed forms, bone and joint involvement [4].

The body mass index (BMI) of each patient was determined and patients put into different weight categories according to WHO criteria. For further details see Table 1.

**Table 1:** Weight categories according to Body Mass Index (BMI) [15].

Category	Adults BMI (kg/m^2^)	Children (less than 20 years) BMI for age
Underweight	<18.50	<5^th^ percentile
Normal weight	18.50–24.99	5^th^ to <85^th^ percentile
Overweight	≥25.00	≥85^th^ to <95^th^ percentile
Obesity	≥30.00	≥95^th^ percentile

On weekly follow-ups, the ulcers were assessed, photo-documented and the results entered in a follow-up CRF. This was done for each patient until complete wound closure or end of study period.

For IS*2404* PCR positive ulcers which failed to heal, additional investigations to determine the cause of delayed healing were carried out. Non-healing ulcers were assessed by wound management experts, a local expert at the level of the BU unit in Amasaman, a university hospital-based plastic surgery and burns expert at Korle-Bu teaching hospital who serves as consultant to the BU unit, and a plastic surgeon from Berne, Switzerland, who has longstanding experience of surgical BU treatment in West Africa. Assessment was performed on the basis of standardized case vignettes with demographic patient data, medical history, information on treatment and the course of wound healing, including photo documentation (see S3 Case Reports of the Primary Health Care Center (PHC) at Obom (OHC) and S4 Case Reports of Secondary Health Care Center (SHC) at the Hospital Amasaman (AMH)). The answers were tabulated and analyzed.

Wound infection was diagnosed based on the presence of the following local and systemic indicators: area of redness and hyperthermia around the wound, lymphangitis, localized pain, increased amounts and change in colored exudate, malodor, delayed or abnormal healing, wound breakdown, increased systemic temperature, general malaise, and increased leucocyte count.

Severity and frequency of pain was assessed at each dressing session. A scale of mild to severe pain was used (see S1 Study protocol). Additionally, patients were interviewed to describe their perception of wound pain and the use of analgesics. To distinguish uncomplicated, well healing wounds from chronic, arrested or deteriorating wounds, the progress of wound healing was analyzed using the criteria suggested by Flanagan: reduction of the wound area by 20 to 40% within 2 to 4 weeks of treatment initiation [16].

Patients in whom BU had been suspected at the PHC center on clinical grounds but the diagnosis rejected on the basis of a negative IS*2404* PCR result were recaptured in the community. Once the cases were found, the original lesions were identified, described and photo-documented. Wounds were put into three categories: healed, healing and non-healing. For the non-healing wounds, additional investigations were performed to determine the cause.

Infrastructure and wound care practices at both health facility levels were assessed on the basis of direct observation, employing the following criteria using the WHO guidelines as reference standard [7]:

1. Infrastructure assessment
  - Availability of facilities (wound dressing room, sterilization and surgical facilities) to manage the different categories of BU lesions, in particular those with and without bacterial infections, seen at the PHC and SHC centers.
  - Appropriateness of patient flow, i.e. keeping patients with bacterially contaminated ulcers separate from patients with clean ulcers after skin grafting and outpatients from inpatients to minimize spread of nosocomial infections into the community.
2. Assessment of wound care practices:
  - Appropriateness of patient information, in particular explanation of wound management procedures including dressing, pain control and surgery.
  - Appropriateness of wound cleansing, in particular whether wound cleansing was done with copious amounts of potable water as recommended, or by surface cleaning with wet cotton wool/gauze.
  - Availability of material and appropriateness of dressing technique.
  - Appropriateness of infection and pain control.

### Ethical clearance

The study was conducted in accordance with the ethical principles that have their origin in the Declaration of Helsinki and in compliance with ICH-GCP, ISO 14155–1 and -2, and the applicable laws and regulations of the participating country.

Approval was obtained from the Institutional Review Board of the Noguchi Memorial Institute for Medical Research, Legon, Ghana and the Ghana Health Service Ethical Review Committee, reference number, GHS-ERC: 07/07/13 (see S1 Ethical Clearance).

## Results

### Patient characteristics

**Fig 1:**
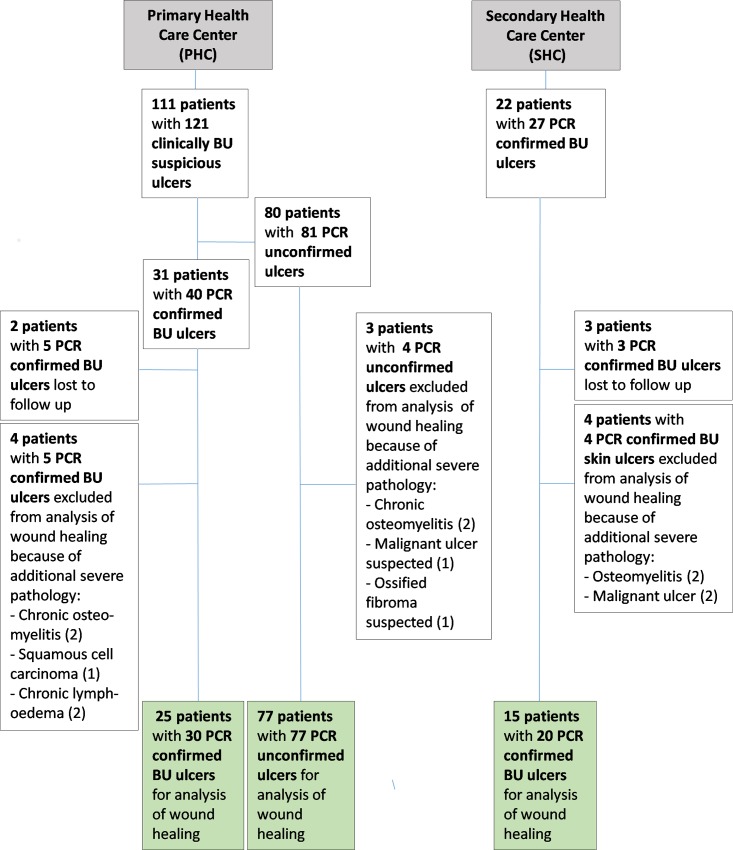
Study algorithm at the PHC center and the SHC center.

A total of 133 patients, 111 at the PHC center and 22 at the SHC center, were enrolled in the study. At the PHC center, 31 patients were IS*2404* PCR positive, and at the SHC center 22. At the PHC center 25 of the IS*2404* PCR positive and 77 of the negative patients, and at the SHC center 15 of the IS*2404* PCR positive patients entered the analysis of wound healing. For further details see Fig 1.

**Fig 2:**
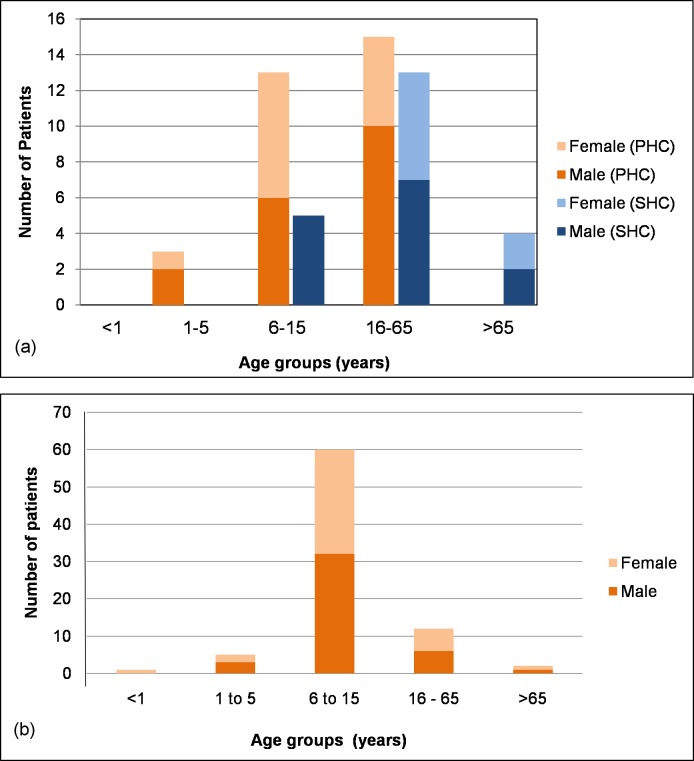
Age and sex distribution of patients with IS*2404* PCR positive ulcers at the PHC and the SHC centers (a) and patients with PCR negative ulcers at the PHC center (b).

As expected for an African BU patient population, more than half of the IS*2404* PCR positive patients at the PHC center (53%; 16/31) were younger than 16 years (Fig 2A). Dominance of children was more pronounced among the patients with PCR negative ulcers, where 82.5% (66/80) were younger than 16 years (Fig 2B). In contrast, the majority of PCR-confirmed BU patients at the SHC center (77.3%; 17/22) were older than 15 years (Fig 2A). Among the PCR-confirmed BU patients, the male to female ratio was less balanced (63.6% males at the SHC center and 58.1% at the PHC center), than among the patients with PCR negative ulcers (52.5% males).

Six percent (2/31) of patients at the PHC center and 36% (8/22) at the SHC center were underweight. The general health status of patients at the PHC center was good; comorbid conditions identified were HIV infection (n = 1), sickle cell disease (n = 1) and arterial hypertension (n = 1). At the SHC center, comorbid conditions were more abundant and included arterial hypertension (n = 7), diabetes mellitus (n = 2), sickle cell disease (n = 1), HIV infection (n = 1), asthma (n = 1), peptic ulcer disease (n = 1) and epilepsy (n = 1).

### Lesion characteristics

**Fig 3:**
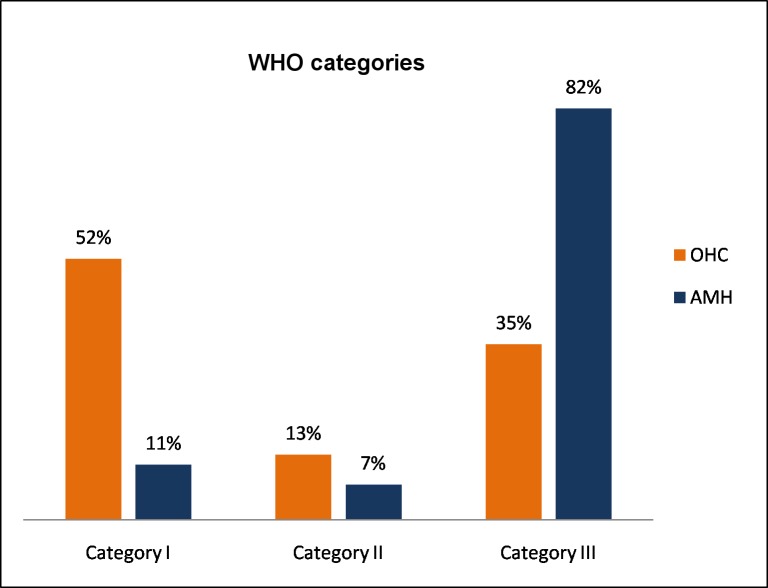
WHO categories of all BU patients with IS*2404* PCR positive ulcers enrolled at the PHC and the SHC centers

All IS*2404* PCR positive BU patients presented with ulcerative lesions (Fig 3). At the PHC center 30 ulcers were assessed and all were located on the lower (93.3%) and upper (6.7%) extremities with 63.3% of ulcers on the right side. Among the 20 ulcers assessed at the SHC center, 87.5%, 4.2% and 8.3% were located on the lower extremities, upper extremities (63.6% on the right side) and the face, respectively.

### Wound healing

**Fig 4:**
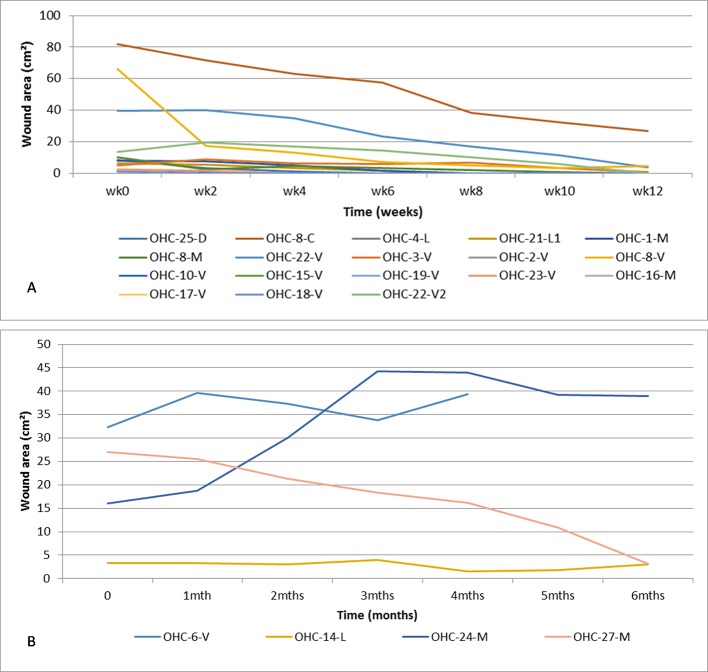
Development of IS*2404* PCR positive ulcers measured by change in wound area over time at the PHC center (the patient code “OHC” refers to the patient numbering at the PHC center in Obom) A acute ulcers which healed within the observations period B chronic non healing ulcers.

**Fig 5:**
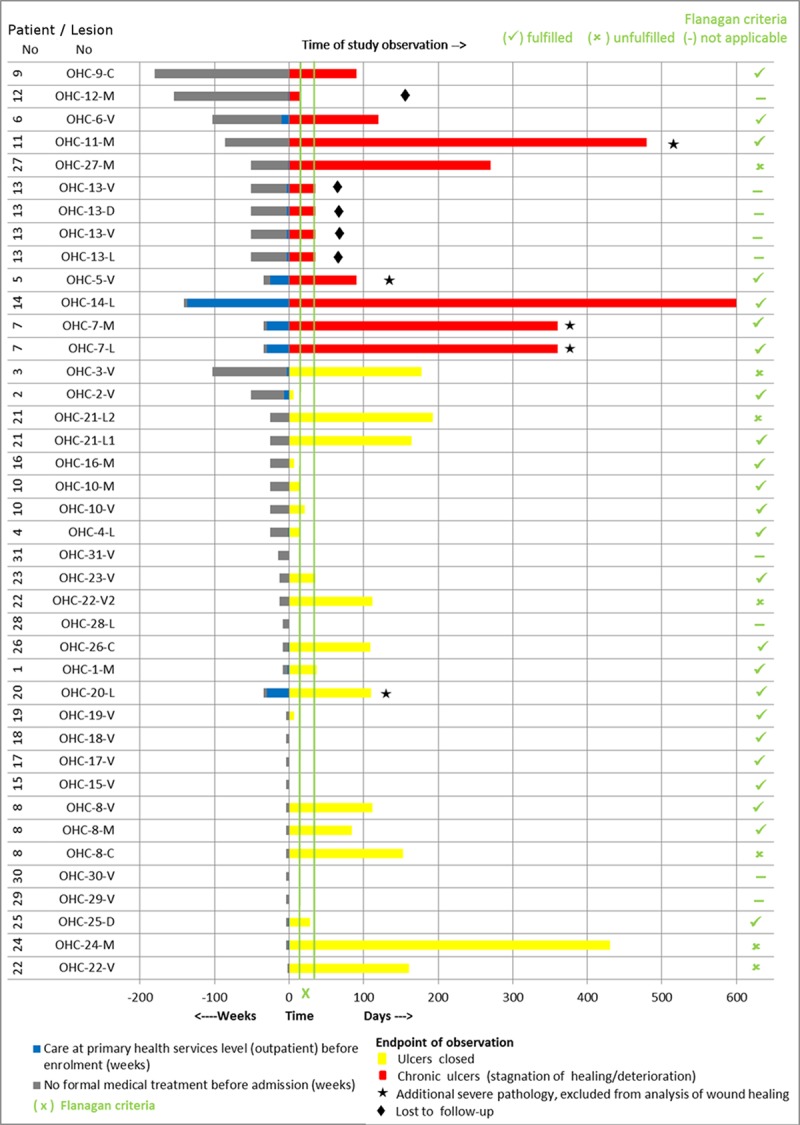
Pre-enrolment history and post-enrolment observation of IS*2404* PCR positive ulcers of the study patients at the PHC center (the patient code “OHC” refers to the patient numbering at the PHC center in Obom).

**Fig 6:**
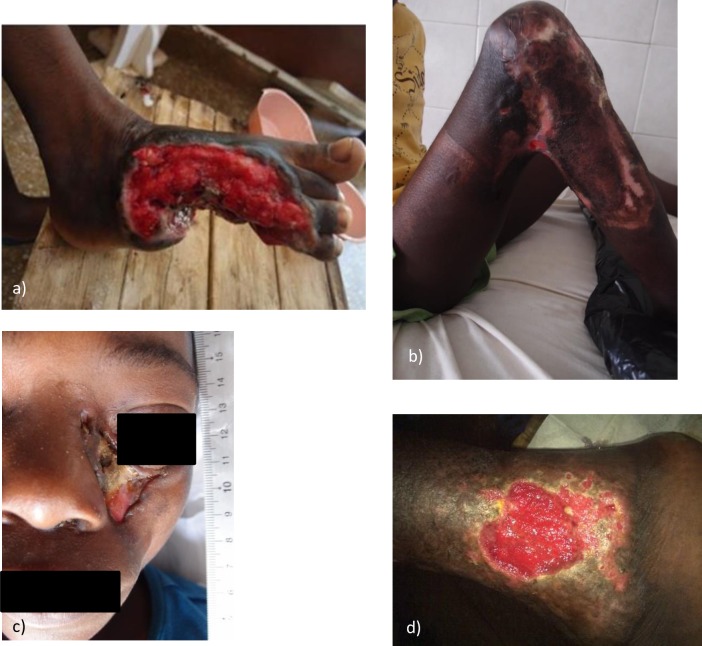
**Complications of IS*2404* PCR positive ulcers** a) squamous cell carcinoma of the right foot in patient 023 at the SHC center b) knee contracture in patient 014 at the SHC center; c) exposed necrotic maxillary bone in patient 015 at the SHC center d) non-healing left lateral malleolar ulcer with underlying chronic lymphedema in patient 007 at the PHC center.

Of the 30 PCR positive skin ulcers assessed at the PHC center, 26 (86.7%) healed completely without the need for surgical or other adjunctive therapy and 4 (13.3%) did not heal during the observation period (Fig 4A and 4B). Sixty-five percent (17/26) of healed skin ulcers healed in less than 3 months, 26.9% (7/26) between 3 and 6 months and 7.7% (2/26) after 6 months. Three out of four non-healing skin ulcers were reliably predicted to not respond to treatment, and fulfilled Flanagan’s criteria (Fig 5). For the 4 PCR positive skin ulcers that failed to heal, underlying problems identified were exposed bone (n = 1), wound infection (n = 1), wound location at a joint (n = 1) and poor adherence to treatment (n = 1). Additional severe pathologies identified amongst the IS*2404* positive skin ulcers were chronic osteomyelitis (n = 2), chronic lymphedema (n = 2) (Fig 6D), and squamous cell carcinoma (n = 1). These ulcers were excluded from the wound healing analysis because the additional severe pathologies found preclude healing (see Fig 1).

**Fig 7:**
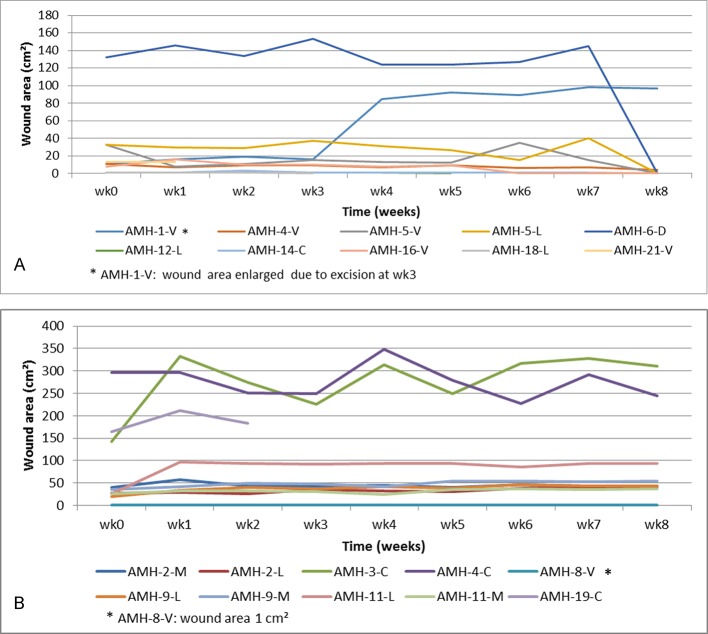
Development of IS*2404* PCR positive ulcers measured by change in wound area over time at the SHC center (the patient code “AMH” refers to the patient numbering at the SHC center in Amasaman) A sub-chronic and healed ulcers B chronic non healing ulcers.

Of the twenty IS*2404* PCR positive skin ulcers assessed at the SHC center, 8 (40%) healed at the endpoint after split-skin grafting (75%), excision (12.5%) and without adjunct treatment (12.5%). Twelve IS*2404* PCR positive ulcers (60%) did not heal during the observation period of which 10 (83.3%) persisted in a chronic state and 2 (16.7%) in a sub-chronic wound state despite regular wound care. Underlying pathologies identified for delayed wound healing were: wound infection (n = 10), venous and arterial insufficiency (n = 4) and nutritional deficiency (n = 7). Some of the patients had more than one possible cause of delayed wound healing. Ninety percent (9/10) of chronic wounds were reliably predicted to not respond to treatment, and fulfilled Flanagan´s criteria. At enrolment the mean wound area for sub-chronic and healed wounds was 22.2 cm^2^ (1–132 cm^2^) and for chronic wounds 78.1 cm^2^ (1–297 cm^2^) (Fig 7A and 7B). Additional severe pathologies identified amongst the IS*2404* positive skin ulcers were osteomyelitis (n = 2) and malignant ulcer (n = 2) (see Fig 6A). These ulcers were excluded from the wound healing analysis because the additional severe pathologies found preclude healing (see Fig 1). Other complications are shown in Fig 6B and 6C.

**Fig 8:**
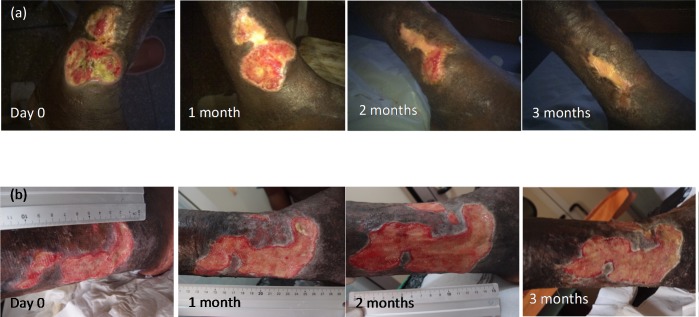
a) Uncomplicated IS*2404* PCR positive ulcer of the left ankle of a patient at the PHC center; b) non-healing IS*2404* PCR positive ulcer of the right leg of a patient at the SHC center.

Fig 8 contrasts an uncomplicated IS*2404* PCR positive ulcer of the left ankle in a PHC center patient with a non-healing IS*2404* PCR positive ulcer of the right leg in an SHC center patient.

**Fig 9:**
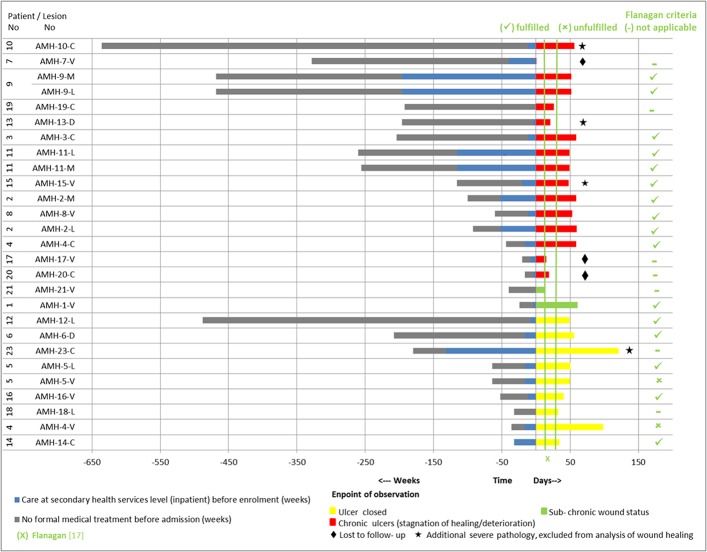
Pre-enrolment history and post-enrolment observation of IS*2404* PCR positive ulcers of the study patients at the SHC center (the patient code “AMH” refers to the patient numbering at the secondary health care center in Amasaman).

Figs 5 and 9 illustrate the history and evolution of IS*2404* PCR positive ulcers during the observation period.

At the PHC center, 66.7% (16/24) of IS*2404* PCR positive ulcers with a pre-enrolment duration of ≤6 months closed within a post-enrolment observation period of ≤3 months. In contrast, only 16.6% (1/6) of IS*2404* PCR positive ulcers with a pre-enrolment duration of >6 months closed within a post-enrolment observation period of ≤3 months. Two of four patients with chronic IS*2404* PCR positive ulcers which did not close whilst under treatment at the PHC center during the study period were lost to follow-up (Fig 5).

The SHC center patients had observed their IS*2404* PCR positive ulcers on average 33 (range 3–156) months before visiting a health facility. At enrolment the patients were already hospitalized on the BU ward or had been treated as outpatients on average for 7 months (range 1–49). Fifty percent (5/10) of the IS*2404* PCR positive ulcers with a pre-enrolment duration of ≤16 months closed within a post-enrolment observation period of ≤3 months. In contrast, only 20% (2/10) of IS*2404* PCR positive ulcers with a pre-enrolment duration of >16 months closed within a post-enrolment observation period of ≤3 months. Three patients with chronic IS*2404* PCR positive ulcers were lost to follow-up after an average observation time of 12 days. (Fig 9).

### Wound infection

**Fig 10:**
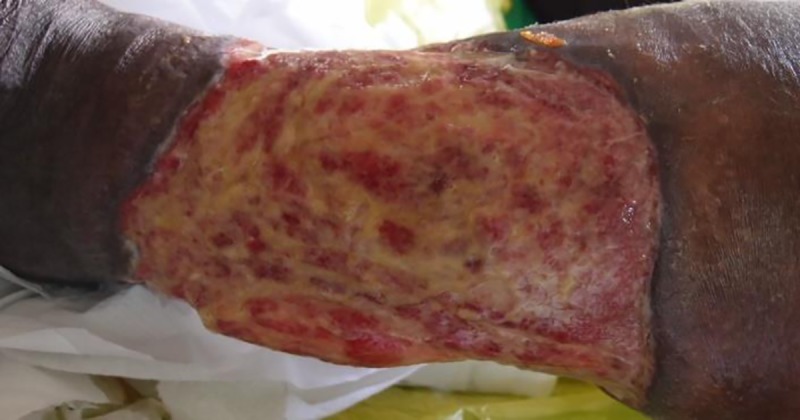
Infected IS*2404* PCR positive ulcers on the left leg of patient 003 at the SHC center.

At the SHC center 50% of all IS*2404* PCR positive ulcers showed evidence of infection at least once during the observation period as compared to 9% at the PHC center. Fig 10 shows a typical case of wound infection with unhealthy, pale-looking granulation tissue, creamish discharge and slightly swollen adjacent skin.

### Pain assessment

**Fig 11:**
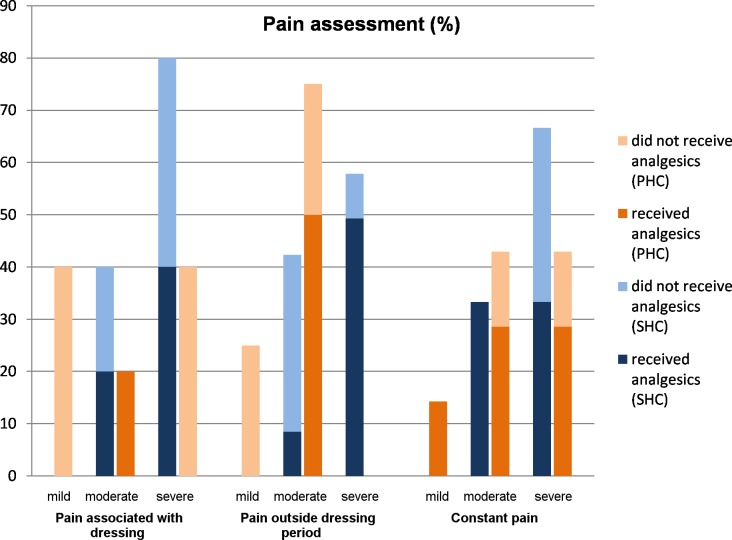
Pain assessment in BU patients with IS*2404* PCR positive ulcers enrolled at the PHC and the SHC centers.

At the PHC center 54.8% (17/31) of patients with IS*2404* positive ulcers experienced pain related to their wounds at least once during the observation period. In 52.9% (9/17) of these patients, the pain was intermittent, usually during wound dressing in 55.6% (5/9) while 47.1% (8/17) had constant pain. Of all patients with wound pain, 23.5% (4/17) described it as mild, 52.9% (9/17) as moderate and 23.5% (4/17) as severe. Of the pain associated with wound dressing, 40% (2/5) was mild, 20% (1/5) moderate and 40% (2/5) severe. None of the patients with wound dressing associated severe pain received analgesics, while 52.9% (9/17) of all patients who experienced pain used analgesics at some point during treatment (see Fig 11). The analgesics used were paracetamol, diclofenac and ibuprofen, all of which were not prescribed.

At the SHC center, 59.1% (13/22) of all patients with IS*2404* positive ulcers (including those with chronic ulcers) enrolled in the study complained about pain related to their wounds. The pain was localized at the wound itself and at the surrounding tissues in (53.8%, 7/13) or on the entire affected limb. Thirty-eight percent (5/13) of patients had wound dressing associated pain but none of the patients received analgesics prior to wound dressing. 53.8% (7/13) of all patients who complained about pain received analgesic therapy at some point (see Fig 11). The most frequently prescribed analgesics were paracetamol, diclofenac or a combination of the two.

### Infrastructure and wound care practices

At the PHC center the facility had one treatment room where all wounds were dressed. These wounds included minor burns, traumatic ulcers, minor surgical wounds and Buruli ulcers. As a measure to prevent possible transmission of *M*. *ulcerans* to other wounds, BU wounds were treated only after all the non-BU wounds had been dressed. Large and complicated wounds were referred to the closest secondary health-care facility in the municipality.

Availability and quality of dressing materials was limited and the observation of wound dressing techniques revealed some shortcomings:

- gloves and instruments (scissors, forceps etc.) were sometimes not available in sufficient quantities
- wound irrigation was hampered by the availability of normal saline and the alternative approach with clean water [17] has so far not been considered
- re-use of bandages after washing in patients’ households was regularly observed for economic reasons
- cleaning of wound surfaces from exudates was mainly mechanical, interfering with granulation
- contamination of wounds from surrounding skin during wound cleaning and dressing has been observed as possible cause of secondary bacterial wound infection
- pain control is not perceived as a very important component of wound management

At the SHC center, separation of out- and in-patients, patients with contaminated, bacterially infected and non-contaminated wounds and wound management following standardised protocols were not fully installed and inconsistent.

In the expert interviews, all experts identified hygiene and wound care deficiencies as a major cause of deterioration of wounds, in addition to a lack of identifying complicating underlying conditions. The wound management recommendations by the local experts were oriented closely at wound care principles of WHO [7]. The international expert highlighted additionally the problem of recognition of arrested wound healing and the lack of progression to active wound management, such as refreshing wound margins.

### Recapture study of patients with IS*2404* PCR negative ulcers at the PHC center

**Fig 12:**
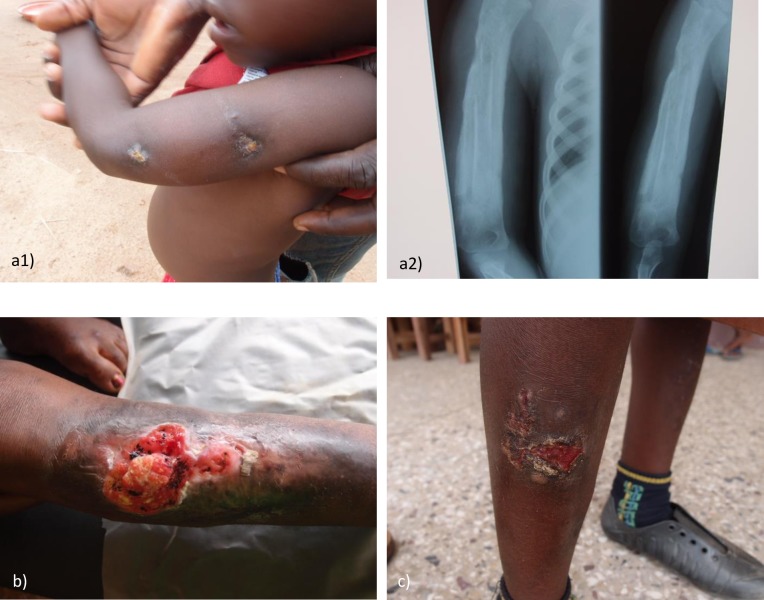
Non-healing IS*2404* PCR negative ulcers at the recapture study at the PHC center: a1) and a2) chronic osteomyelitis; b) suspected malignant ulcer; c) infected wound.

Of the 81 IS*2404* PCR negative skin ulcers of patients recaptured at the PHC center, 4 had additional severe pathologies, chronic osteomyelitis (n = 2) (see Fig 12a1 and 12a2), suspected malignant ulcer (n = 1) (see Fig 12B), and suspected ossified fibroma (n = 1). They were excluded from the wound healing analysis because the additional severe pathologies found preclude healing (see Fig 1).

Of 77 ulcers included in the analysis, 76 (98.7%) were either completely closed (86.8%) or almost closed (13.2%). The remaining 1 non-healing wound was infected (confirmed microbiologically as *Staphylococcus aureus*) (see Fig 12C). All open IS*2404* PCR negative wounds remained negative on repeat PCR (recapture wounds–see S1 Wound documentation Recapture Study).

## Discussion

This is the first comprehensive prospective observational study on wound healing in BU patients with IS*2404* PCR positive ulcers which compares a primary and secondary health care center. Additionally, patients with ulcers clinically diagnosed as BU at the PHC level but not confirmed by IS*2404* PCR were recaptured in the community to verify the correctness of the classification as non-BU cases, to assess the course of wound healing, to make a final diagnosis and to provide treatment, if wounds persisted.

At both the PHC and SHC levels, there were two main categories of IS*2404* PCR positive BU ulcers. One group healed promptly, progressively reducing in size and closing completely within a maximum of 12 weeks after enrolment, whereas the other group was largely unresponsive to the wound management applied. The latter were chronic ulcers already persistent over various lengths of time before enrolment.

The proportion of early versus longstanding, often chronic ulcers differed substantially between the PHC and SHC levels. At the PHC level most (52%) of the IS*2404* PCR positive ulcers were WHO Category I ulcers, whereas at the SHC level the majority (82%) were Category III ulcers. As a consequence, the majority of the ulcers at the PHC level closed without the need for surgical intervention (86.7%) compared to only 40% at the SHC level where the majority required split-skin grafting (75%) or excision (12.5%). This shows clearly that ulcers which have become large or chronic run a very high risk of requiring invasive and expensive therapy to achieve wound closure.

Assessment of healing progress is important to prevent overlooking the transition of ulcers into chronic forms which need active interventions to prevent longstanding arrest and further deterioration. Monitoring of wound surface area is a simple and reliable method. Flanagan has suggested on the basis of an extensive literature review that a reduction of less than 20 to 40% within 2 to 4 weeks after initiation of treatment is a strong indication of failing wound healing [16]. This criterion has been incorporated into the WHO guidelines [7] and we have applied it here to our patients (see Figs 5 and 9). Three out of four of non-healing wounds in the PHC level cohort, and 90% (9/10) at the SHC level were reliably predicted to fail to respond to treatment at the recommended assessment time points, and therefore fulfilled Flanagan’s criteria.

Monitoring reduction in wound area as a sign of healing, faces difficulties during anti-mycobacterial therapy. Ulcers may enlarge during therapy, because the skin covering areas of necrotic subcutaneous tissue may break in and tissue debris may slough off [18]. Furthermore, immune reconstitution-associated paradoxical reactions [10, 19] may contribute to an enlargement of the wounds, leading to an inflammatory phase preceding the wound healing phase. These causes of enlargement of wounds or healing delay often cannot easily be distinguished from persisting *M*. *ulcerans* infection or deterioration caused by secondary infection with other pathogens [9, 10, 19]. In this study, persistence of *M*. *ulcerans* infection after completion of antibiotic treatment was not observed. All IS*2404* positive wounds which failed to close had underlying pathologies or causes in addition to BU. At the PHC level these were chronic osteomyelitis, chronic lymphedema, squamous cell carcinoma and wound infection and at the SHC level, venous and arterial insufficiency, malignant deterioration, nutritional deficiency and lymphedema. Other reasons for impaired wound healing at the SHC level were wound care and hygiene insufficiencies and repeated wound infections. Only 9% of wounds at the PHC level and as much as 50% at the SHC level were clinically infected. This significant difference may be attributable to differences in the wound spectrum cared for as well as the greater risk of acquiring nosocomial infections at the secondary level of the health care system (with in-patient facilities) compared to the primary level. Since all but two of the patients at the SHC level where managed as in-patients, the majority of them had intense daily contact with the hospital environment, in contrast to the PHC level patients who were all managed on an out-patient basis. At the SHC level there was no adequate spatial separation of patients with clean post-surgical wounds from those yet to have surgery and new patients with active ulcers. Thus, patients were under high risk of cross-contamination of wounds resulting in difficult-to-treat wound infections.

When critical deficiencies were observed, the responsible persons of the BU control program and the health services were informed and corrective measures taken, e.g. supply of dressing materials and instruments and discussion with the authorities involved on the need to ensure regular supply of these materials; continuous training of nurses on wound bed preparation techniques and the need to be more sensitive to patient-centered concerns and its impact on wound healing.

In summary, many factors account for failed wound healing. Patient-dependent reasons include underlying conditions interfering with wound healing, such as diabetes mellitus and arterial hypertension, malnutrition, and lack of compliance. On the health provider side it includes infrastructural problems, availability of dressing material, clean water for cleansing of wounds and lack of training and motivation of health staff, which put patients at risk of impaired wound healing and deterioration of wounds, with repeated secondary bacterial infections as the main driving force. We observed various factors facilitating better wound healing and early wound closure at the community level. For obvious reasons, patients tend to present early to community health centers and wounds are thus smaller on presentation compared to patients at the secondary health care level. Successful wound management is achieved with less effort for both the health care system and the patients and at much lower cost. Nosocomial infections can be more easily avoided. Additionally, patients continue to live in their communities which positively impacts on their nutritional status. We observed only 6% underweight patients at the PHC level as compared to 36% at the SHC level.

BU wounds are traditionally regarded to be typically painless unless there is superimposed bacterial infection [3, 4]. In our study, however, the majority of patients (54.8% at the PHC level and 59.1% at the SHC level) experienced wound pain at various points in time, mostly severe and associated with wound dressing (55.6% at the PHC level and 40% at the SHC level). Alferink et al. and de Zeeuw et al. made a similar observation with nearly 30% of BU patients experiencing severe procedural pain [20, 21]. Adequate preparation and planning of procedures such as wound dressing are key to pain prevention [7].

Pain negatively affects wound healing and severely impairs daily life [22]. However, only around half of the patients at both health care levels had access to analgesics. None of the patients received analgesics prior to wound dressing at both health facilities, and the majority of analgesics used by patients were not prescribed. This is avoidable sufferance and a significant deviation from the WHO wound management guidelines [23].

In vertical disease-specific control programs and clinical trials, patients who do not belong to the target group receive little, if any attention. BU is a particularly difficult disease in this respect since many more patients with wounds are clinically suspected to be BU than finally laboratory confirmed. This carries risks in two directions. False negatives do not get the indicated anti-mycobacterial treatment and true negatives may have other significant pathologies in need of specific treatment or require general wound management to prevent transition into chronic ulcers and systemic complications. We therefore re-captured at the PHC level all patients who went back into the community during the study period after clinically suspected BU had not been confirmed by IS*2404* PCR. The aim was to assess the course of wound healing in these patients, to identify patients who have been misdiagnosed and to treat patients with persisting ulcers. Ninety-nine percent (76/77) of the IS*2404* PCR negative ulcers were either completely closed (86.8%) or almost closed (13.2%). This can be explained by the fact that the majority of these patients (60/80; 75%) were children in the 6–15 year group. This is a physically very active age group, and their wounds were likely of traumatic origin. Very importantly, however, 5% of the recaptured patients were found to have missed diagnoses of therapeutic significance (chronic osteomyelitis, wound infection, ossified fibroma and suspected malignant ulcer). All ulcers which were still open at the time of recapture remained negative in repeated IS*2404* PCR analysis.

## Conclusion

After completion of specific treatment, Buruli ulcers often require adequate general wound management over long periods to achieve wound closure. Even where attempts are made to follow WHO recommended standards of care, deficits of health care services and delays in the recognition of complications are continuing bottlenecks to satisfactory outcomes.

This study indicates that with basic infrastructure, equipment and supplies at appropriate quality standards, well-trained health staff and adherence to wound management guidelines, most wounds can be adequately treated at the PHC level, where patients tend to report earlier, stay closer to their families, can maintain their means of livelihood and are less prone to nosocomial wound infections compared to in-patient facilities. Determining reduction in wound area after 2 to 4 weeks (Flanagan’s criteria) of treatment is useful to predict non-healing wounds. Recapture of patients with clinically BU suspicious, PCR negative wounds, clearly showed that even though the majority had healed without sequelae, significant pathology remains unattended if not carefully followed-up. Patient centered care needs to change from vertical to horizontal wound management and facility and training related issues need to be urgently addressed.

## Supporting information

S1 STROBE Checklist (PDF)

S1 Study protocol (PDF)

S1 Case Report Form (CRF) for enrolment (Day 0). (PDF)

S2 Case Report Form (CRF) Follow-up. (PDF)

S3 Case Report Primary Health Care Center (PHC) at Obom (OHC) (PDF)

S4 Case Report Secondary Health Care Center (SHC) at the Hospital Amasaman (AMH) (PDF)

S1 Ethical clearance (PDF)

S1 Wound documentation Recapture Study (PDF)

## References

[1] Röltgen K, Pluschke G. Epidemiology and disease burden of Buruli ulcer: a review. Research and Reports in Tropical Medicine. 2015;6:59–73.

[2] George KM, Chatterjee D, Gunawardana G, Welty D, Hayman J, Lee R, et al Mycolactone: a polyketide toxin from Mycobacterium ulcerans required for virulence. Science (New York, NY). 1999;283(5403):854–7. Epub 1999/02/05. 10.1126/science.283.5403.854 9933171

[3] Junghanss T, Johnson R.C, Pluschke G. Mycobacterium ulcerans disease In: Farrar J, Hotez P, Junghanss T, Kang G, Lalloo D, White NJ, editors. Manson's Tropical Diseases, Expert Consult. 23rd ed. Philadelphia, USA: Elsevier Saunders; 2014 p. 519–31.

[4] World Health Organization. Treatment of mycobacterium ulcerans disease (Buruli ulcer): guidance for health workers, [Adobe PDF, file no. 9789241503402_eng.pdf]. Geneva: World Health Organization, 2012 [2017/01/31]. Available from: http://www.who.int/iris/handle/10665/77771.

[5] Junghanss T, Um Boock A, Vogel M, Schuette D, Weinlaeder H, Pluschke G. Phase change material for thermotherapy of Buruli ulcer: a prospective observational single centre proof-of-principle trial. PLoS Negl Trop Dis. 2009;3(2):e380 Epub 2009/02/18. PubMed Central PMCID: PMC2637542. 10.1371/journal.pntd.0000380 19221594 PMC2637542

[6] Vogel M, Bayi PF, Ruf M-T, Bratschi MW, Bolz M, Um Boock A, et al Local Heat Application for the Treatment of Buruli Ulcer: Results of a Phase II Open Label Single Center Non Comparative Clinical Trial. Clin Infect Dis. 2016;63(3):242–50. Epub 2015 Oct 20. PubMed Central PMCID: PMC4706634. 26486698 10.1093/cid/civ883 PMC4706634

[7] World Health Organization. Wound and Lymphoedema Management Geneva: World Health Organization, 2010 [2017/01/31]. Available from: http://apps.who.int/iris/handle/10665/44279.

[8] Simpson C, O'Brien DP, McDonald A, Callan P. Mycobacterium ulcerans infection: evolution in clinical management. ANZ journal of surgery. 2013;83(7–8):523–6. Epub 2012/09/19. 10.1111/j.1445-2197.2012.06230.x 22989109

[9] Yeboah-Manu D, Kpeli GS, Ruf MT, Asan-Ampah K, Quenin-Fosu K, Owusu-Mireku E, et al Secondary Bacterial Infections of Buruli Ulcer Lesions Before and After Chemotherapy with Streptomycin and Rifampicin. PLoS Negl Trop Dis. 2013;7(5):e2191 Epub 2013/05/10. PubMed Central PMCID: PMC3642065. 10.1371/journal.pntd.0002191 23658847 PMC3642065

[10] O'Brien DP, Robson M, Friedman ND, Walton A, McDonald A, Callan P, et al Incidence, clinical spectrum, diagnostic features, treatment and predictors of paradoxical reactions during antibiotic treatment of Mycobacterium ulcerans infections. BMC Infect Dis 2013;13:416 PubMed Central PMCID: PMC3854792. 10.1186/1471-2334-13-416 24007371 PMC3854792

[11] Schütte D, Um-Boock A, Mensah-Quainoo E, Itin P, Schmid P, Pluschke G. Development of Highly Organized Lymphoid Structures in Buruli Ulcer Lesions after Treatment with Rifampicin and Streptomycin. PLoS Negl Trop Dis. 2007;1(1:e2). Epub 2007/10/31. PubMed Central PMCID: PMC2041817 10.1371/journal.pntd.0000002 17989779 PMC2041817

[12] Nienhuis WA, Stienstra Y, Thompson WA, Awuah PC, Abass KM, Tuah W, et al Antimicrobial treatment for early, limited Mycobacterium ulcerans infection: a randomised controlled trial. Lancet (London, England). 2010;375(9715):664–72. Epub 2010/02/09. 10.1016/S0140-6736(09)61962-0 20137805

[13] Nienhuis WA, Stienstra Y, Abass KM, Tuah W, Thompson WA, Awuah PC, et al Paradoxical responses after start of antimicrobial treatment in Mycobacterium ulcerans infection. Clin Infect Dis. 2012;54(4):519–26. Epub 2011/12/14. 10.1093/cid/cir856 22156855

[14] Portaels F, Silva MT, Meyers WM. Buruli ulcer. Clinics in dermatology. 2009;27(3):291–305. Epub 2009/04/14. 10.1016/j.clindermatol.2008.09.021 19362692

[15] World Health Organisation. Global Database on Body Mass Index 2006 [30. Jan. 2017]. Available from: www.who.int/bmi/index.jsp.

[16] Flanagan M. Improving accuracy of wound measurement in clinical practice. Ostotomy/Wound Management. 2003;(49):28–40. 14652419

[17] Fernandez R, Griffiths R. Water for wound cleansing. Cochrane Database of Systematic Reviews. 2013;(2):Art. No.: CD003861. 10.1002/14651858.CD003861.pub3 22336796

[18] Ruf M-T, Sopoh GE, Brun LV, Dossou AD, Barogui YT, Johnson RC, et al Histopathological changes and clinical responses of Buruli ulcer plaque lesions during chemotherapy: a role for surgical removal of necrotic tissue? PLoS Negl Trop Dis. 2011;5(9:e1334). PubMed Central PMCID: PMC3181242. 10.1371/journal.pntd.0001334 21980547 PMC3181242

[19] Schütte D, Pluschke G. Immunosuppression and treatment-associated inflammatory response in patients with Mycobacterium ulcerans infection (Buruli ulcer). Expert opinion on biological therapy. 2009;9(2):187–200. Epub 2009/02/25. 10.1517/14712590802631854 19236249

[20] Alferink M, de Zeeuw J, Sopoh G, Agossadou C, Abass KM, Phillips RO, et al Pain Associated with Wound Care Treatment among Buruli Ulcer Patients from Ghana and Benin. PloS one. 2015;10(6). Epub 2015/06/01. PubMed Central PMCID: PMCPMC4451111. 10.1371/journal.pone.0119926 PMC4451111 26030764

[21] de Zeeuw J, Alferink M, Barogui YT, Sopoh G, Phillips RO, van der Werf TS, et al Assessment and Treatment of Pain during Treatment of Buruli Ulcer. PLoS Negl Trop Dis. 2015;9(9):e0004076 Epub 2015/09/25. PubMed Central PMCID: PMC4581868. 10.1371/journal.pntd.0004076 26402069 PMC4581868

[22] World Union of Wound Healing Societies. Principles of best practice: Minimising pain at wound dressing-related procedures. A consensus document. London, UK Medical Education Partnership Ltd, 2004 [2017/01/31].

[23] World Health Organization. WHO's cancer pain ladder for adults [2017/01/31]. Available from: www.who.int/cancer/palliative/painladder/en

