# Supplementary material for: Assessing and managing wounds of Buruli ulcer patients at the primary and secondary health care levels in Ghana
Source: PLoS Negl Trop Dis. 2017 Feb 28;11(2):e0005331. doi: 10.1371/journal.pntd.0005331 (PMC5345880; doi:10.1371/journal.pntd.0005331)
Supplement: S1 Study protocol — (PDF) [file pntd.0005331.s002.pdf]

Study protocol

Diagnosis and systematic follow up of Buruli Ulcer patients in a district hospital and in a health Centre

Version: 7.0

Date: 22.08.2013

Subproject of TOWARDS EFFECTIVE CONTROL OF BURULI ULCER 2 (Stop Buruli 2)

NMIMR STC Number: 3(4)/2011-12

NMIMR-IRB CPN 099/11-12

Principal Investigator
Dr. Dorothy Yeboah-Manu, PhD
Department of Bacteriology
Noguchi Memorial Institute for Medical Research
Legon, Accra, Ghana
0208123882 (cell), 0302502152 (fax)
Dyeboah-manu@noguchi.mimocm.org

Dr. Edwin Ampadu
National Buruli ulcer Control Program
Disease Control Unit
Korle-Bu
0208167962

Prof. Dr. Thomas Junghanss
Section Clinical Tropical Medicine
University Hospital, INF 324
69120 Heidelberg, Germany

Prof. Dr. Gerd Pluschke
Medical Parasitology and Infection Biology
Swiss Tropical and Public Health Institute
Socinstr. 57
4002 Basel, Switzerland

DURATION: 2 years

Source of Funding: Stop Buruli and VW Foundation

Table of Contents

NMIMR STC Number: 3(4)/2011-12 1
GLOSSARY AND ABBREVIATONS 1
KEYWORDS 1
SUMMARY OF INVESTIGATION AND ASSESSMENTS 2
1 INTRODUCTION 5
1.1 Background 5
1.2 Rationale for the study 5
1.3 Overall goal 7
1.4 Study objectives 7
2 STUDY DESIGN 7
3 PARTICIPANT ENTRY 8
3.1 Study Site and Target Population 8
3.1.1 Study Site 8
3.1.2 Target Population 8
3.2 Inclusion Criteria 8
3.3 Exclusion Criteria 8
3.4 Pre- enrolment Evaluation 9
3.5 Withdrawal Criteria, Procedures after Withdrawal 9
4 ASSESSMENT AND FOLLOW-UP 9
4.1 Clinical and Laboratory Assessments (at enrolment) 9
4.2 Clinical and Laboratory Assessments during Follow up 10
4.3 Storage, Transport and Analysis of Samples (biopsies, swabs and blood samples) 10
5 ADVERSE EVENTS 11
5.1 Lab procedures 11
6 STATISTICS AND DATA ANALYSIS 12
7 ETHICS AND REGULATORY ISSUES 13
7.1 Ethics Approval 13
7.2 Consent 13
7.3 Confidentiality 14
8 STUDY MANAGEMENT 14
9 REFERENCES 16

GLOSSARY AND ABBREVIATONS

AE – Adverse Event

AFB – Acid Fast Bacilli

BU – Buruli Ulcer, Buruli Disease

CNS – Central Nervous System

CRF – Case Report Form

GCP – Good Clinical Practice

IMP - Investigational Medicinal Product

PCR – Polymerase Chain Reaction

SAE – Serious Adverse Event

ZN – Ziehl-Neelsen

KEYWORDS

Buruli Disease, Wound Management, Wound healing

SUMMARY OF INVESTIGATION AND ASSESSMENTS

Figure 1: Overview of the two study sites

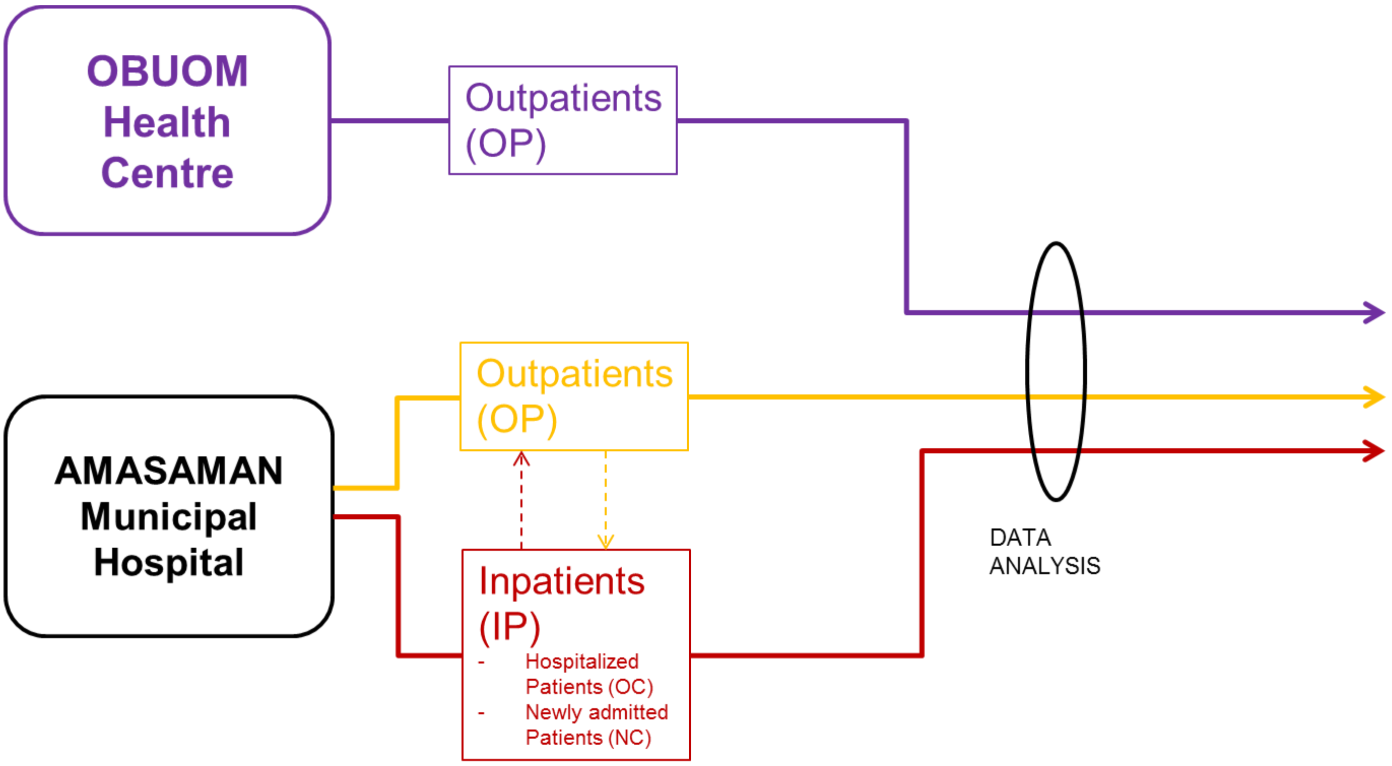

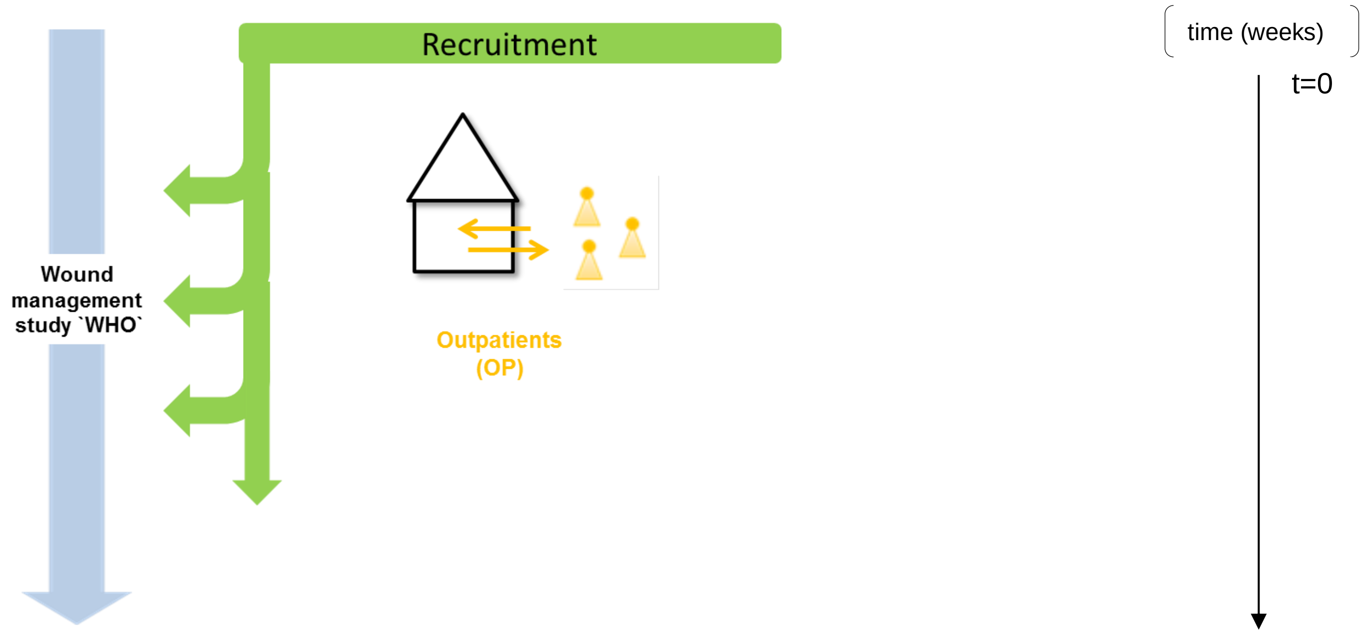

Figure 2: Observational study phases and study groups in Obom

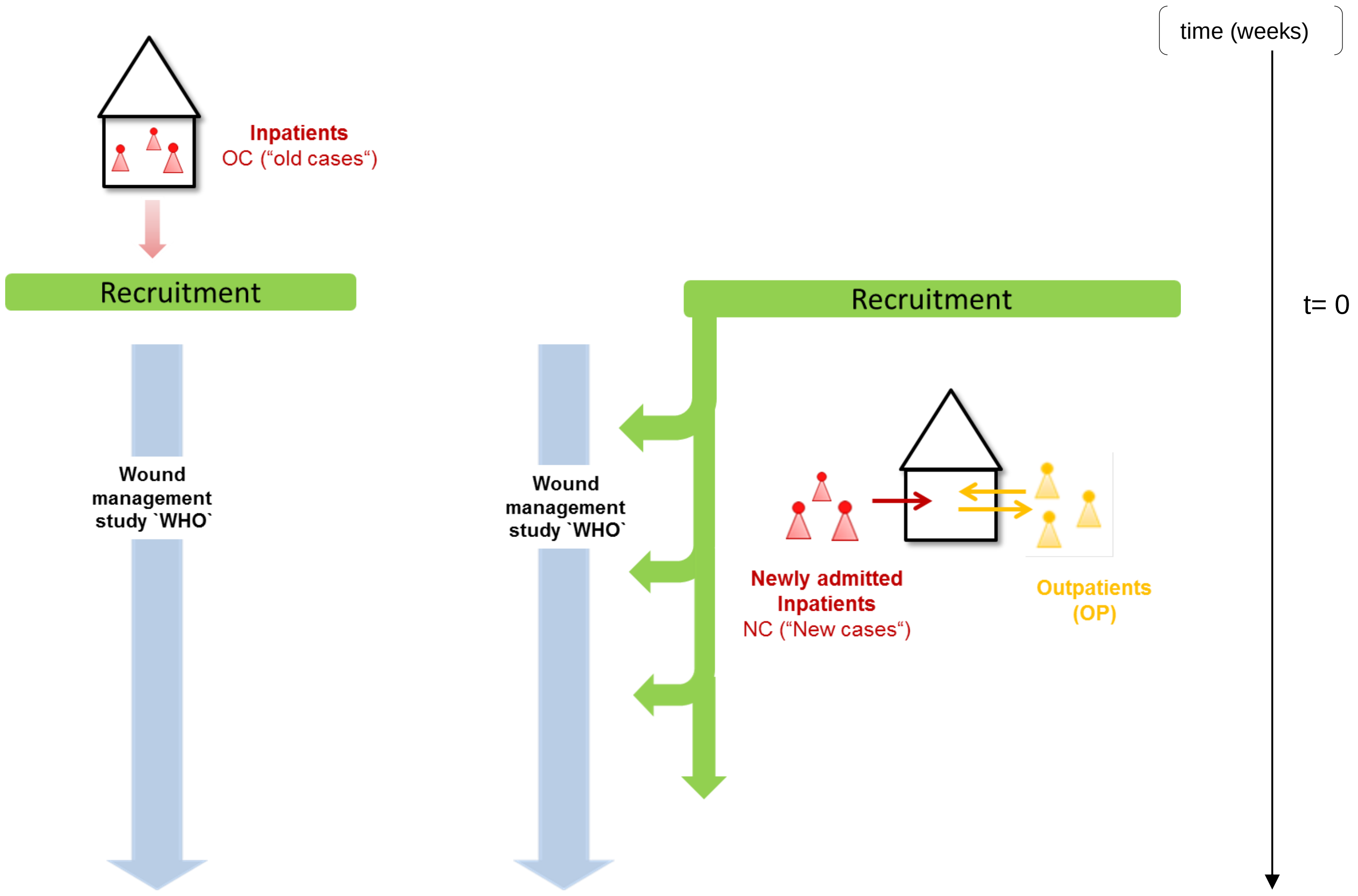

Figure 3: Observational study phases and study groups in Amasaman

	Day0	Month 1	Month 2	Month 3	Month 4	Month 5	Month 6
Clinical examination at enrolment	X						
Informed consent	X						
• Clinical wound assessment<br>• Weight<br>• Temperature	X	Once a week and if indicated	Once a week and if indicated	Every two weeks and if indicated	Every two weeks and if indicated	Every two weeks and if indicated	Every two weeks and if indicated
Photo documentation of wound	X	Every two weeks	Every two weeks	Every two weeks	Every two weeks	Every two weeks	Every two weeks
Swabs (ulcer) for diagnosis (PCR, AFB, culture, AG- test)	X						
FNA (nodule) for diagnosis (PCR, AFB, culture, AG-test)	X[1]						
Swabs (ulcer) for secondary bacterial infection and resistance testing	X	after 2 weeks + after 4 weeks	If indicated	If indicated	If indicated	If indicated	X
Punch biopsy	X		X[2]				
Blood test (WBC, differential, HIV-test[3], sickle test[4])	X						
Blood test (WBC, differential, CRP, malaria, blood culture and others)	If clinically indicated						

[1]if clinically indicated punch biopsy at one additional later date

[2] additional biopsies if indicated and during surgery.

[3] if positive: CD 4 count, viral load, repeatedly if clinically indicated

[4] if positive: referral to a sickle cell disease centre

Table 1 Time schedule

STUDY PROTOCOL

1 INTRODUCTION

1.1 Background

Buruli Disease (BU) is a chronic necrotizing disease of the skin and soft tissue caused by *Mycobacterium ulcerans* [[1]]. The disease starts as a subcutaneous nodule, papule or plaque that eventually ulcerates and progresses over months to years. The disease can affect all age groups, but children under 15 years of age are predominantly affected [[2]]. Serious permanent disability may result. *M. ulcerans* produces a macrolide toxin, mycolactone, which is associated with tissue destruction and local immunosuppression [[3]]. BU has been reported in >30 countries, but the major burden lies on children living in remote areas of West Africa associated with swamps and stagnant water bodies [[4]]. Over decades wide excision of the infected tissue was the standard treatment for BU. Disadvantages are the highly traumatic nature of the interventions, high cost and very high recurrence rates. Chemotherapy with streptomycin/ clarithromycin and rifampicin has successfully been tested in a recent trial and further trials are ongoing [[5]]. Chemotherapy has become an essential part of the treatment recommendations of the WHO BU Technical Advisory Group [[6]] and is currently implemented in BU endemic regions by national BU control program.

In the treatment of BU patients general wound management has been grossly neglected. The focus has been largely on the *M. ulcerans* specific anti-mycobacterial treatment. Problems of wound healing due to local immune reconstitution (paradoxical reactions) and intervening secondary bacterial infections have been misinterpreted and underestimated. Their impact on time to healing (wound closure) and morbidity is significant. This can only be reduced through improvement of general wound care on the basis of better knowledge of the underlying processes. Transferability of results generating in BU patients with chronic wounds to other types of wounds will be high.

1.2 Rationale for the study

To contribute to a better understanding of wound healing and to improve wound management in settings with limited resources, a prospective observational follow-up study of BU patients with chronic wounds will be conducted.

A specific focus will be to disentangle the three principle mechanisms which drive the wound pathology in otherwise healthy patients: (a) the mycobacterial infection, including remaining mycolactone levels in the affected tissue (b) local immune reconstitution and (c) secondary bacterial infections. On the basis of improved knowledge of these entities and their time course clinical recognition can be improved, rational laboratory diagnostics suggested and wound care procedures tailored to the needs and health care settings.

The study will be conducted in the Buruli Ulcer Ward at Amasaman Municipal Hospital and in the Health Centre of Obom. Studying patients in both facilities will allow comprehensive insight in the management and healing of Buruli Ulcer wounds at the primary and secondary level of the health care system.

STUDY OBJECTIVES

1.3 Overall goal

To contribute to a better understanding of wound healing and to improve wound management in settings with limited resources at the primary and secondary level of the health care services.

1.4 Study objectives

(1) To establish the clinical and laboratory tools for monitoring BU patients with chronic wounds.

(2) To initiate a prospective cohort of BU patients with continuous enrolment at Amasaman District Hospital and Obom health Centre.

(3) To describe BU lesions including secondary effects (secondary bacterial infections; local immune reconstitution; emergence of secondary lesions) at enrolment and over time until the wound is closed (or the 6 months study period is completed) for

A.) Hospital based setting

B.) Health Centre based setting

(4) To investigate the microbiological, histopathological and immunological features including wound healing parameters at enrolment and over time until the wound is closed (or the 6 months study period is completed) for

A.) Hospital based setting

B.) Health Centre based setting

2 STUDY DESIGN

Prospective observational follow-up study of Buruli Ulcer patients treated in:

A.) The Buruli Ulcer Ward at the Municipal Hospital of Amasaman

- inpatients already hospitalized at day 0 (“old cases”) (n_{OC}=30)
- inpatients newly admitted (“new cases”) during study period (n_{NC}=30)
- outpatients (n_{OP}=20)

B.) The Health Centre of Obom

- outpatients (n_{OP}=50)

Study duration

2 years

3 PARTICIPANT ENTRY

3.1 Study Site and Target Population

3.1.1 Study Site

The study will take place in

A.) The Buruli Ulcer Ward at Amasaman Municipal Hospital (AMH)in the Ga West District of the Greater Accra Region
B.) The Obom Health Centre (OHC) in the Ga- South Municipality of the Greater Accra region.

Both institutions are the main health care facilities that offer Buruli ulcer case management services in the respective district; both offer clinical diagnosis, wound dressing and Streptomycin/ Rifampicin treatment according to WHO recommendations, while AMH in addition has facilities for surgical excision and skin grafting. The two districts are the only districts that are BU endemic in the Greater-Accra Region and annually attend to more than hundred cases. The Ga-West Municipal Health Directorate reports the second highest number of BU cases in Ghana; furthermore it receives the worst cases nationwide. The Amasaman Municipal Hospital has both in-patient (consisting of three wards and 40 beds capacity) and out-patient facility while the Obom Health Centres has only out-patient facility. Both health care facilities have a longstanding collaboration with the Noguchi Memorial Institute for Medical Research (NMIMR), Legon, for case confirmation by PCR and also offer additional laboratory investigations when needed to support case management.

3.1.2 Target Population

BU patients of the Buruli Ulcer Ward / OPD at Amasaman District Hospital in the Ga West District of the Greater Accra Region and BU patients of the Health Centre at Obom.

3.2 Inclusion Criteria

A.) In- and out-patients with confirmed BU lesions (ulcers / nodules) at Amasaman District Hospital.
B.) Outpatients with confirmed BU lesions (ulcer/ nodules) at the Health Centre of Obom

3.3 Exclusion Criteria

Since no significant adverse effects are foreseen for patients participating in this observational study, no specific exclusion criteria are applicable.

3.4 Pre- enrolment Evaluation

Patients fulfilling the inclusion criteria (see section “inclusion criteria” above) will be fully informed about the clinical study and informed written consent of patients or their representatives willing to participate will be obtained (see section 8.2).

All patients who have consented will be submitted to following pre-enrolment evaluations

(1) Clinical evaluation of the ulcers following the WHO guidelines (WHO 2002)

(2) Medical history

(3) Full physical examination

Patients who are not enrolled will be receiving the same standard care as all patients of the Amasaman Buruli Ulcer Ward / OPD or the Health Centre of Obom.

3.5 Withdrawal Criteria, Procedures after Withdrawal

Patients can withdraw at any time form the study and will continue to receive the standard care of the Amasaman Buruli Ulcer Ward.

4 ASSESSMENT AND FOLLOW-UP

4.1 Clinical and Laboratory Assessments (at enrolment)

A) Buruli Ulcer Ward at Amasaman Municipal Hospital

- Baseline clinical examination including photographic documentation
    - Overall clinical assessment (past and present medical history, present complaints, physical examination)
    - Description of the lesion (margin of ulcer, size of ulcer / nodule [max / min diameter, size in mm^2 determined in digital images taken in a standardized format], wound exudates, epithelialisation, scarring, temperature at lesion and contra lateral)
- Laboratory tests
    - Fine needle aspiration (nodules) for Buruli Ulcer diagnosis
    - Swab samples (ulcers) for diagnosis, validation of new diagnostic procedures and for the analysis of secondary infections and for further necessary analysis
    - Punch biopsies (ulcers) for assessment of histopathological features, nature and localization of secondary infections and for markers of inflammation and wound healing
    - Blood samples for WBC, differential, HIV- test, sickle test
    - Blood samples of patients if clinically indicated for blood cultures, malaria RDT, CRP

- Blood samples from HIV positive patients for CD4 count and viral load

B) Health Centre in Obom:
Same as above

4.2 Clinical and Laboratory Assessments during Follow up

Follow-ups are performed at the intervals as lined out in table 1:

A) Buruli Ulcer Ward at Amasaman Municipal Hospital

- Clinical examination and assessment
    - Clinical assessment (body temperature, weight)

- Assessment of wound healing and secondary effects
    - Description of the lesion (size of ulcer / nodule [max / min diameter, size in mm^2 determined in digital images taken in a standardized format], epithelialisation, scarring, moisture balance, wound ground, granulation tissue, wound exudates, margin of ulcer, temperature at lesion and contra lateral, wound surface, odour, pus)
    - Photo documentation made of all lesions.

- Laboratory tests
    - Punch biopsies (ulcers) for assessment of histopathological features, nature and localization of secondary infections and for markers of inflammation and wound healing treatment success
    - Swab samples (ulcers) for analysis of secondary infections
    - Blood samples of patients with a temperature >38,5°C or clinically indicated for blood cultures, malaria RDT, WBC, differential blood count, CRP and others
    - Blood samples from HIV positive patients for repeated CD4 count and viral load
    - Tissue samples (of wound debridement, wound excision) for assessment of histopathological features

B) Health Centre in Obom: As above

All data will be directly entered into the CRF (source data)

4.3 Storage, Transport and Analysis of Samples (biopsies, swabs and blood samples)

Four swabs will be collected for investigating secondary infection; one will be used for direct smears for microscopic examination, the second inserted into a sterile tube containing 3 ml of PBS for

enumeration of bacterial load and isolation of specific aerobic pathogens, the third will be transported in Robertson's cooked meat medium for anaerobic bacteria isolation and the fourth will be used for cultivation of *M. ulcerans* and/or determination of mycolactone levels. Samples will be kept on ice and transported the same day to the laboratory at NMIMR for investigations the same day, if delay is anticipated, then samples will be collected into Amies transport media.

Biopsy samples for histopathological analysis will be inserted into 10% formalin for 24 hours, and then transferred into a new tube containing 70% ethanol, until analysis at Swiss Tropical and Public Health Institute in Basel, Switzerland. For the determination of mycolactone levels, biopsy samples will be immersed in chloroform/methanol and transported the same day to the laboratory at NMIMR for extraction.

Blood samples for microbiological analysis (for an adult, 10-15ml of blood is collected and 5ml for paediatric) will be collected directly into a diphasic blood culture bottles and transported within 24 hours to the laboratory for culture and isolation while blood for CD4 counts and viral load will be collected into vacutainer tubes (BD).

5 ADVERSE EVENTS

The clinical observational study procedures do not pose any risk to the patient.

5.1 Lab procedures

Enumeration of Bacteria Burden: The swab in 3ml PBS will be vortexed and using the stock suspension, serial dilutions from 10^{-2} to 10^{-8}. Hundred microlitres of serial dilutions will be transferred into sterile Petri dishes and inoculated by the pour plate method using Plate Count Agar for total aerobic counts (oxoid) after incubation at 37°C for 18-24 hours. After incubation, the plates will be examined using a colony counting chamber (Gallenkamp, UK) and those with colony counts between 30 and 300 will be selected for computing CFU/ml by multiplying the counts by the dilution factors. The lesion from which the sample was taken will then be classified as clean, contaminated or infected.

Isolation of Specific Aerobic Pathogens: The stock solution will then be centrifuged at 8,000g for 30 minutes and the sediment will be cultured under aerobic conditions on blood, chocolate and MacConkey agar for bacteria isolation. Isolates will be identified by specific biochemical analysis following gram staining and confirmed by MALDI-TOF mass spectrometry.

Anaerobic Bacteriology: The sample in the cooked meat medium will be incubated at 37°C for 48-73 hours. The media will then be examined for signs of growth such as turbidity, proteolysis and saccharolysis. Tubes with signs of growth will then be subcultured onto blood agar with a metronidazole disc placed on it to check for resistance. This is placed in a glass jar with an anaerobic

gas pack and incubated at 37°C for 48-72 hours; bacteria growth will be examined for colonial morphology and susceptibility to metronidazole and gram reaction. Final identification will be done by MALDI-TOF mass spectrometry.

Blood Culture: The culture bottles will be incubated aerobically at 35- 37°C for up to 7 days and daily checked for signs of growth such as turbidity of the broth, growth on the agar slope or a pellicle on the surface of the broth. Subculture will be done onto Blood agar (35-37°C for 18-24 hours aerobically), Chocolate agar (35-37°C for 48 hours anaerobically) and MacConkey agar (37°C for 18-24 hours aerobically). Growth will be identified using gram staining and standard biochemical analysis depending on the gram reaction.

Mycolactone analysis: Extracted lipids will be analyzed by liquid chromatography-mass spectrometry (LC-MS).

Histopathology: Tissue samples will be fixed in 4% neutral-buffered paraformaldehyde for 24h and subsequently transferred to 70% ethanol for transport. Samples will be dehydrated, embedded in paraffin, cut into 5 mm thin sections and retrieved on glass slides. After dewaxing and rehydration, sections will be stained with haematoxylin/eosin (HE) and Ziehl-Neelsen (ZN) staining of AFBs. Immunohistochemistry will be performed with antibodies specific for markers of inflammation and wound healing

6 STATISTICS AND DATA ANALYSIS

Descriptive

Simple descriptive statistics will be performed with baseline variables (age, sex) and wound healing related variables (clinical response, adverse events, histopathological, microbiological and immunological parameters and features).

1. Age and sex distribution of BU patients hospitalized/attending outpatient clinic in Amasaman and Obom
2. Residence of the BU patients attending the BU Centre Amasaman/ Obom
3. Nutritional status of BU patients
4. Frequency and types of skin diseases coexisting with BU
5. Frequency of BU lesions among relatives/ family members
6. Secondary bacterial infection/ colonization at hospital entry

Analytical

1. Association of histophathological and immunological parameters of wound healing with clinical signs for wound healing
2. Secondary bacterial infection / colonization over time in hospitalized patients
3. Time dependent evaluation of wound size
4. Secondary bacterial infection / colonization of wounds of NC compared to OC
5. Association between body temperature and microbiological colonisation of BU wounds
6. Evaluation of pain during wound healing
7. Definition of clinical wound healing signs in BU wounds over time
8. Determination of patient-related factors predisposing to wound infections and delayed wound healing.

7 ETHICS AND REGULATORY ISSUES

7.1 Ethics Approval

This study will be conducted in accordance with the ethical principles that have their origin in the Declaration of Helsinki and in compliance with ICH-GCP, ISO 14155-1 and -2, and the applicable laws and regulations of the participating country.

Approval of this protocol will be sought for from the:

> Ethical Review Board of the Noguchi Memorial Institute for Medical Research, Legon, Ghana and of the Ghana Health Service

7.2 Consent

Patients willing to participate in the study will be fully informed about the study procedures, the risks of participation and possible benefits. The explanation of the written English informed consent will be translated into the patient's spoken language when needed. In general, written informed consent will be obtained by the signature of the patient or the signature of the guardian/legal representative (in case a patient is minor) on the informed consent form or, if the patient or guardian/ legal representative is illiterate, certify their consent with a fingerprint accompanied by the signature of an impartial witness.

.

The right of the patient or guardian/legal representative to refuse participation without giving reasons will be respected.

All participants are free to withdraw their consent to participate in the study at any time without giving reasons and without any consequences.

The Ethics Committee(s) must approve the informed consent document to be used by the Investigator.

7.3 Confidentiality

The Principal Investigator will preserve the confidentiality of participants taking part in the study.

8 STUDY MANAGEMENT

The day-to-day management of the study will be taken care of by the clinical investigators:
Dr Victor Akuoko and Stefanie Pfau (clinical); Evelyn Owusu-Mireku (Nurse), Grace Kpeli (laboratory) at Amasaman
Naa Okaikor Addison (clinical and microbiological investigations) at Obom

Principal Investigator:
Dorothy Yeboah- Manu (Noguchi Memorial Institute for Medical Research, Legon)

9 REFERENCES

[1]Portaels F, Silva MT, Meyers WM (2009) Buruli ulcer.ClinDermatol 27: 291–305. doi:10.1016/j.clindermatol.2008.09.021
[2] Asiedu K, Scherpbier R, Raviglione M (2000) Buruli ulcer. World Health Organization, Global Buruli Ulcer Initiative.
[3]Werft van der T., Graaf van der W.T.A, Tappero J.W et al (1999): Mycobacterium ulcerans infection in The Lancet 1999; 354: 1013- 1018
[4] Asiedu K, Scherpbier R, Raviglione M (2000) Buruli ulcer. World Health Organization, Global Buruli Ulcer Initiative.
[5]Nienhuis W.A., Stienstra Y., Thompson W.A. et al (2010): Antimicrobial treatment for early, limited Mycobacterium ulcerans infection: a randomused controlled trial in The Lancet 2010; 37: 664-672
[6]Buntine J. WHO (2001): Buruli Ulcer: Management of Mycobacterium ulcerans disease.
