# Supplementary material for: Assessing and managing wounds of Buruli ulcer patients at the primary and secondary health care levels in Ghana
Source: PLoS Negl Trop Dis. 2017 Feb 28;11(2):e0005331. doi: 10.1371/journal.pntd.0005331 (PMC5345880; doi:10.1371/journal.pntd.0005331)
Supplement: S1 Case Report — (PDF) [file pntd.0005331.s003.pdf]

BU-WOUND-STUDY	DAY 0	Side Code ☐☐☐	Patient´s NO ☐☐☐

Date: __. __. __ (dd/mm/yy)

Demographic data

Informed consent form	① signed

Sex: ① Male ② Female

Date of birth: __ __.__ __.__ __ (dd/mm/yy) if unknown, age in complete years: __ __

Place of birth: ______________________________

Home village/ district: ______________________________

Residence: ______________________________

Profession / current job: ______________________________

Educational background	① Yes	⓪ None	
If yes please indicate the level	① Primary	⓪ Secondary	⑧ Tertiary
Religion	① Christian	⓪ Moslem	⑧ Traditional
	② Others:		
Hospital status	① hospitalized, since: __.__.__ (dd.mm.yy)	⓪ outpatient, since: __.__.__ (dd.mm.yy)	⑧ newly admitted

Present medical history

Diabetes	① Yes	⓪ No	⑧ Unknown
TB	① Yes	⓪ No	⑧ Unknown
Hepatitis	① Yes	⓪ No	⑧ Unknown
Congestive heart failure	① Yes	⓪ No	⑧ Unknown
Sickle cell disease	① Yes	⓪ No	⑧ Unknown
HIV	① Yes	⓪ No	⑧ Unknown
Hearing impairment/ disorder	① Yes	⓪ No	⑧ Unknown
Cough	① Yes	⓪ No	⑧ Unknown
Allergies	① Yes	⓪ No	⑧ Unknown
	if yes, please specify:		
Other diseases	① Yes	⓪ No	⑧ Unknown
	if yes, please specify:		
Drugs currently taken	① Yes	⓪ No	⑧ Unknown
	If yes, please list the names of the drugs :		
Pregnancy	① Yes	⓪ No	⑧ Unknown
Smoker	① Yes	⓪ No	
	if yes, please specify: __ __ sticks/day		for __ __ years
Alcohol	① daily	⓪ No	⑧ occasionally

BU-WOUND-STUDY	DAY 0	Side Code ☐☐☐	Patient´s NO ☐☐☐

Physical examination

Weight: __ __ __ kg Height __, __ __ m

Body temperature (axillary): __ __, _ °C

Skin -Ulcer(s) / nodule(s) fill in separate form(s)

Paleness (palms)	① Yes	⓪ No
Jaundice (sclera)	① Yes	⓪ No
Rash	① Yes*	⓪ No
Oedema	① Yes*	⓪ No
Scar(s)	① Yes*	⓪ No
BCG- vaccination scar	① Yes	⓪ No
Pigmentation abnormalities	① Yes*	⓪ No
Varicose veins	① Yes*	⓪ No
Others	① Yes	⓪ No
	If yes, please specify:	

* if yes fill in separate form

Lymph nodes

Palpable	① Yes	⓪ No
If yes:	① generalized	② head/ neck
	③ armpit	④ grain
Painful on palpation	① Yes	⓪ No
Skin above palpable lymph node warmer compared to surrounding skin	① Yes	⓪ No

Cardiovascular system

Pulse	__ __ __ / minute	
Blood pressure	systolic __ __ __ mm Hg	diastolic __ __ __ mmHg
Heart murmur	① Yes	⓪ No
	if yes, specify:	

Respiratory system

① abnormalities detected if yes, specify__________________

⓪ No abnormalities detected

BU-WOUND-STUDY	DAY 0	Side Code ☐ ☐ ☐	Patient´s NO ☐ ☐ ☐

Abdomen

Liver palpable	(1) Yes	(0) No
	if yes, size __ __ cm below costal margin	
Spleen palpable	(1) Yes	(0) No
	if yes, size __ __ cm below costal margin	
Abdomen tender on palpation	(1) Yes	(0) No
	if yes, specify:	
Other abnormalities (ascites)	(1) Yes	(0) No
	If yes, specify:	

CNS

Disorientation	(1) Yes	(0) No
	if yes, specify:	
Motor dysfunction	(1) Yes	(0) No
	if yes, specify:	
Gait deviation	(1) Yes	(0) No
	if yes, specify:	

BU-WOUND-STUDY	DAY 0	Side Code ☐ ☐ ☐	Patient´s NO ☐ ☐ ☐

Lesion general information

**Ulcer(s)** ○ single ○ multiple	**Nodule(s)** ○ single ○ multiple

Site of ulcer(s) / nodule(s)

(sketch and number all lesions and fill in one CRF per lesion)

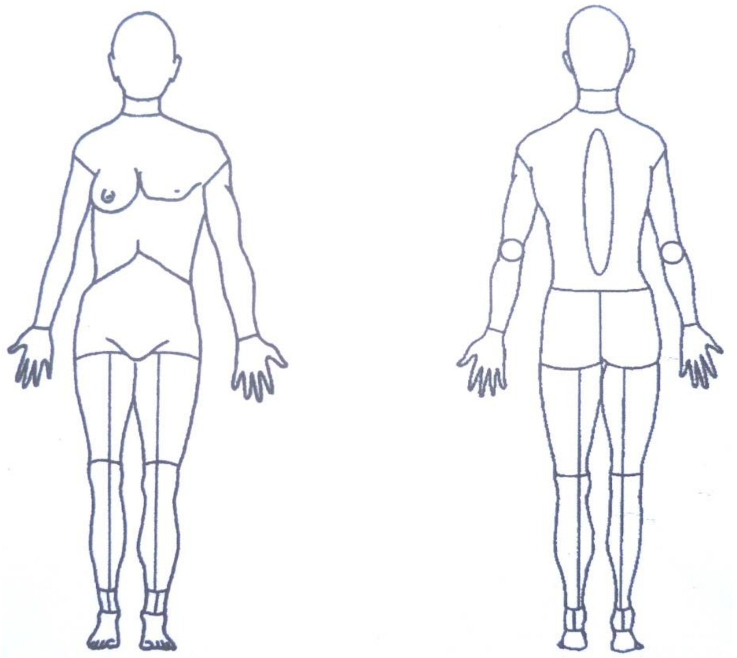

Ulcer/ nodules in family members?	(1) Yes, specify:______	(0) No
Ulcer/nodules among other household member?	(1) Yes, specify:______	(0) No
Patient's expectations of the end point of treatment	(1) complete healing of ulcer	(0) complete function of limb
	(8) odour management	(2) other:
Patient´s expectation of treatment time	__ __ years __ __ months __ __ days	

BU-WOUND-STUDY	DAY 0	Side Code ☐☐☐	Patient´s NO ☐☐☐

Lesion specific

ulcer ☐ nodule ☐	ulcer / nodule localization: __ __

History

observed since __ __. __ __. __ __ (dd/mm/yy)   If unknown, duration in years __ __ months __ __ weeks __ __

Consultation in a health facility before	(1) Yes	(0) No
If yes, please specify:		
Wound dressing	(1) Yes	(0) No
Surgery	(1) Yes	(0) No
Antimycobacterial agents	(1) Yes	(0) No
	if yes, specify:	
Other drugs/ herbals	(1) Yes	(0) No
	If yes, specify:	
Traditional treatment	(1) Yes	(0) No
	If yes, specify:	
History of trauma at site of lesion?	(1) Yes	(0) No
	if yes, specify	

Present complaints

Pain	(1) Yes*	(0) No

*if yes, specify in the visual analog scale (VAS):

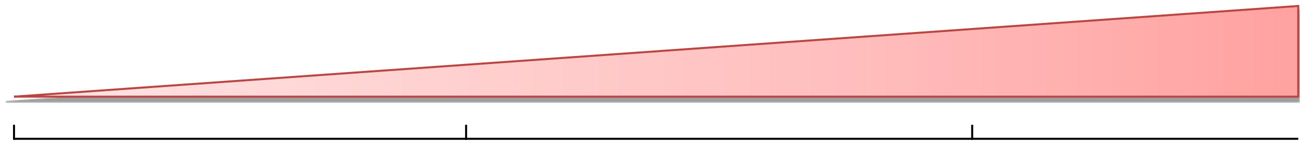

mild pain (1)   moderate pain (2)   severe pain (8)

Itching	(1) Yes	(0) No
Restriction of movement	(1) Yes	(0) No
Others	(1) Yes	(0) No
	if yes, specify:	

Size

Max. diameter: __ __ __ mm   Area of the wound __ __ __ mm²

Max. diameter: __ __ __ mm   Area of the induration __ __ __ mm²

BU-WOUND-STUDY	DAY 0	Side Code ☐ ☐ ☐	Patient´s NO ☐ ☐ ☐

Margin of ulcer

Undermined	(1) Yes	(0) No
Collapsed	(1) Yes	(0) No
Specify, if possible:	(1) flat	(0) edematous
	(8) Red	(2) epithelialization
	(3) Scarring	(4) other:

Moisture balance

Dry	(1) Yes	(0) No
Moist	(1) Yes	(0) No

Wound base

Cotton wool appearance	(1) Yes	(0) No
Granulation tissue	(1) Yes	(0) No
Fibrinous	(1) Yes	(0) No
Necrotic	(1) Yes	(0) No
Odor	(1) Yes	(0) No
Discharge	(1) clear	(0) bloody
	(8) Pus	(2) others:

Surrounding skin

Induration	(1) Yes	(0) No
	Size	
Discoloration	(1) Yes	(0) No
	Size	
Oedema	(1) Yes	(0) No
	Size	
Redness	(1) Yes	(0) No
ΔT	(1) Yes	(0) No

Photographs

Photographs taken (1) Yes (0) No

if no, please specify reason____

if yes, fill in the nos:

Picture nos: _____, _____, _____, _____, _____

Investigator(s) signature(s):
