# Supplementary material for: Assessing and managing wounds of Buruli ulcer patients at the primary and secondary health care levels in Ghana
Source: PLoS Negl Trop Dis. 2017 Feb 28;11(2):e0005331. doi: 10.1371/journal.pntd.0005331 (PMC5345880; doi:10.1371/journal.pntd.0005331)
Supplement: S2 Case Report — (PDF) [file pntd.0005331.s004.pdf]

BU-WOUND-STUDY	FOLLOW-UP	Side Code ☐☐☐	Patient´s NO ☐☐☐

Date: __. __. __ (dd/mm/yy)

General Information

Hospital status	(1) hospitalized	(0) outpatient	(8) discharge<br>date: __.__.__<br>(dd/mm/yy)

Body temperature (axillary): __ __, _ °C

Weight __ __ __ kg

Main treatment

☐ Antimycobacteria agents: ______________________________

☐ Physiotherapy/ physical rehabilitation

☐ Others: ____

Present complaints and since last examination

Pain	(1) Yes*	(0) No

* If yes, specify in the visual analog scale (VAS)

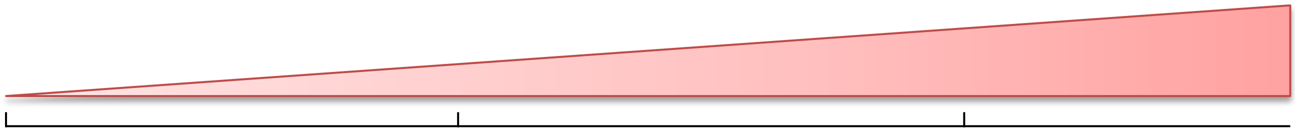

mild pain (1) moderate pain (2) severe pain (8)

Is there an analgesic therapy?	(1) Yes	(0) No
	If yes, please specify:	
Other complaints?	(1) Yes	(0) No
	If yes, please specify:	

Lymph nodes

Palpable	(1) Yes	(0) No
If yes:	(1) generalized	(2) head/ neck
	(3) armpit	(4) grain
Painful on palpation	(1) Yes	(0) No
Skin above palpable lymph node warmer compared to surrounding skin	(1) Yes	(0) No

BU-WOUND-STUDY	FOLLOW-UP	Side Code ☐☐☐	Patient´s NO ☐☐☐

Lesion specific

Fill in one form per lesion in patients with multiple lesions. Number the lesions as in the CRF “Day 0”.

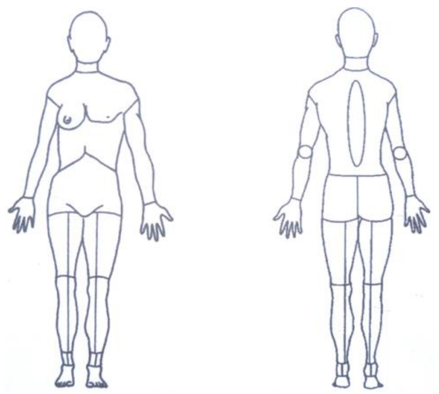

☐ Ulcer ☐ Nodule

Lesion Localization ☐☐☐

Main treatment

- ☐ Dressing
- ☐ Excision
- ☐ Skin graft: Date: __. __. __ (dd/mm/yy)
- ☐ Amputation : Date: __. __. __ (dd/mm/yy)
- ☐ Debridement: Date: __. __. __ (dd/mm/yy)
- ☐ Others: ____

Wound Assessment

Size

Max. diameter: __ __ __ mm   Area of the wound __ __ __ mm²

Max. diameter: __ __ __ mm   Area of the induration __ __ __ mm²

Epithelialization	(1)	Yes	(0)	No
Eschar	(1)	Yes	(0)	No
Exudation	(1)	Yes	(0)	No
Scarring	(1)	Yes	(0)	No
Moisture balance	(1)	dry	(0)	moisture
Wound base	(1)	clear discharge	(0)	necrotic
	(8)	white- cotton wool	(2)	fibrinous
Granulation tissue	(1)	Yes	(0)	No
If yes, specify:	(1)	normal	(0)	discoloration
	(8)	other:		
Margin of the ulcer	(1)	undermined	(0)	collapsed
	(8)	flat	(0)	edematous
	(2)	reddened		

BU-WOUND-STUDY	FOLLOW-UP	Side Code ☐☐☐	Patient´s NO ☐☐☐

Surrounding skin of the lesion	(0) induration, size:	(1) oedema, size
	(8) discoloration, size:	(2) ΔT
	(3) redness	

Microbiological assessment

Wound surface looks	(1) opaque	(0) yellow
	(8) green	
Discharge	(1) Yes	(0) No
If yes please specify:	(1) clear	(0) bloody
	(8) pus	(2) others:
Odor	(1) Yes	(0) No

Laboratory

Swab, Microbiology	(1) done	(0) not done	(8) not applicable
Blood sample	(1) done	(0) not done	(8) not applicable
Blood culture	(1) done	(0) not done	(8) not applicable
Punch biopsy	(1) done	(0) not done	(8) not applicable
Tissue samples (Debridement/ Excision)	(1) done	(0) not done	(8) not applicable

Photographs

Photographs taken (1) Yes (0) No

if no, please specify reason____

if yes, fill in the nos:

Picture nos: _____, _____, _____, _____, _____

Investigator(s) signature(s):
