# Supplementary material for: Assessing and managing wounds of Buruli ulcer patients at the primary and secondary health care levels in Ghana
Source: PLoS Negl Trop Dis. 2017 Feb 28;11(2):e0005331. doi: 10.1371/journal.pntd.0005331 (PMC5345880; doi:10.1371/journal.pntd.0005331)
Supplement: S3 Case Report — (PDF) [file pntd.0005331.s005.pdf]

Case Report OBOM

Acute Buruli Ulcer Wounds

Patient No. 001

1. Demographic data

Sex: Male
Age: 44 years

2. Wound description

BU Category I (see Photo documentation)

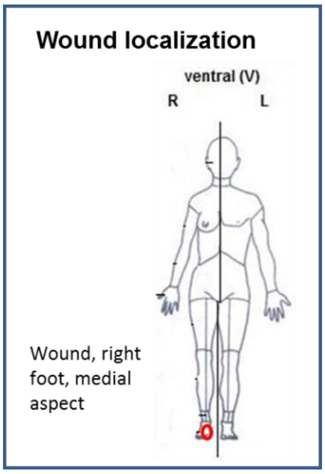

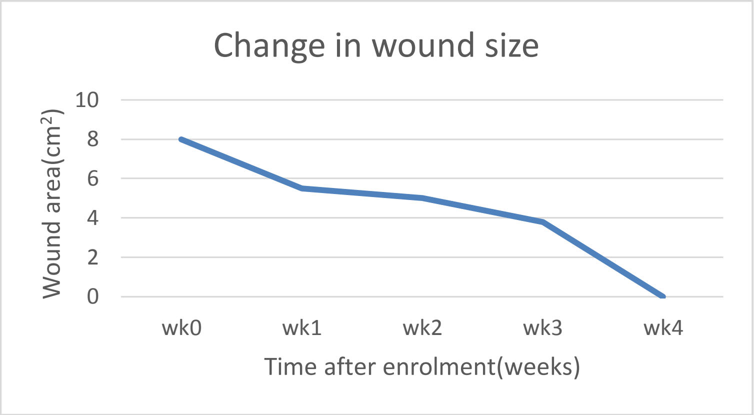

3. Medical History

Nil of significance

Wound History:

Wound observed since: 08/2013

BU- Treatment: 25/09/13- 20/11/13

Wound healed: 15/11/13

4. Physical examination

Body- Mass- index (BMI) 21.41 kg KG/m²

All systems normal

5. Current Medication

Streptomycin 1000mg, Rifampicin 600mg. 56 doses taken

Patient had been on BU treatment for 14 days before enrolment

Paracetamol (occasionally)

6. Laboratory

BU confirmation: ZN (-) PCR (+) for *M.ulcerans*

Retroscreen: non reactive

7. Photo documentation

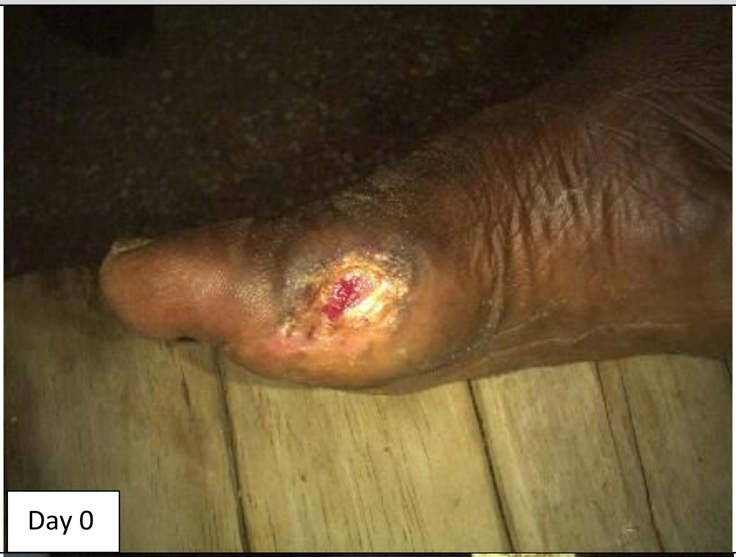

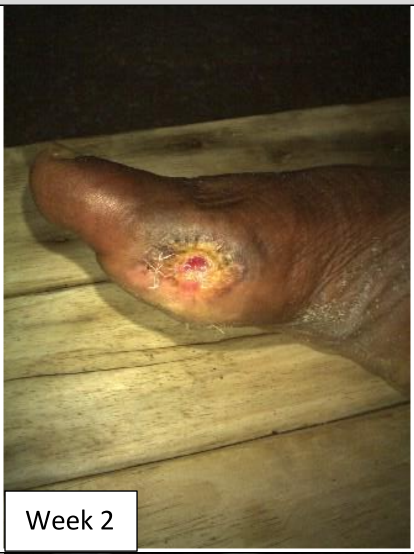

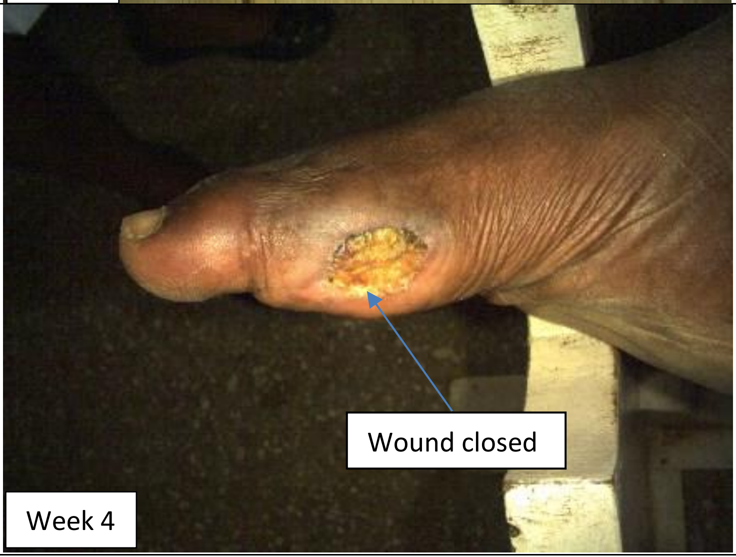

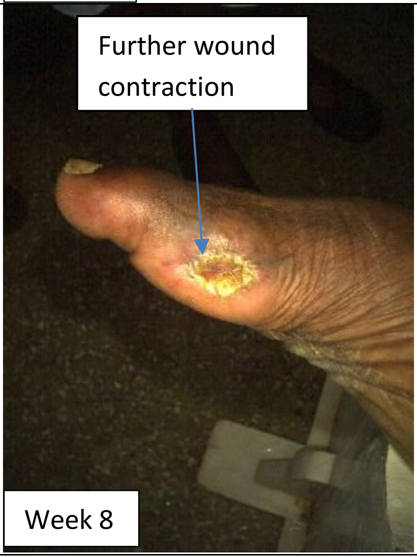

Case Report OBOM

Acute Buruli Ulcer Wounds

Patient No. 002

1. Demographic data

Sex: Male
Age: 29 years

2. Wound description

BU Category I (see Photo documentation)

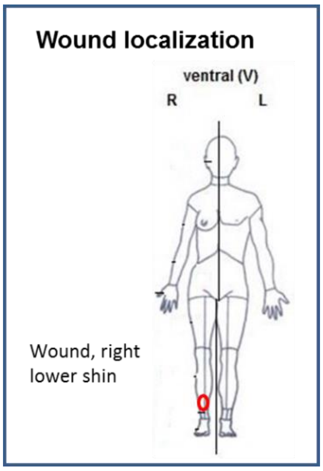

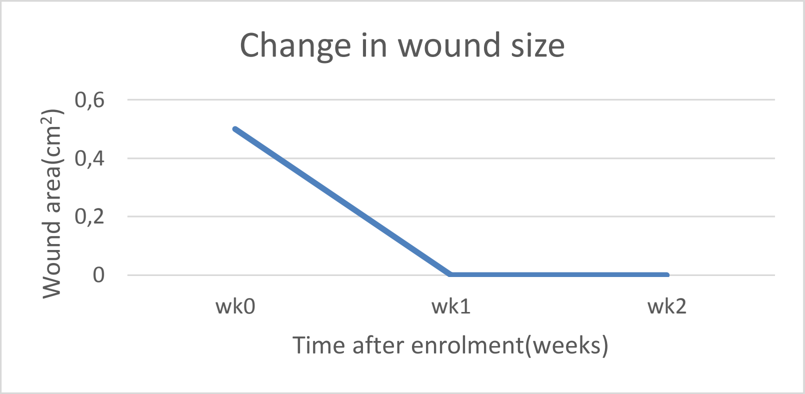

3. Medical History

Nil of significance

<u>Wound History:</u>

Wound observed since: 08/2013

BU- Treatment: 21/08/13- 15/10/13

Wound healed: 15/10/13

4. Physical examination

Body- Mass- index (BMI) 21.03 kg KG/m²

All systems normal

5. Current Medication

Streptomycin 1000mg, Rifampicin 600mg. 56 doses taken

Patient had been on BU treatment for 49 days before enrolment

6. Laboratory

BU confirmation: PCR (+) ZN (-) for *M. ulcerans*

Retroscreen: Non reactive

7. Photo documentation

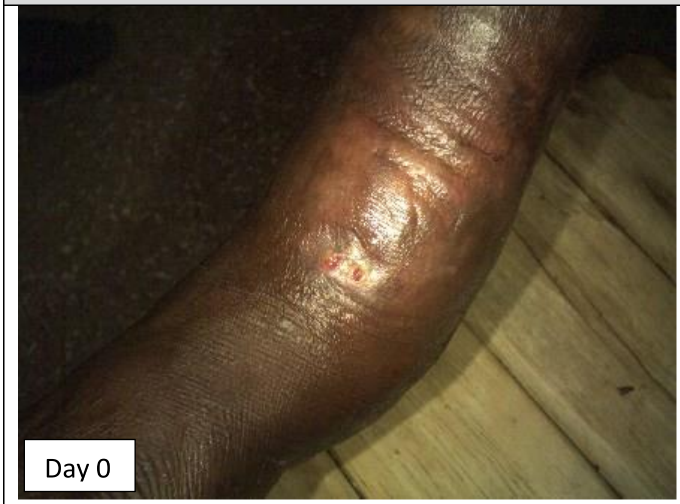

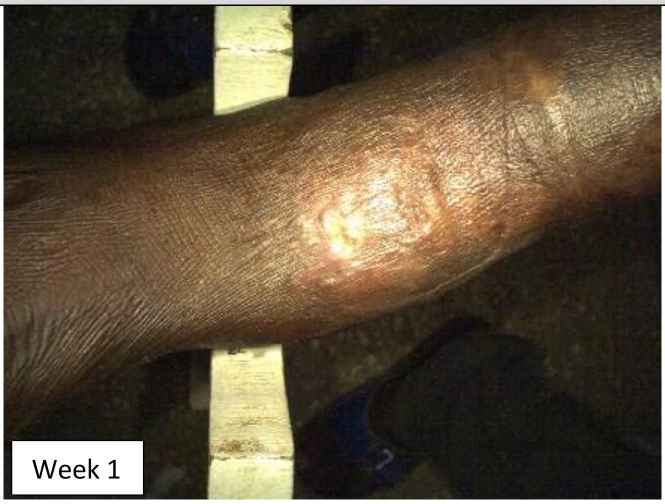

Case Report OBOM

Acute Buruli Ulcer Wounds

Patient No. 003

1. Demographic data

Sex: Male
Age: 9 years

2. Wound description

BU Category I (see Photo documentation)

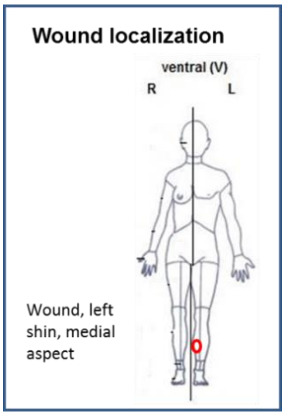

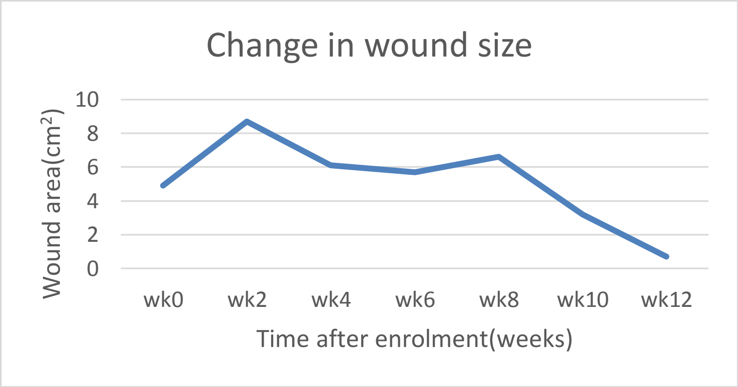

3. Medical History

Nil of significance

<u>Wound History:</u>

Wound observed since: 08/2013

BU- Treatment: 26/09/13- 28/11/13

Wound healed: 12/04/14

4. Physical examination

Body- Mass- index (BMI) 19.7 kg KG/m^2

All systems normal

5. Current Medication

Streptomycin 750mg, Rifampicin 450mg. 56 doses taken

Patient had been on BU treatment for 20 days before enrolment

6. Laboratory

BU confirmation: PCR (+) ZN (+) for *M. ulcerans*

Retroscreen: Non reactive

7. Photo documentation

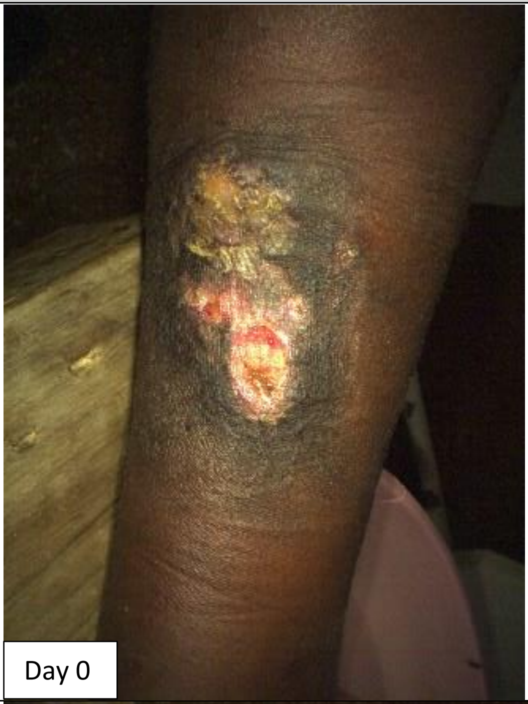

Day 0

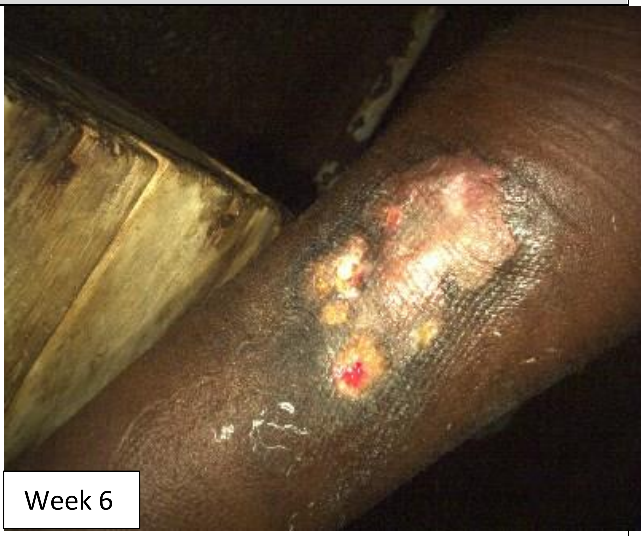

Week 6

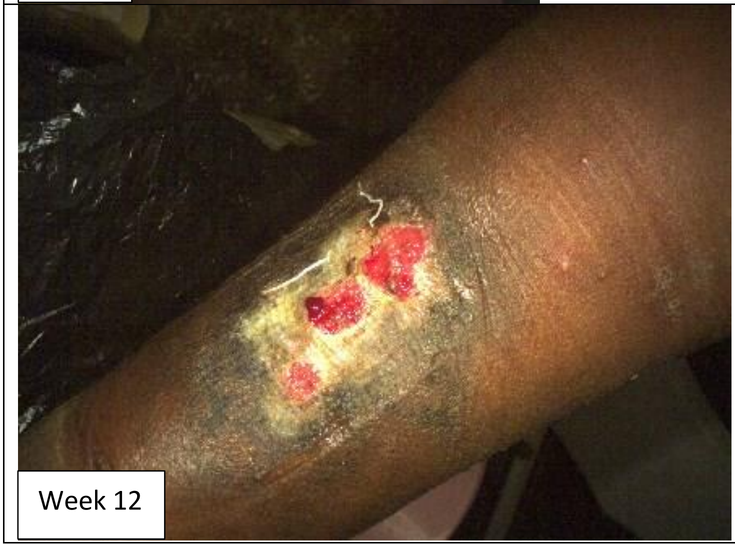

Week 12

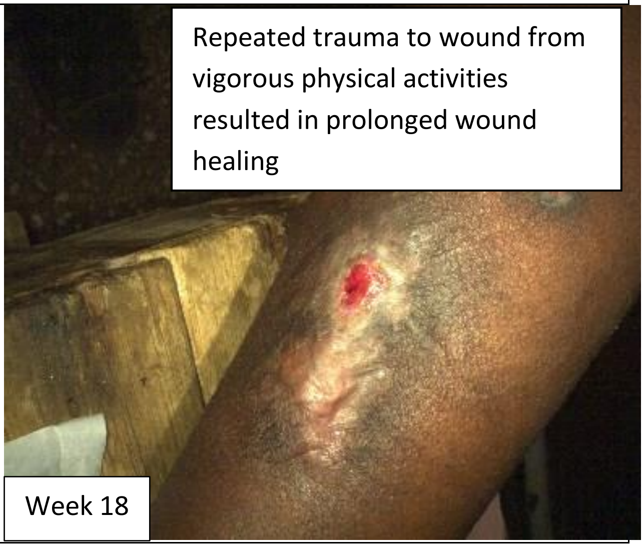

Repeated trauma to wound from vigorous physical activities resulted in prolonged wound healing

Week 18

Case Report OBOM

Acute Buruli Ulcer Wounds

Patient No. 004

1. Demographic data

Sex: Female
Age: 4 years

2. Wound description

BU Category I (see Photo documentation)

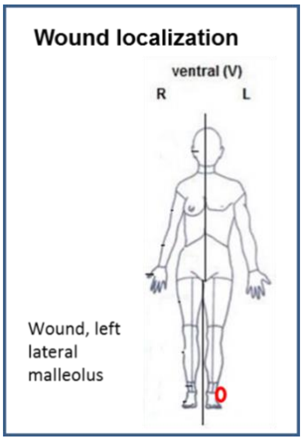

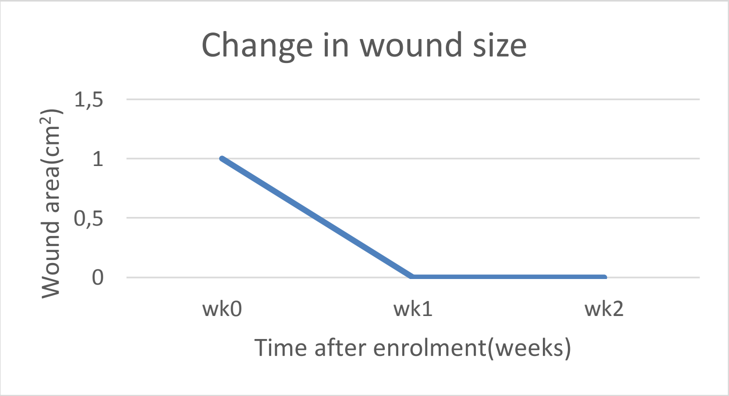

3. Medical History

Nil of significance

<u>Wound History:</u>

Wound observed since: 04/2013

BU- Treatment: 08/10/13- 05/12/13

Wound healed: 30/10/13

4. Physical examination

Body- Mass- index (BMI) 15.0 kg KG/m²

All systems normal

5. Current Medication

Streptomycin 330mg, Rifampicin 150mg. 56 doses taken

Patient had been on BU treatment for 8 days before enrolment

6. Laboratory

BU confirmation: PCR (+) ZN (-) for *M. ulcerans*

Retroscreen: Non reactive

7. Photo documentation

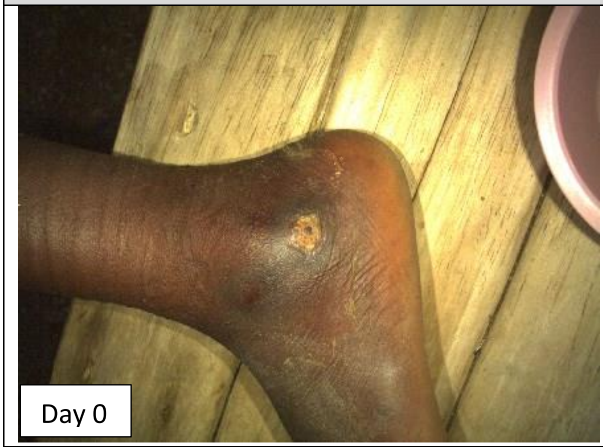

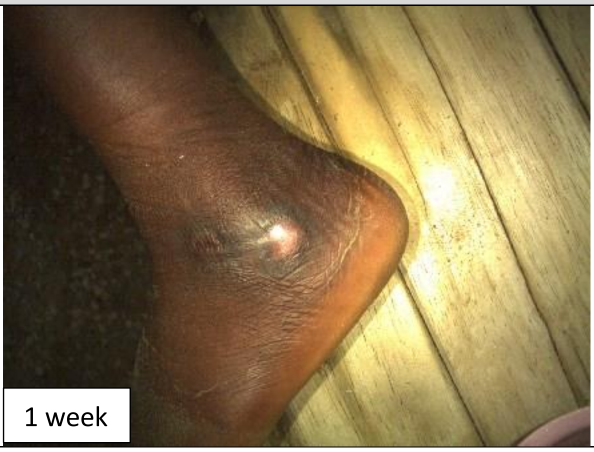

Case Report OBOM

Acute Buruli Ulcer Wounds

Patient No. 008

1. Demographic data

Sex: Male
Age: 40 years

2. Wound description

BU Category III (see Photo documentation)

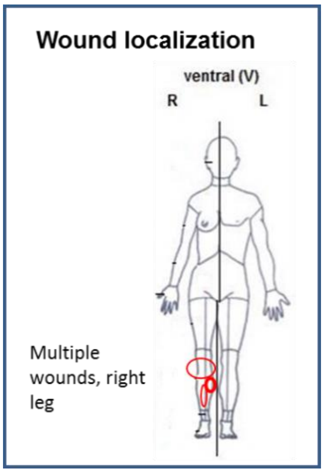

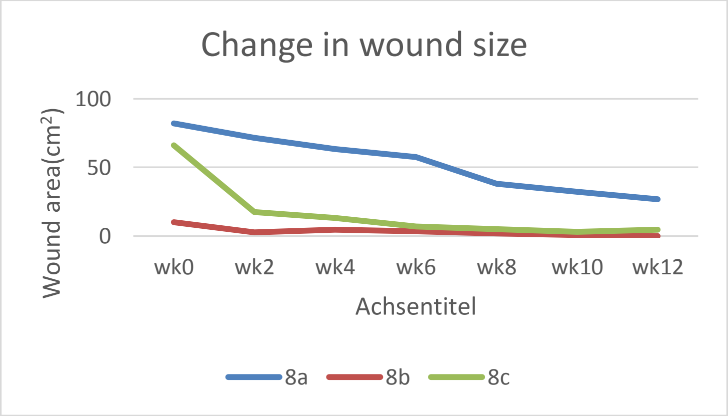

3. Medical History

Nil of significance

<u>Wound History:</u>

Wound observed since: 09/2013

BU- Treatment: 30/10/13- 26/12/13

Last wound healed: 04/2014

4. Physical examination

Body- Mass- index (BMI) 18.73 kg KG/m²

All systems normal

5. Current Medication

Streptomycin 1000mg, Rifampicin 600mg. 56 doses taken

6. Laboratory

BU confirmation: PCR (+) ZN (-) for *M. ulcerans*

Retroscreen: Non reactive

7. Photo documentation

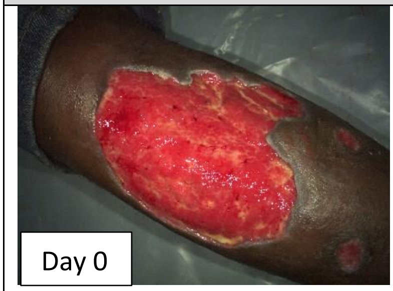 Day 0	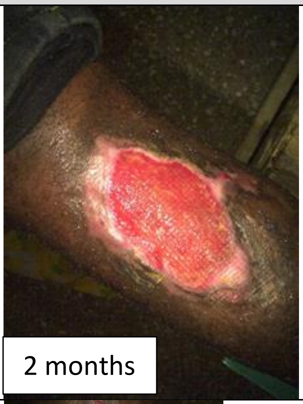 2 months	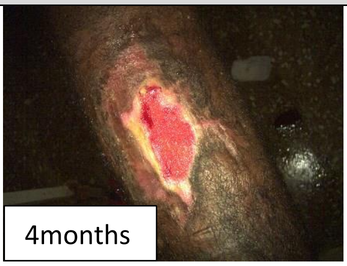 4months	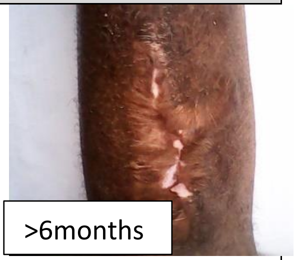 >6months
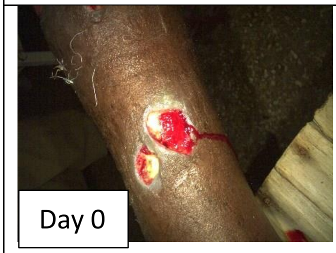 Day 0	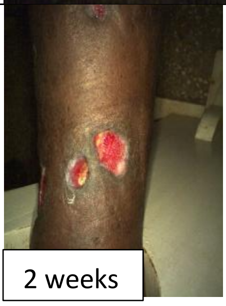 2 weeks	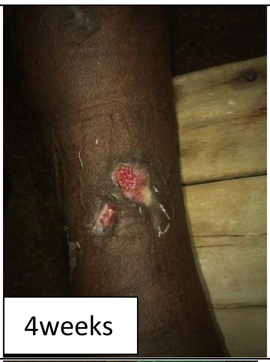 4weeks	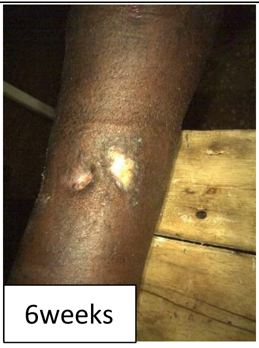 6weeks
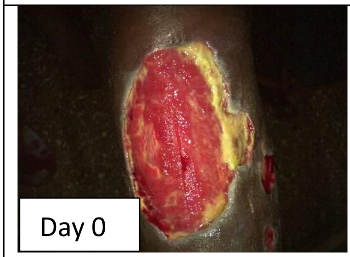 Day 0	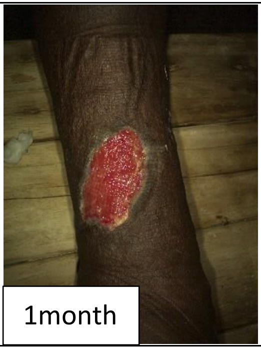 1month	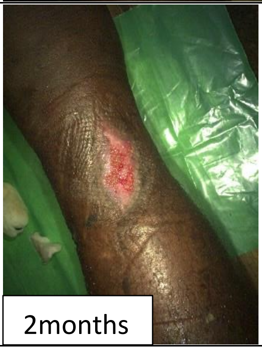 2months	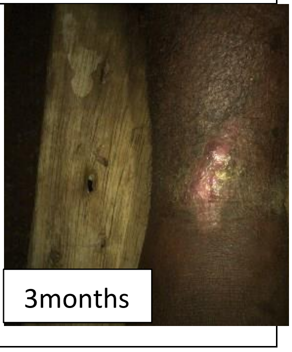 3months

Case Report OBOM

Acute Buruli Ulcer Wounds

Patient No. 010

1. Demographic data

Sex: Male
Age: 10 years

2. Wound description

BU Category III (see Photo documentation)

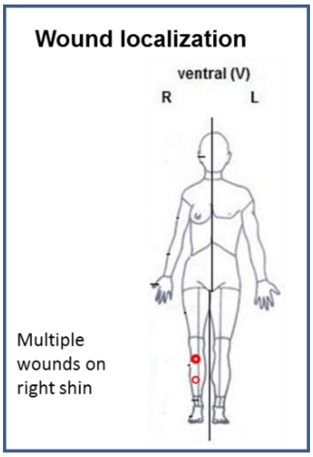

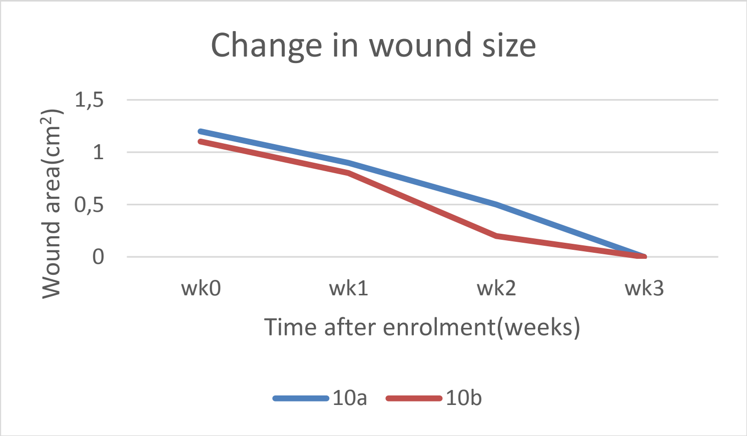

3. Medical History

Nil of significance

<u>Wound History:</u>

Wound observed since: 05/2013

BU- Treatment: 13/11/13- 20/01/14

Wound healed: 11/12/13

4. Physical examination

Body- Mass- index (BMI) 17.86 kg KG/m²

All systems normal

5. Current Medication

Streptomycin 750mg, Rifampicin 450mg 56 doses taken

6. Laboratory

BU confirmation: PCR (+) ZN (-) for *M. ulcerans*

Retroscreen: Non reactive

7. Photo documentation
Wound 10a

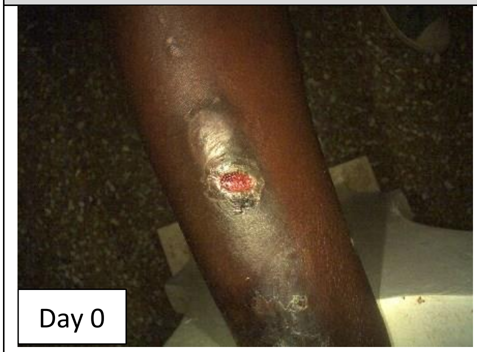

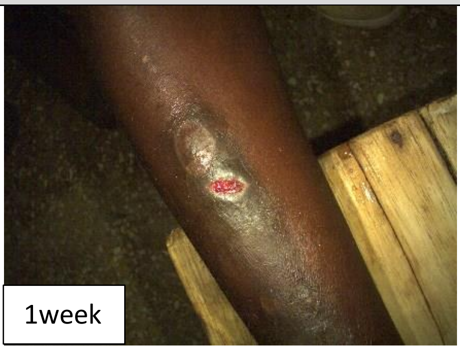

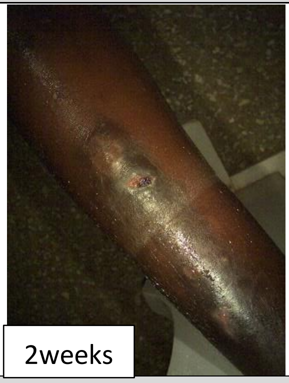

Photo documentation:
Wound 10b

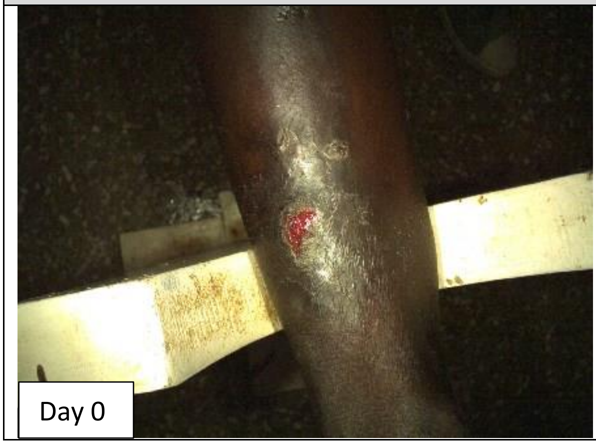

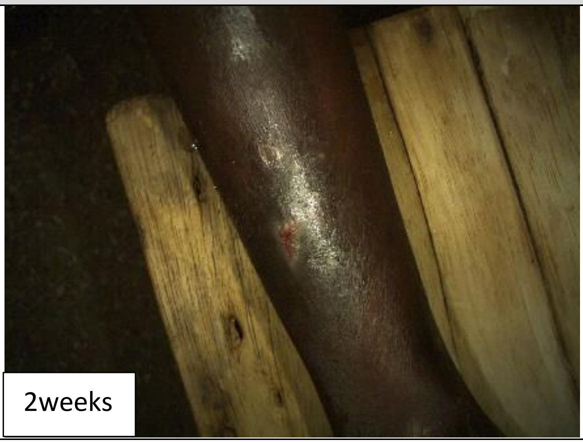

Case Report OBOM

Acute Buruli Ulcer Wounds

Patient No. 021

1. Demographic data

Sex: Male
Age: 23 years

2. Wound description

BU Category III (see Photo documentation)

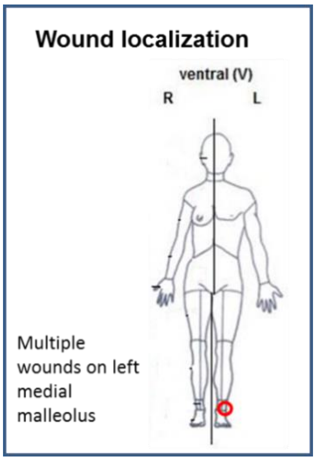

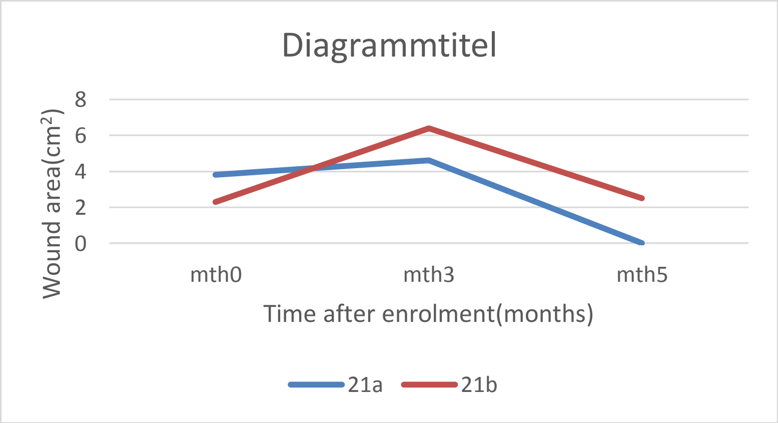

3. Medical History

Sickle Cell Disease

<u>Wound History:</u>

Wound observed since: 12/2013

BU- Treatment: 21/05/14- 29/07/14

Wound healed: 11/2014

4. Physical examination

Body- Mass- index (BMI) 20.69 kg KG/m²

All systems normal

5. Current Medication

Streptomycin 1000mg, Rifampicin 600mg. 56 doses taken

Haematinics

6. Laboratory

BU confirmation: PCR (+) ZN (-) for *M. ulcerans*

Retroscreen: Non reactive

Hb electrophoresis: SS genotype

7. Photo documentation:

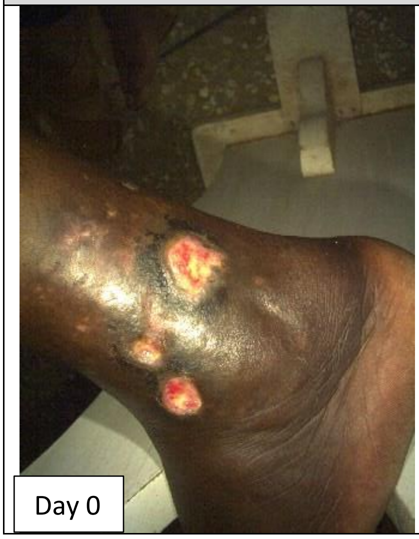

Day 0

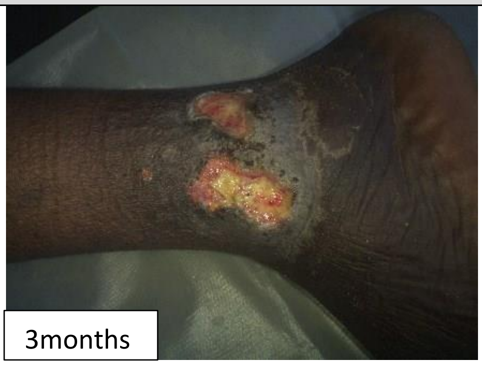

3months

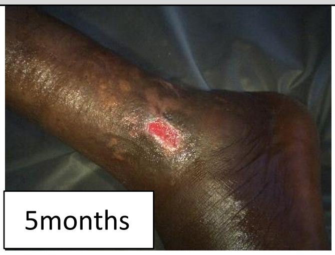

5months

Case Report OBOM

Acute Buruli Ulcer Wounds

Patient No. 022

1. Demographic data

Sex: Male
Age: 57 years

2. Wound description

BU Category III (see Photo documentation)

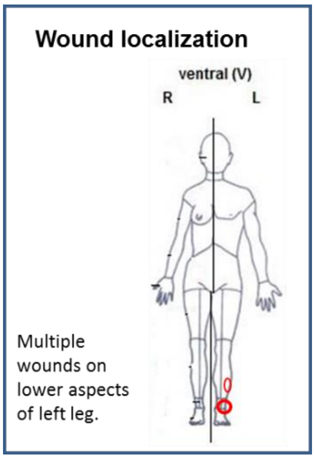

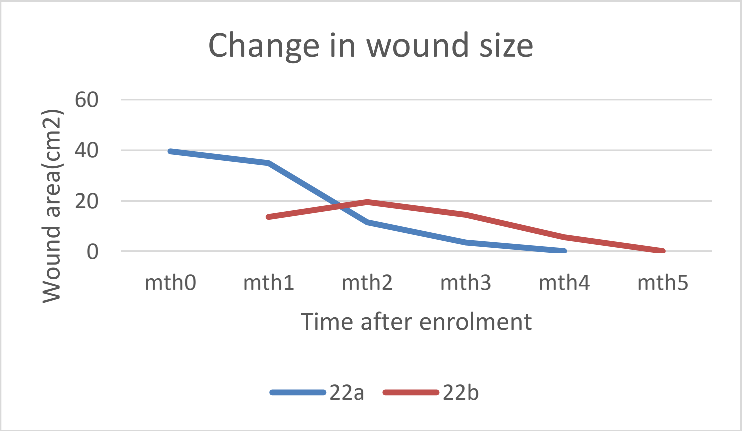

3. Medical History

Hypertensive

<u>Wound History:</u>

Wound observed since: 03/2014

BU- Treatment: 04/06/14- 03/08/14

All wounds healed by: 12/11/2014

4. Physical examination

Body- Mass- index (BMI) 24.93 kg KG/m²

All systems normal

5. Current Medication

Streptomycin 1000mg, Rifampicin 600mg. 56 doses taken

6. Laboratory

BU confirmation: PCR (+) ZN (-) for *M. ulcerans*

Retroscreen: Non reactive

7. Photo documentation: Wound 22a

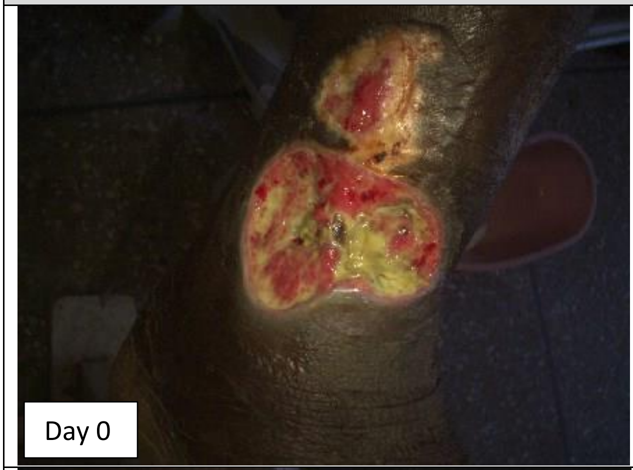

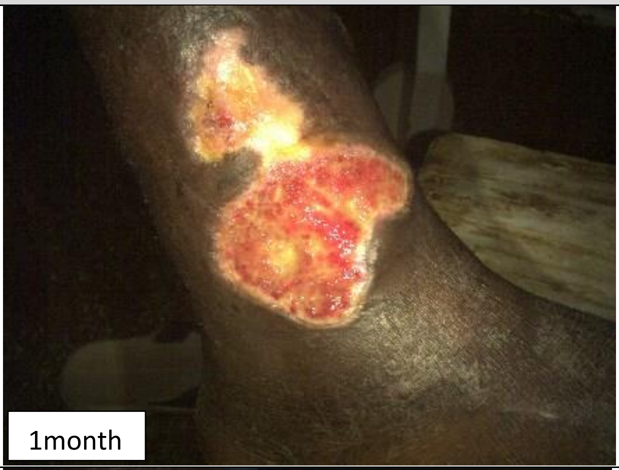

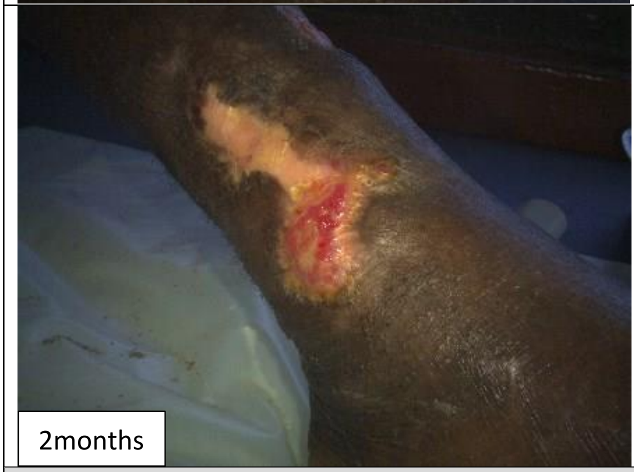

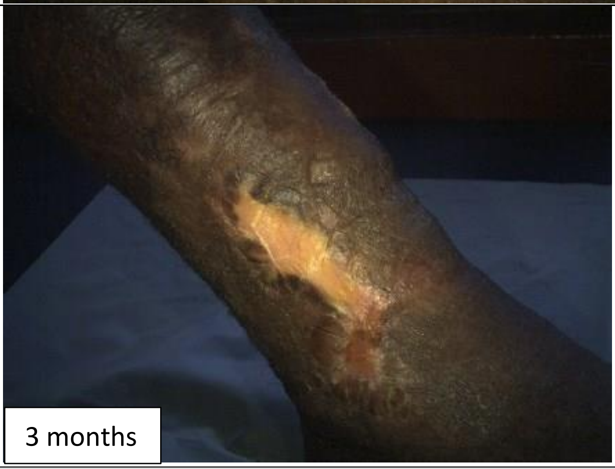

Wound 22b

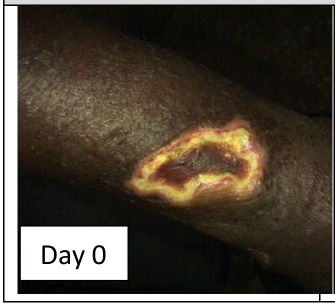

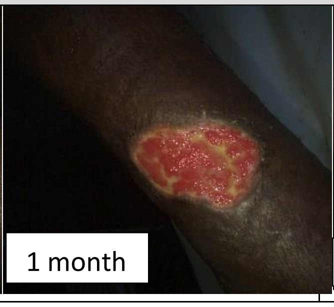

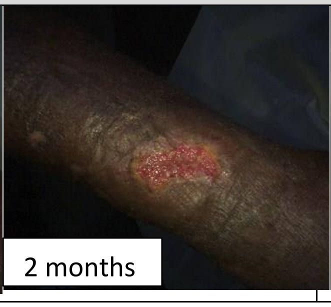

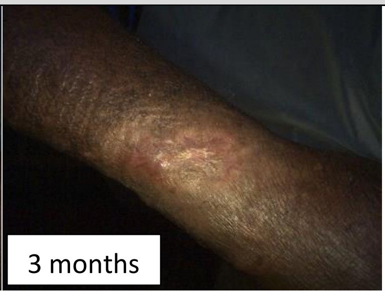

Case Report OBOM

Acute Buruli Ulcer Wounds

Patient No. 023

1. Demographic data

Sex: Male
Age: 2.5 years

2. Wound description

BU Category I (see Photo documentation)

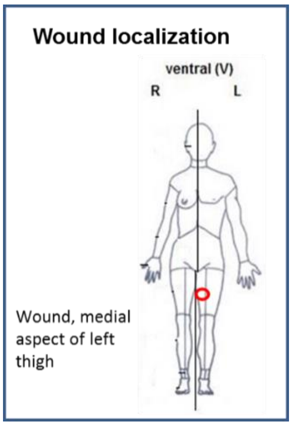

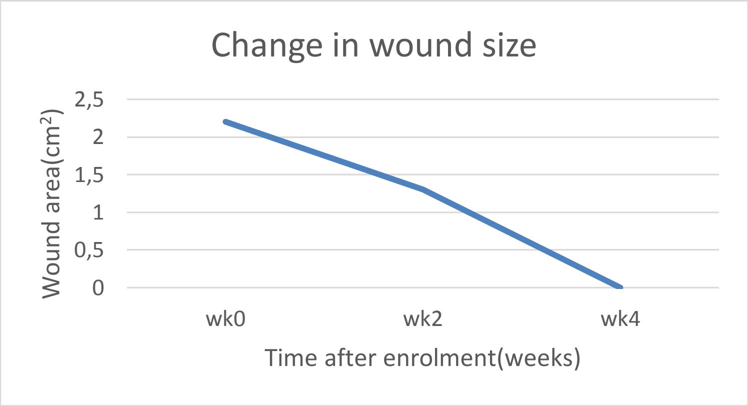

3. Medical History

Nil of significance

Wound History:

Wound observed since: 04/2014

BU- Treatment: 02/07/14- 05/09/14

Wound healed by: 04/08/2014

4. Physical examination

Body- Mass- index (BMI) 15.95 kg KG/m²

All systems normal

5. Current Medication

Streptomycin 330mg, Rifampicin 150mg. 56 doses taken

6. Laboratory

BU confirmation: PCR (+) ZN (+) for *M. ulcerans*

Retroscreen: Non reactive

7. Photo documentation:

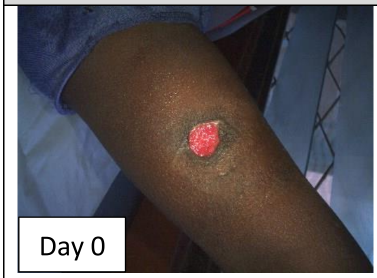

Day 0

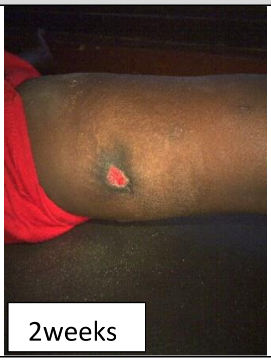

2weeks

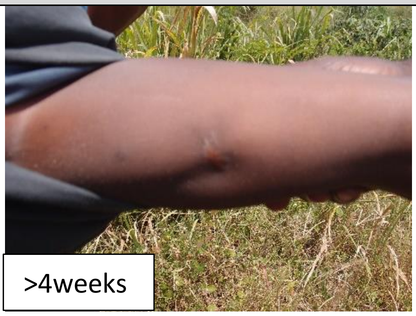

>4weeks

Case Report OBOM

Acute Buruli Ulcer Wounds

Patient No. 024

1. Demographic data

Sex: Female
Age: 6 years

2. Wound description

BU Category II (see Photo documentation)

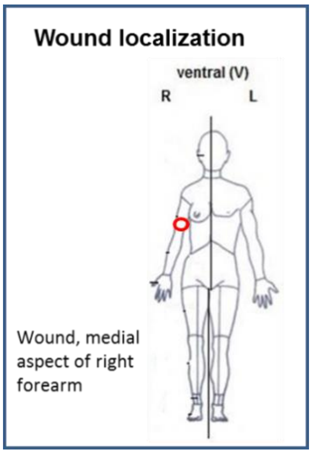

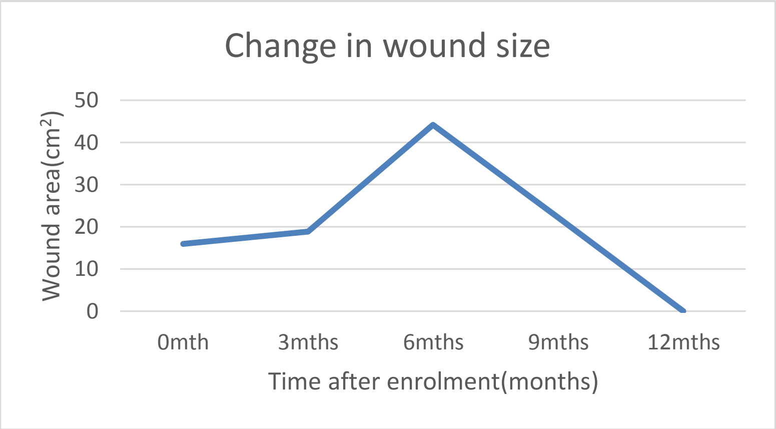

3. Medical History

Nil of significance

<u>Wound History:</u>

Wound observed since: 07/2014

BU- Treatment: 20/08/14- 17/10/14

Wound healed by: 11/2015

4. Physical examination

Body- Mass- index (BMI) 14.24 kg KG/m²

All systems normal

5. Current Medication

Streptomycin 330mg, Rifampicin 150mg. 56 doses taken

Patient had been on BU treatment for 7 days before enrolment

6. Laboratory

BU confirmation: PCR (+) ZN (-) for *M. ulcerans*

Retroscreen: Non reactive

7. Photo documentation:

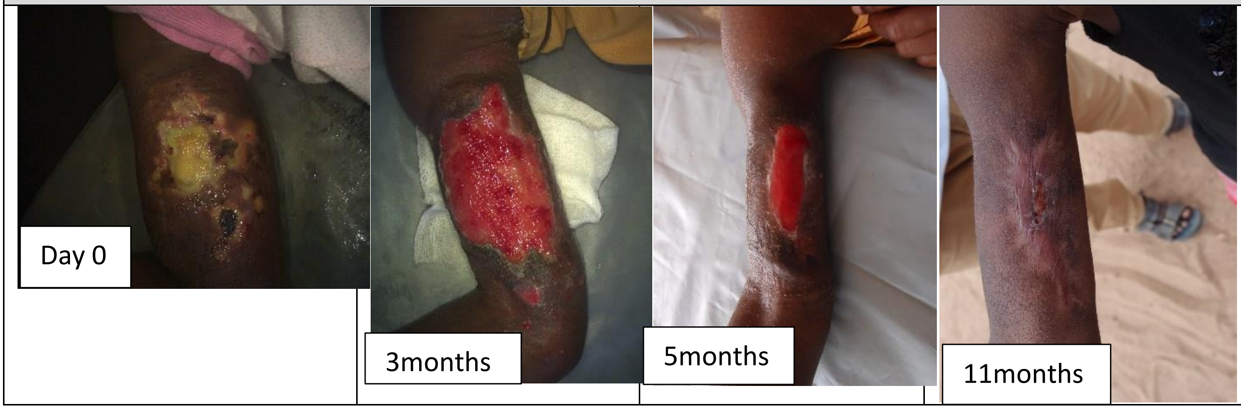

Case Report OBOM

Acute Buruli Ulcer Wounds

Patient No. 025

1. Demographic data

Sex: Female
Age: 42 years

2. Wound description

BU Category I (see Photo documentation)

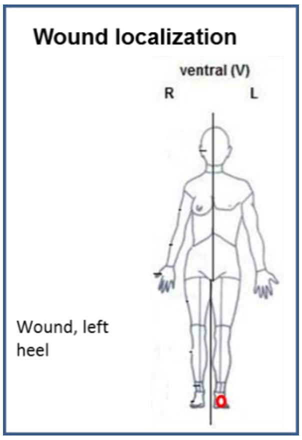

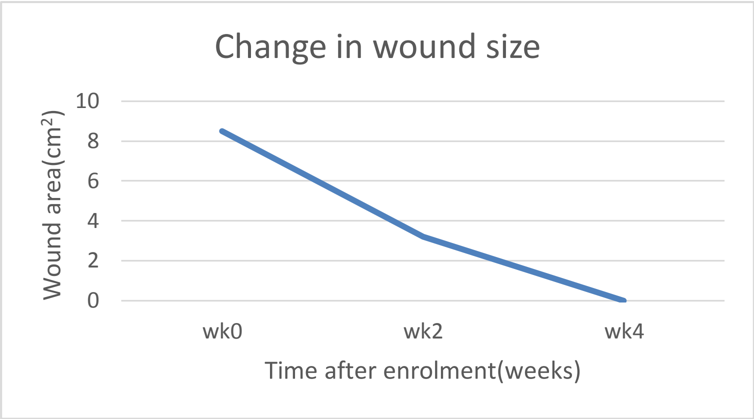

3. Medical History

Nil of significance

<u>Wound History:</u>

Wound observed since: 10/2014

BU- Treatment: 05/11/14- 17/12/14

Wound healed by: 10/12/14

Patient was lost to follow up after 4 weeks of treatment

4. Physical examination

Body- Mass- index (BMI) 22.9 kg KG/m²

All systems normal

5. Current Medication

Streptomycin 1000mg, Rifampicin 600mg. 26 doses taken

Patient had been on BU treatment for 7 days before enrolment.

6. Laboratory

BU confirmation: PCR (+) ZN (-) for *M. ulcerans*

Retroscreen: Non reactive

7. Photo documentation:

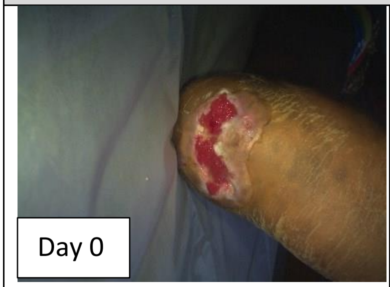

Day 0

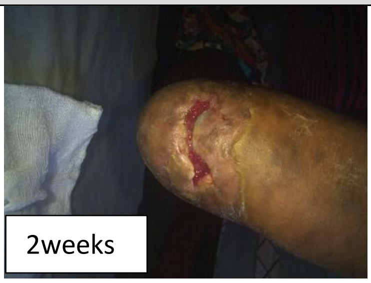

2weeks

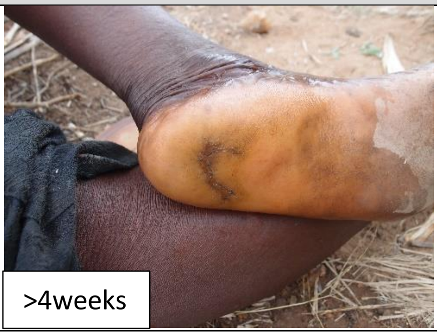

>4weeks

Case Report OBOM

Acute Buruli Ulcer Wounds

Patient No. 026

1. Demographic data

Sex: Male
Age: 36 years

2. Wound description

BU Category III (see Photo documentation)

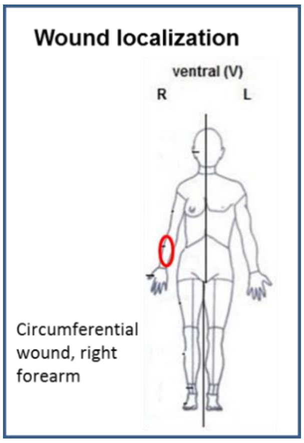

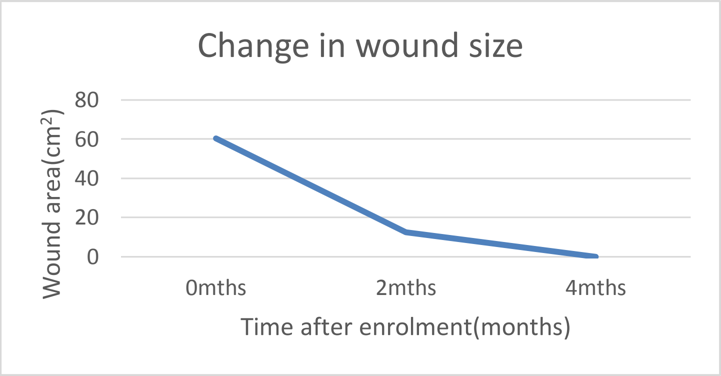

3. Medical History

Nil of significance

Wound History:

Had a previous large wound at same site treated with herbal medication 25 years before new wound
New wound observed since: 09/2014

BU- Treatment: 05/11/14- 02/01/15

Wound healed by: 03/2015

.

4. Physical examination

Body- Mass- index (BMI) 22.5 kg KG/m²

All systems normal

5. Current Medication

Streptomycin 1000mg, Rifampicin 600mg. 56 doses taken

Patient had been on BU treatment for 7 days before enrolment.

Paracetamol

6. Laboratory

BU confirmation: PCR (+) ZN (-) for *M. ulcerans*

Retroscreen: Non reactive

7. Photo documentation:

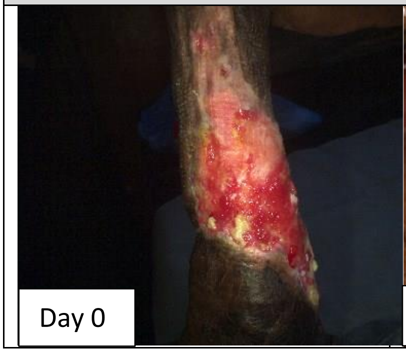

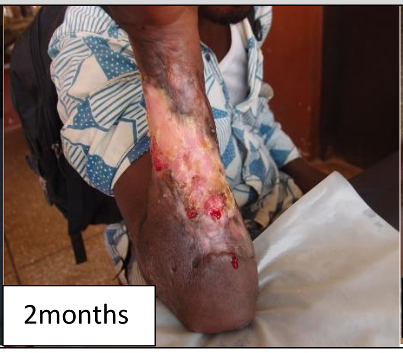

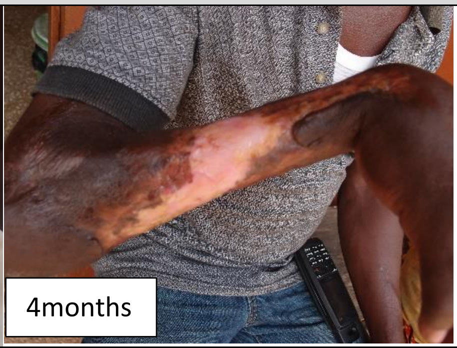

Case Report OBOM

Acute Buruli Ulcer Wounds
Patient No. 027

1. Demographic data

Sex: Female
Age: 21 years

2. Wound description

BU Category II (see Photo documentation)

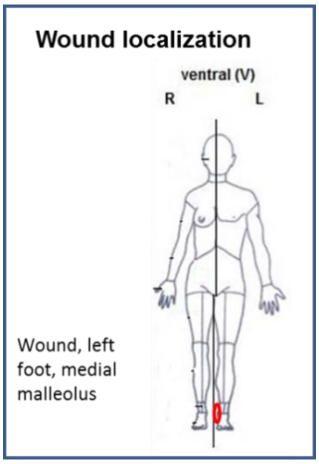

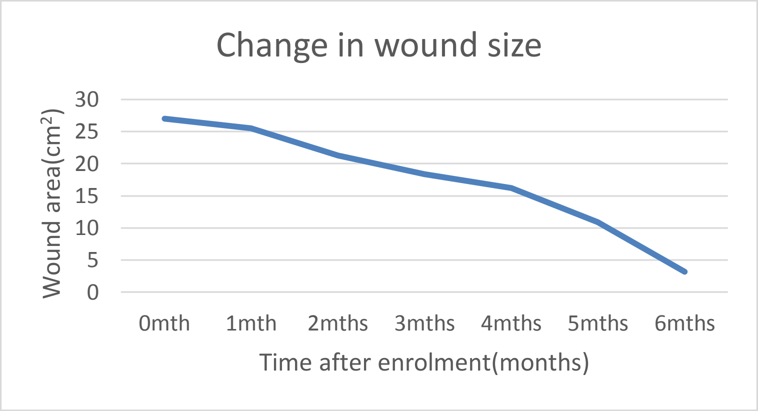

3. Medical History

Nil of significance

<u>Wound History:</u>

Wound observed since: 11/2013

BU- Treatment: 21/11/14- 26/01/15

Almost healed

4. Physical examination

Body- Mass- index (BMI) 21.5 kg KG/m²

All systems normal

5. Current Medication

Streptomycin 1000mg, Rifampicin 600mg. 56 doses taken

Patient had been on BU treatment for 5 days before enrolment.

Paracetamol

6. Laboratory

BU confirmation: PCR (+) ZN (-) for *M. ulcerans*

Retroscreen: Non reactive

7. Photo documentation:

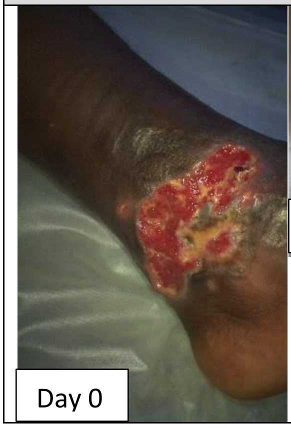

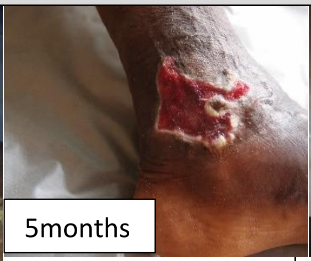

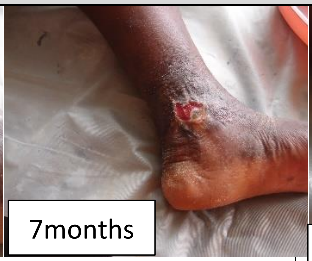

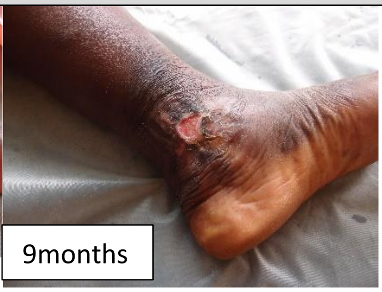

Case Report OBOM

Chronic Buruli Ulcer Wounds

Patient No. 006

1. Demographic data

Sex: Female
Age: 9 years

2. Wound description

BU Category II (see Photo documentation)

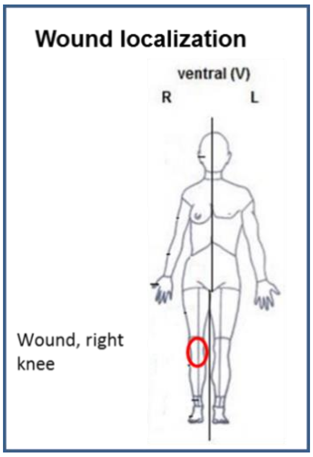

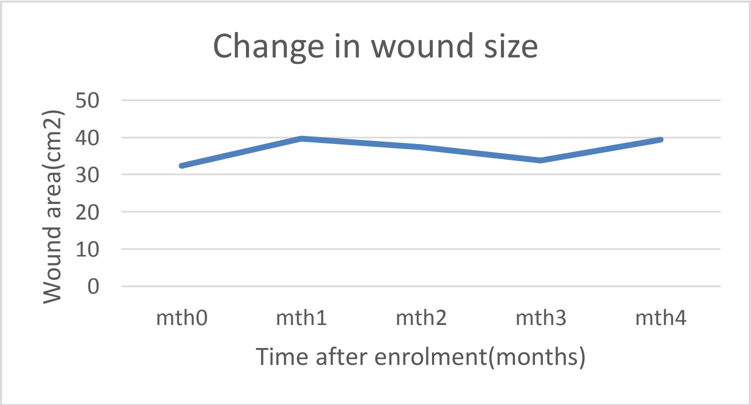

3. Medical History

Nil of significance

<u>Wound History:</u>

Wound observed since: 10/2011

BU- Treatment: 14/08/13- 09/10/13

Patient was lost to follow- up after 4 months of wound monitoring

4. Physical examination

Body- Mass- index (BMI) 14.58 kg KG/m²

All systems normal

5. Current Medication

Streptomycin 500mg, Rifampicin 300mg. 56 doses taken

Patient had completed BU treatment 2 weeks before enrolment..

6. Laboratory

BU confirmation: PCR (+) ZN (-) for *M. ulcerans*

Retroscreen: Non reactive

7. Photo documentation:

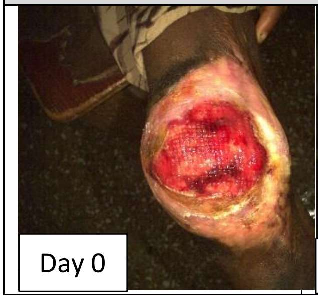

Day 0

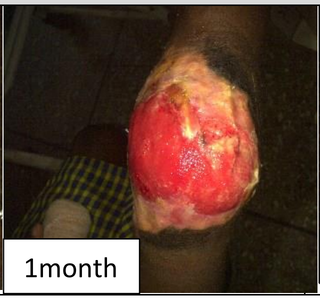

1month

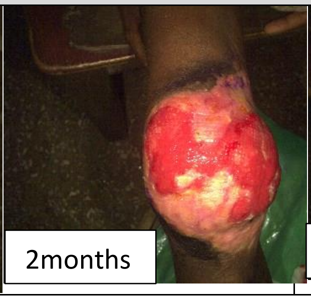

2months

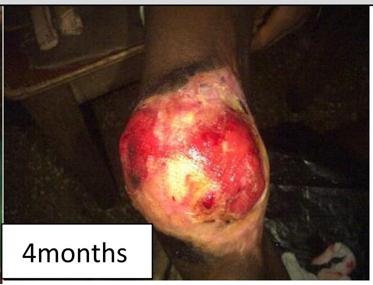

4months

Case Report OBOM

Chronic Buruli Ulcer Wounds

Patient No. 013

1. Demographic data

Sex: Male
Age: 45 years

2. Wound description

BU Category III (see Photo documentation)

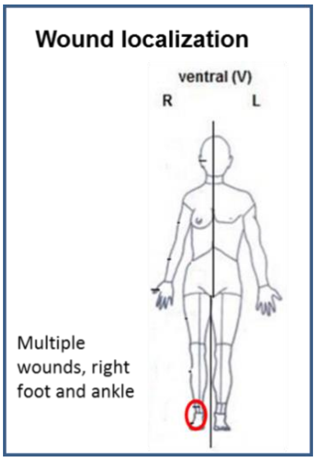

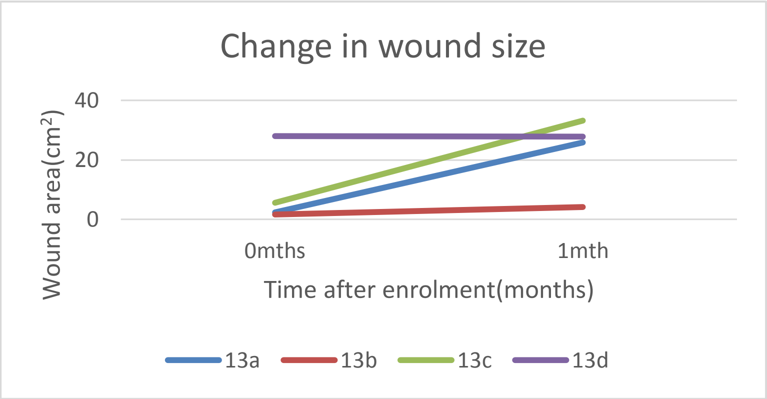

3. Medical History

Retroviral infection

<u>Wound History:</u>

Wound observed since: 10/2011

BU- Treatment: 28/11/13- 24/01/14

Patient was lost to follow up after 1 month of wound monitoring

4. Physical examination

Body- Mass- index (BMI) 19.61 kg KG/m²

All systems normal

5. Current Medication

Streptomycin 1000mg, Rifampicin 600mg. 56 doses taken

Patient had been on BU treatment for 20 days before enrolment.

6. Laboratory

BU confirmation: PCR (+) ZN (+) for *M. ulcerans*

Retroscreen: Reactive

7. Photo documentation:
Wound 13a

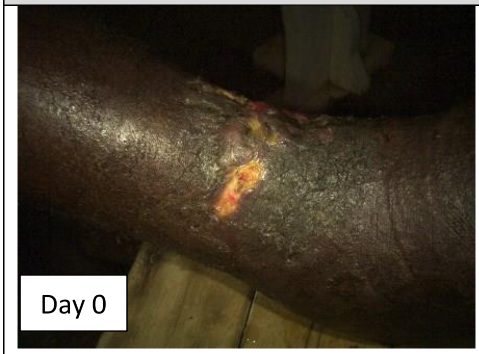

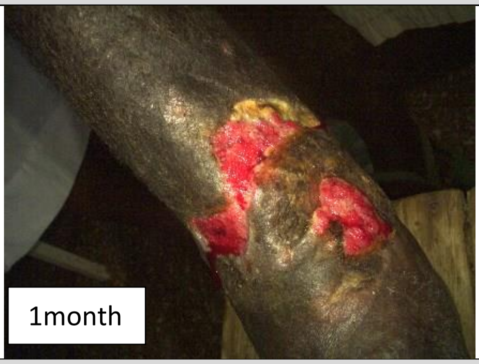

Wound 13b

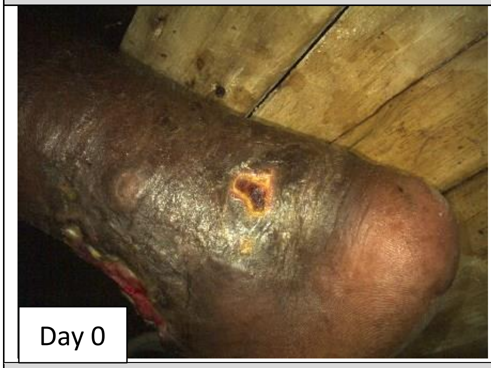

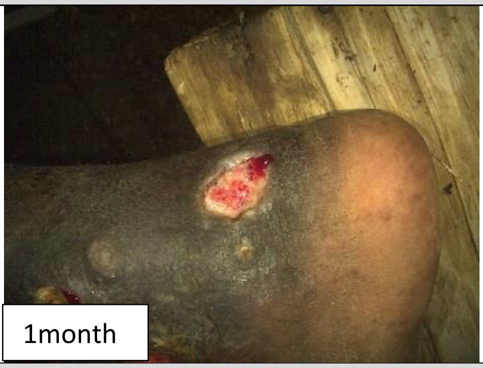

Wound 13c

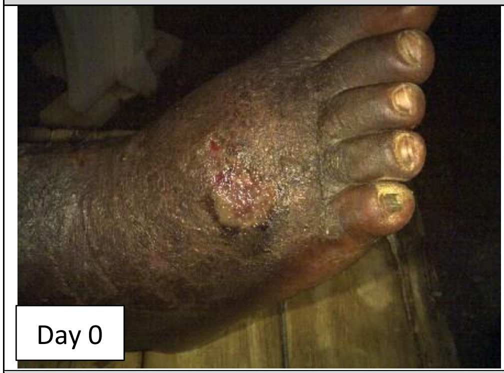

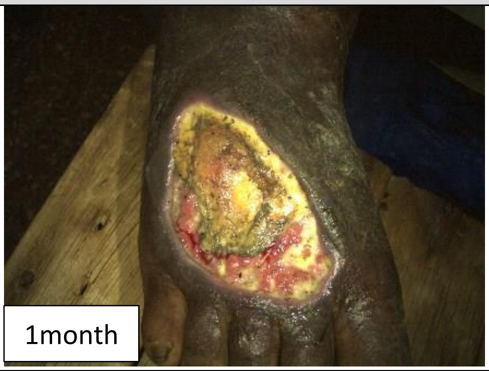

Wound 13d

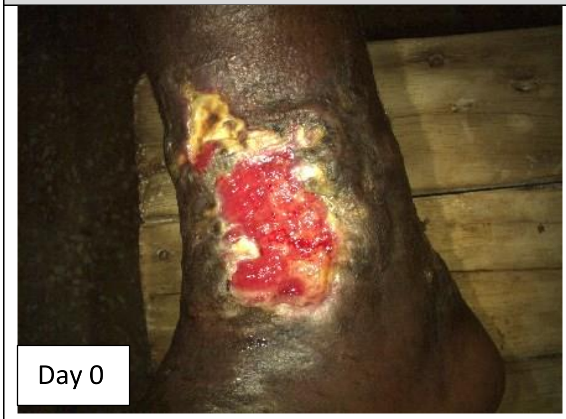

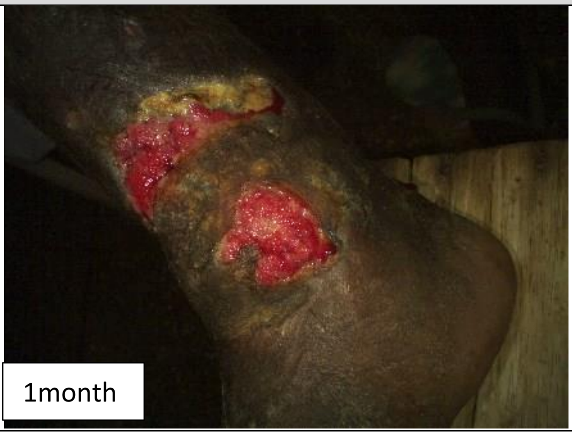

Case Report OBOM

Chronic Buruli Ulcer Wounds
Patient No. 007

1. Demographic data

Sex: Male
Age: 49 years

2. Wound description

BU Category III (see Photo documentation)

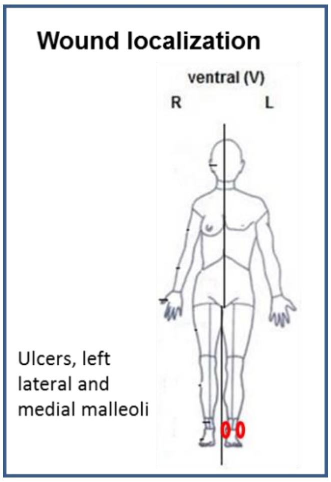

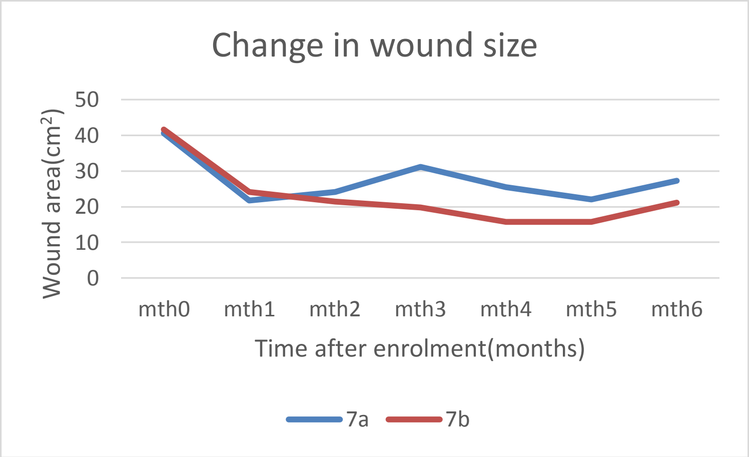

3. Medical History

Nil of significance

<u>Wound History:</u>

Wound observed since: 02/2013

Treatment for previous wounds at same sites:2012

Non healing

Affected limb with signs of chronic lymphoedema

4. Physical examination

Body- Mass- index (BMI) 19.25 kg KG/m²

All systems normal

5. Current Medication

Streptomycin 1000mg, Rifampicin 600mg. 56 doses taken

Patient had completed BU treatment 1 year before enrolment.

6. Laboratory

BU confirmation: PCR (+) ZN (-) for *M. ulcerans*

Retroscreen: Non reactive

7. Photo documentation:

Wound 7a

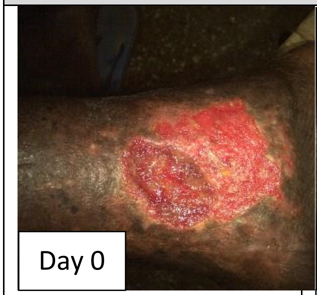

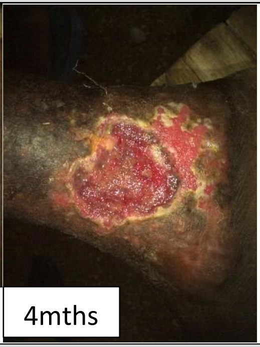

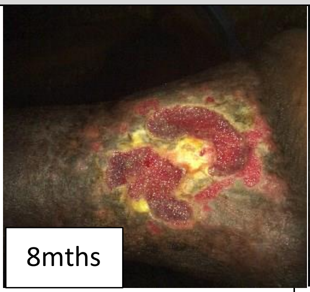

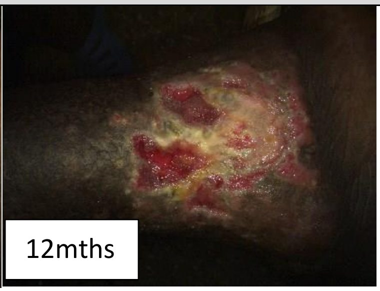

Wound 7b

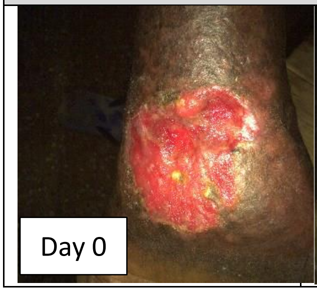

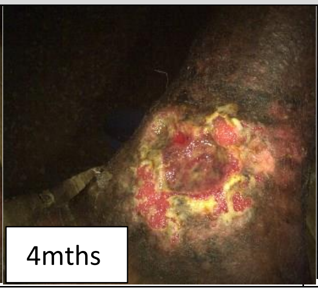

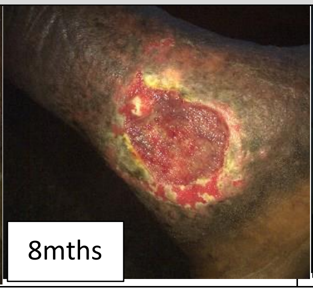

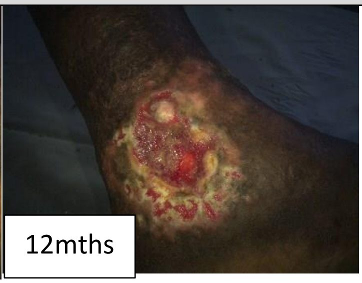

Case Report OBOM

Chronic Buruli Ulcer Wounds

Patient No. 009

1. Demographic data

Sex: Female
Age: 36 years

2. Wound description

BU Category III (see Photo documentation)

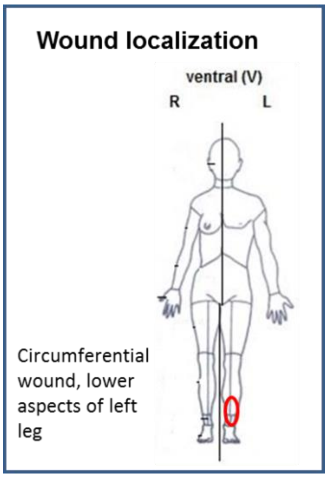

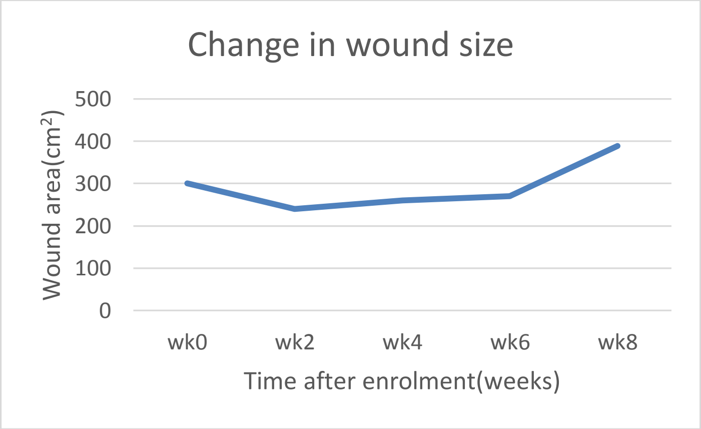

3. Medical History

Nil of significance

<u>Wound History:</u>

Wound observed since: 05/2010

BU- Treatment: 06/11/13- 02/01/14

Wound non healing

Tissue infection

4. Physical examination

Body- Mass- index (BMI) 38.97 kg KG/m²

All systems normal

5. Current Medication

Streptomycin 1000mg, Rifampicin 600mg. 56 doses taken

Diclofenac

Oral Cefuroxime (7days) for wound infection

6. Laboratory

BU confirmation: PCR (+) ZN (-) for *M. ulcerans*

Retroscreen: Non reactive

7. Microbiology

Wound C/S: *Providencia stuartii* cultured (6/11/13)

8. Photo documentation:

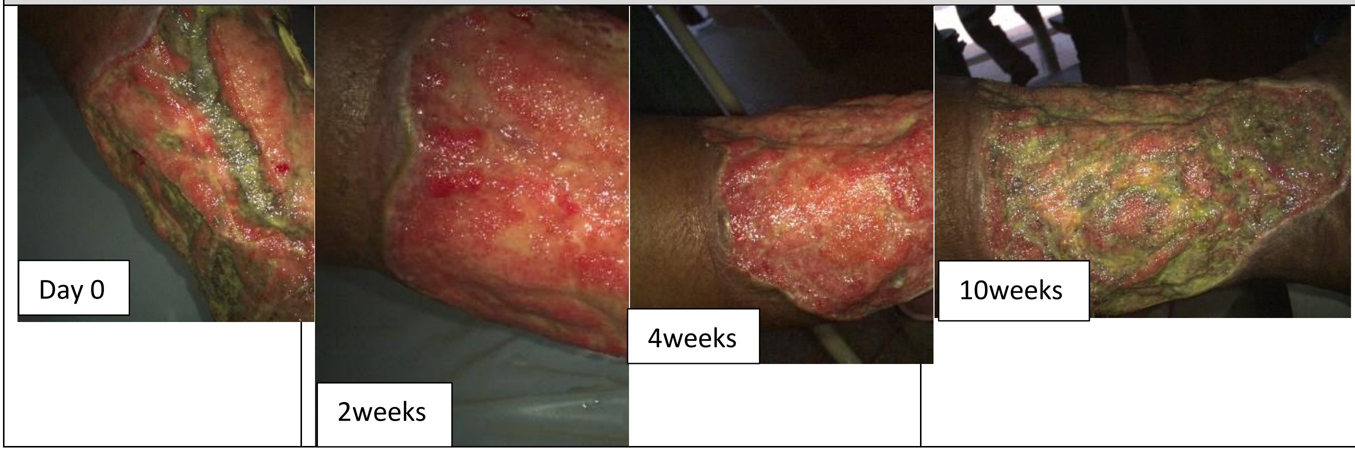

Case Report OBOM

Chronic Buruli Ulcer Wounds

Patient No. 012

1. Demographic data

Sex: Female
Age: 32 years

2. Wound description

BU Category II (see Photo documentation)

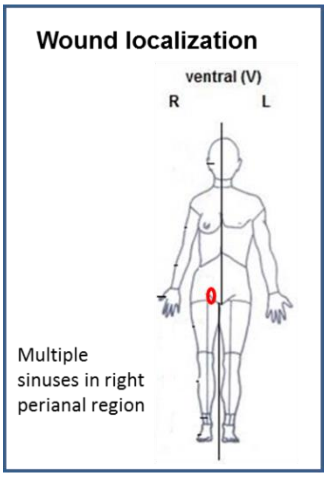

3. Medical History

Nil of significance

Wound History:

Wound observed since: 11/2010

BU- Treatment: 14/11/13- 06/12/13

Chronic osteomyelitis suspected

Patient was lost to follow-up after 2 weeks of wound monitoring.

4. Physical examination

Body- Mass- index (BMI) 21.78 kg KG/m²

All systems normal

5. Current Medication

Streptomycin 1000mg, Rifampicin 600mg. 22 doses taken

Patient had been on BU treatment for 6 days before enrolment

6. Laboratory

BU confirmation: PCR (+) ZN (+) for *M. ulcerans*

Retroscreen: Non reactive

7. Photo documentation:

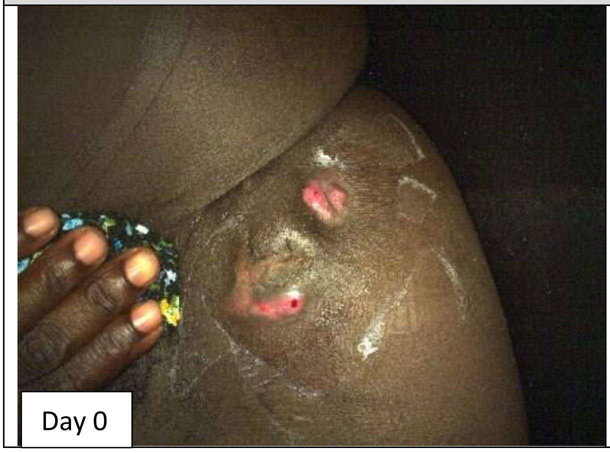

Day 0

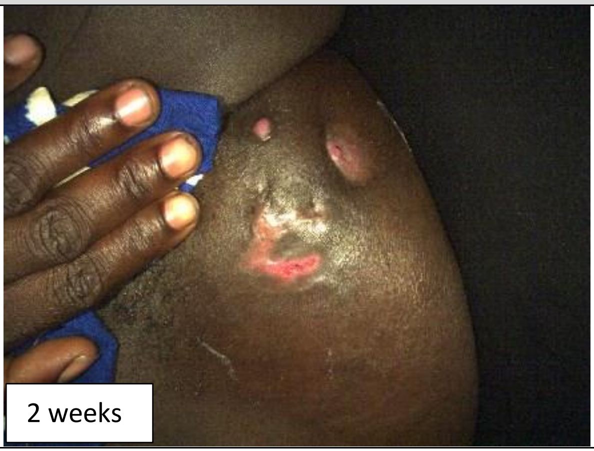

2 weeks

Case Report OBOM

Chronic Buruli Ulcer Wounds

Patient No. 014

1. Demographic data

Sex: Female
Age: 60 years

2. Wound description

BU Category II (see Photo documentation)

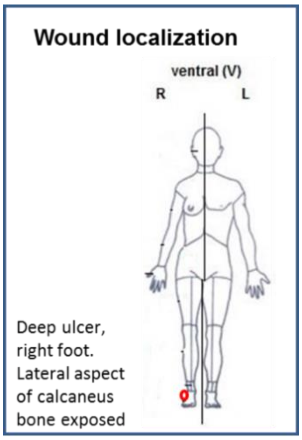

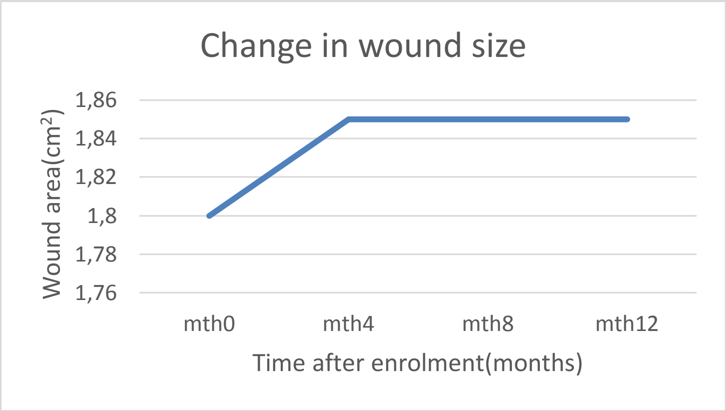

3. Medical History

Asthmatic

<u>Wound History:</u>

Wound observed since: 01/2011

BU- Treatment: 16/02/11- 10/04/11

Non- healing wound

.

4. Physical examination

Body- Mass- index (BMI) 18.73 kg KG/m²

All systems normal

5. Current Medication

Streptomycin 750mg, Rifampicin 450mg. 56 doses taken

Patient had completed BU treatment 3 years before enrolment

6. Laboratory

BU confirmation: PCR (+) ZN (-) for *M. ulcerans*

Retroscreen: Non reactive

7. Photo documentation:

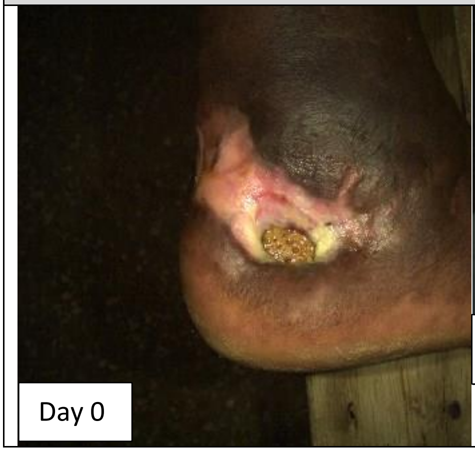

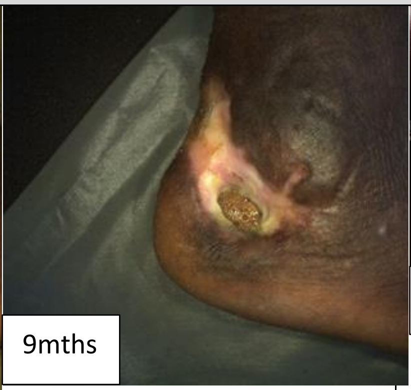

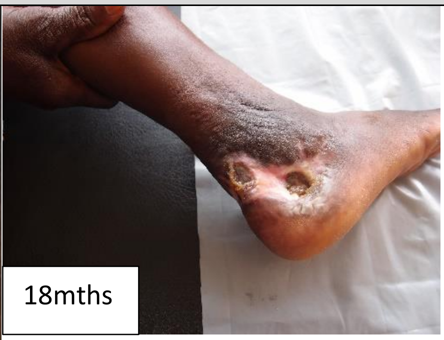

Case Report OBOM

Chronic Buruli Ulcer Wounds

Patient No. 011

1. Demographic data

Sex: Female
Age: 15 years

2. Wound description

BU Category II (see Photo documentation)

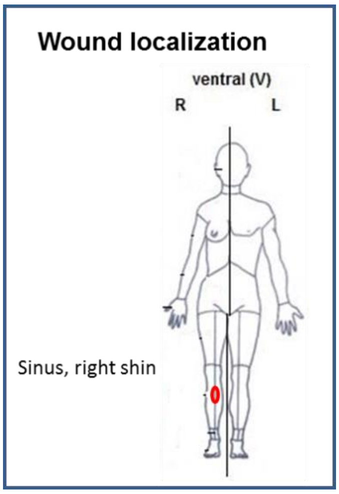

3. Medical History

Nil of significance

Wound History:

Wound observed since: 11/2011

BU- Treatment: 13/11/13- 15/01/14

Chronic osteomyelitis (confirmed by X-ray- see below)

.

4. Physical examination

Body- Mass- index (BMI) 18.96 kg KG/m²

All systems normal

5. Current Medication

Streptomycin 750mg, Rifampicin 450mg. 56 doses taken

Ciprofloxacin (6weeks on account of osteomyelitis)

**6. Laboratory**
BU confirmation: PCR (+) ZN (-) for *M. ulcerans*<br>Retroscreen: Non reactive
**7. Microbiology**
Repeated (3x) cultures of sinus aspirate: *Proteus mirabilis* isolated
**8. Photo documentation:**
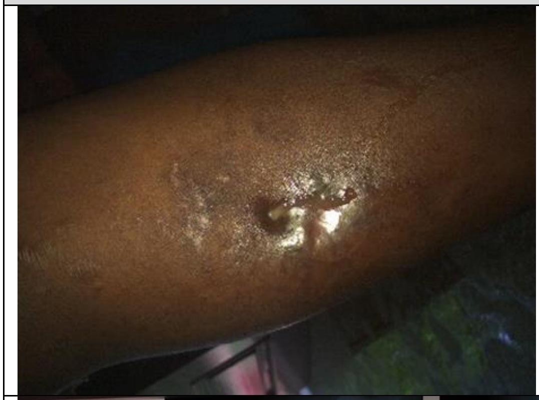
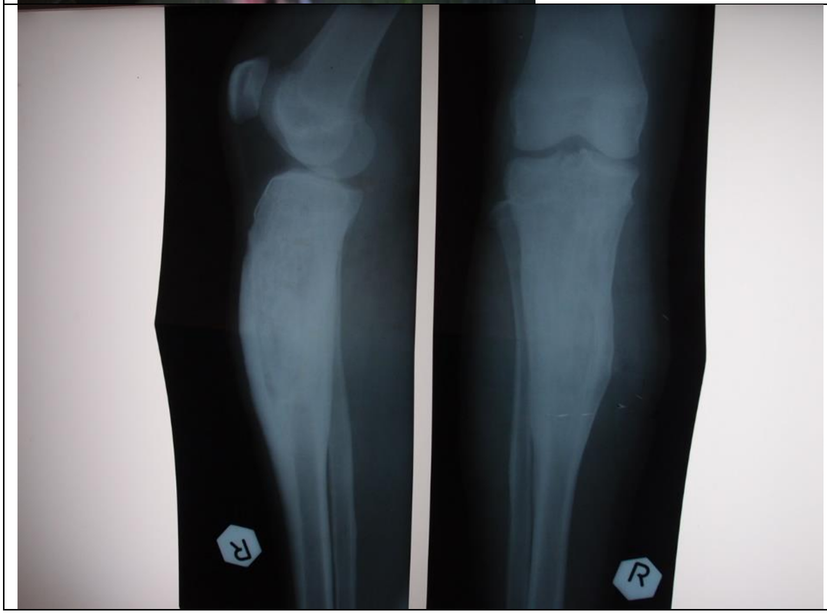

Case Report OBOM

Chronic Buruli Ulcer Wounds

Patient No. 020

1. Demographic data

Sex: Male
Age: 33 years

2. Wound description

BU Category III (see Photo documentation)

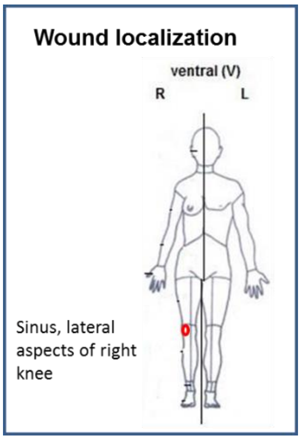

3. Medical History

Nil of significance

<u>Wound History:</u>

Wound observed since: 06/2013

BU- Treatment: 05/02/14- 02/04/14

Chronic osteomyelitis (confirmed by X-ray- see below)

.

4. Physical examination

Body- Mass- index (BMI) 22.05 kg KG/m²

All systems normal

5. Current Medication

Streptomycin 1000mg, Rifampicin 600mg. 56 doses taken

Ciprofloxacin (6weeks on account of osteomyelitis)

Rifampicin for additional 6 weeks on account of osteomyelitis

6. Laboratory

BU confirmation: PCR (+) ZN (+) for *M. ulcerans*

Retroscreen: Non reactive

7. Microbiology

Repeated (3x) cultures of sinus aspirate: *S.aureus* isolated

8. Photo documentation:

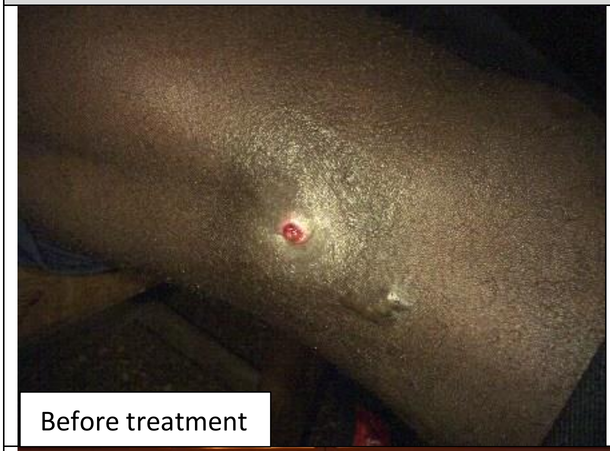

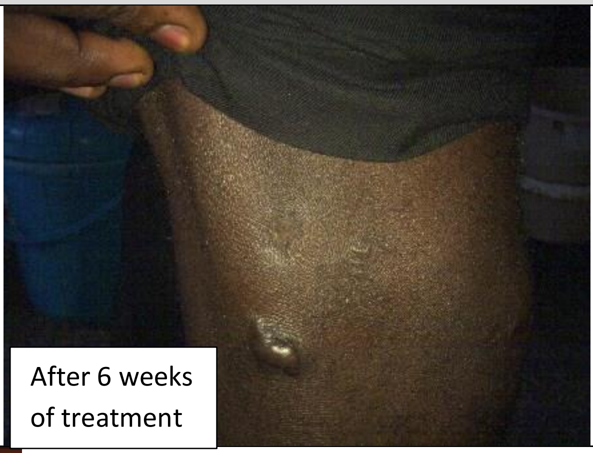

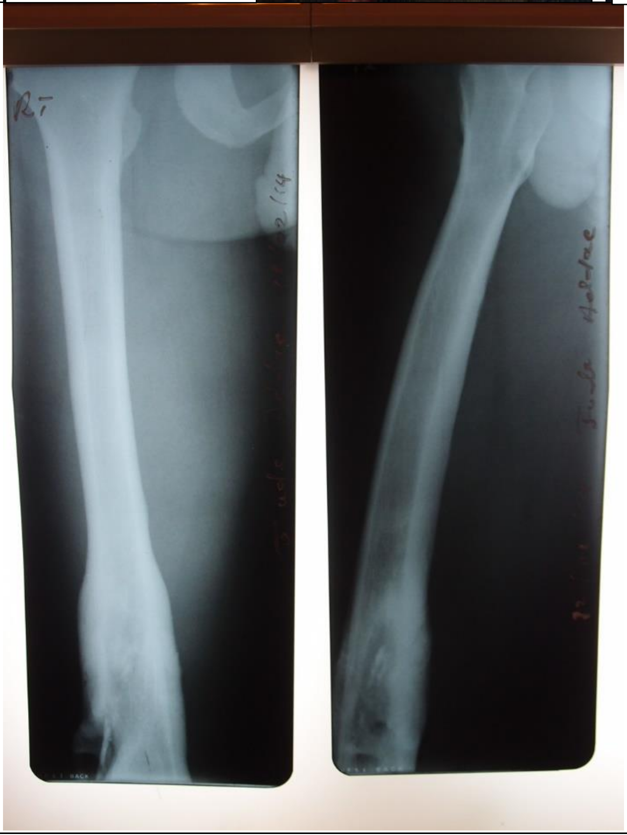

Case Report OBOM

Chronic Buruli Ulcer Wounds

Patient No. 005

1. Demographic data

Sex: Female
Age: 22 years

2. Wound description

BU Category III (see Photo documentation)

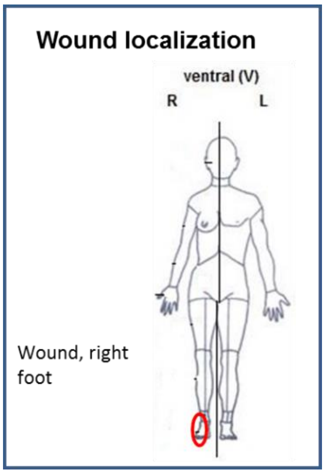

3. Medical History

Nil of significance

<u>Wound History:</u>

Wound observed since: 02/2013

Wounds at the same localization on 2 previous occasions:
2001- 2004 (healed)
01/2010- 03/2011 (healed)

BU- Treatment: 01-03/2011

02/2014 wound biopsy for histopathology
Results: well differentiated invasive squamous cell carcinoma

02/2014 chest X-ray: "lung fields look normal"

X-ray of right foot: 2 lateral digits together with metatarsals absent

4. Physical examination

Body- Mass- index (BMI) 16.73 kg KG/m²

Very pale, slightly jaundiced
Hepatomegaly, 2 cm below costal margin.
Spleen not palpable.
Inguinal lymph nodes enlarged

5. Current Medication

Streptomycin 1000mg, Rifampicin 600mg. 56 doses taken

Diclofenac, Tramadol, Fersolate, Vitamin C

6. Laboratory

BU confirmation: PCR (+) ZN (+) for *M. ulcerans*

Retroscreen: Non reactive

Hb: 5.5g/dl

7. Microbiology

Wound culture: *Proteus Mirabilis* isolated

8. Photo documentation:

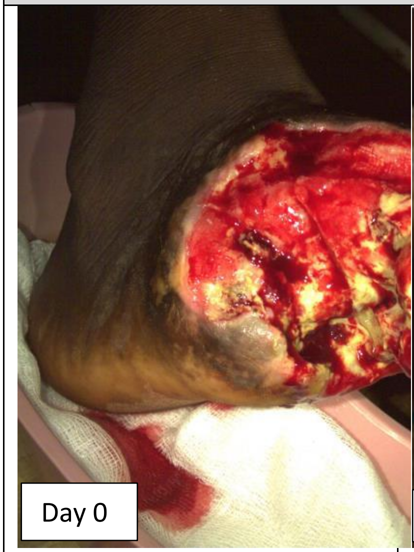

Day 0

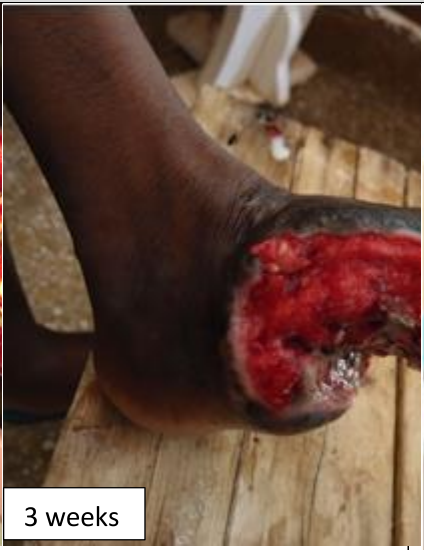

3 weeks

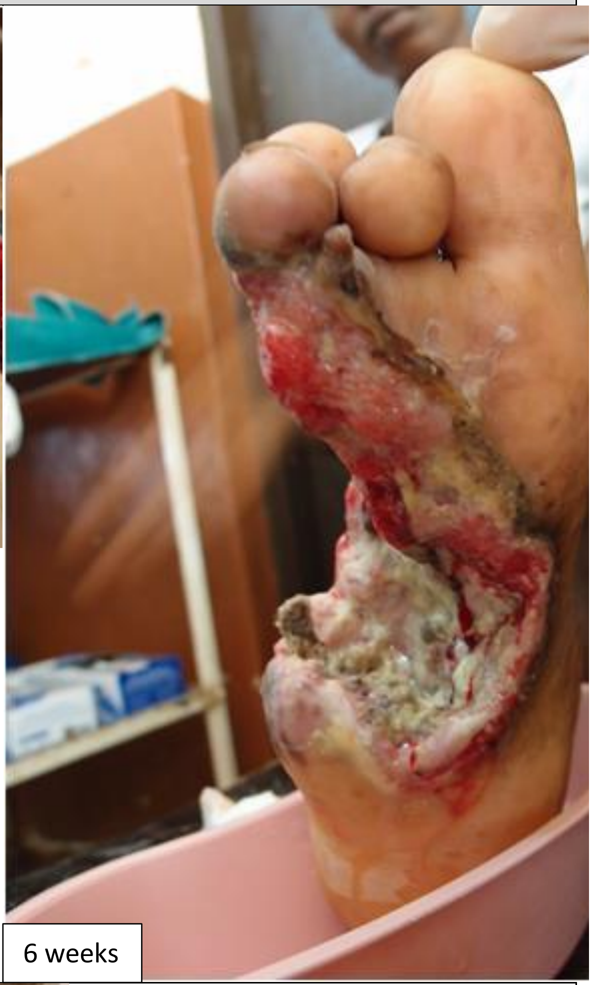

6 weeks

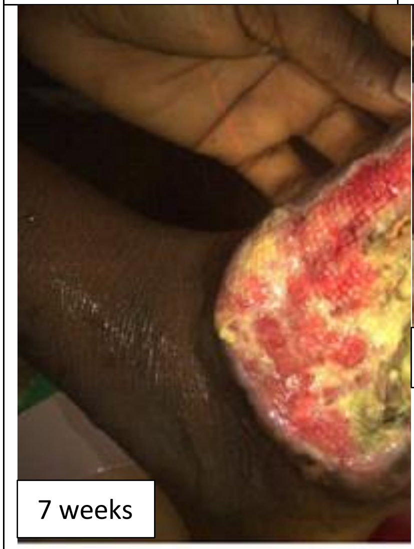

7 weeks

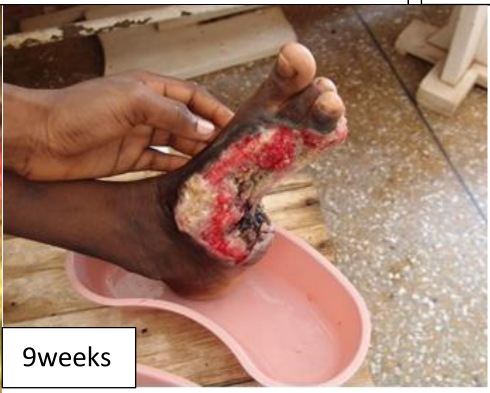

9weeks
