# Supplementary material for: Assessing and managing wounds of Buruli ulcer patients at the primary and secondary health care levels in Ghana
Source: PLoS Negl Trop Dis. 2017 Feb 28;11(2):e0005331. doi: 10.1371/journal.pntd.0005331 (PMC5345880; doi:10.1371/journal.pntd.0005331)
Supplement: S4 Case Report — (PDF) [file pntd.0005331.s006.pdf]

Case Report AMH

Sub-chronic Buruli Ulcer Wounds

Patient No. 001

1. Demographic data

Sex: Male
Age: 15 years

2. Wound description

BU Category III (see Photo documentation)

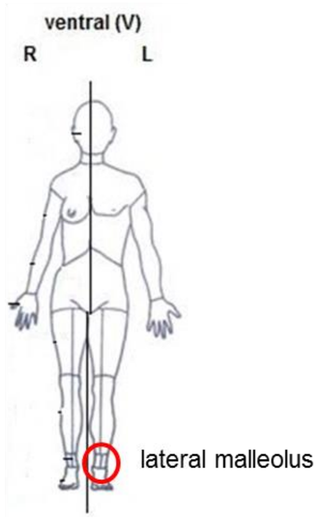

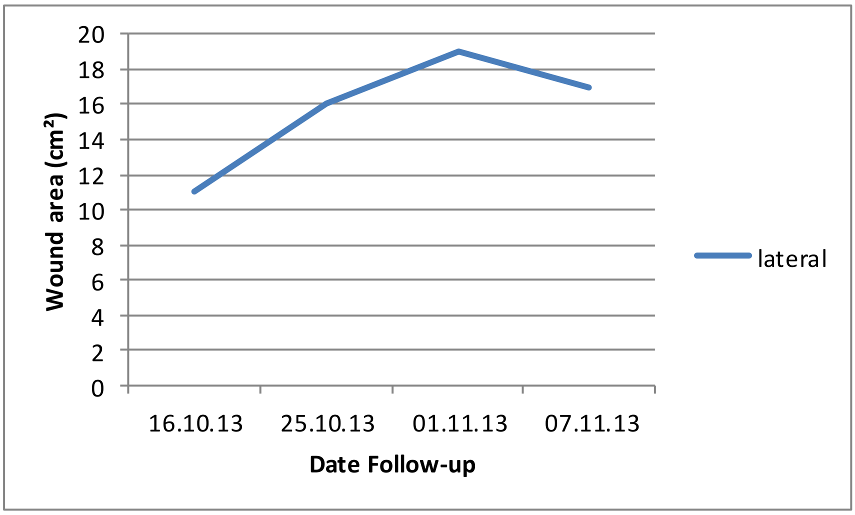

3. Medical History

Wound observed since: 04/2013

since 09/2013 hospitalized at Municipal Hospital Amasaman

09-11/2013 Antimycobacterial treatment with Rifampicin 300mg, Streptomycin 500mg (56d)
08/11/2013 wound excision
29/11/2013 split skin grafting

Secondary diagnoses
No further diagnosis

4. Physical examination

Body- Mass- index (BMI) 15,79 kg KG/m²

All systems normal

Pain assessment: intermittent, severe pain at wound and wound surrounding (8/10)

5. Current Medication

No analgesic therapy

6. Laboratory

BU confirmation: 09/2013 ZN+/ PCR+ for M.ulcerans

**Date**	**05.11.13**
Hb [12-16g/dl]	13,2 g/dl
HCT [37-51%]	39,10 %
RBC [4,2- 6,3M/ul]	5,72 M/ul
MCV [80-97fl]	68,4 fl
MCH [26-32 pg]	23,1 pg
MCHC [31-36 g/dl	33,8 g/dl
WBC [4.1-10.9 K/ul]	8,2 K/ul
ESR [5-15mmfall/h]	
PLT [140-440 K/ul]	
Sickling	negative

7. Microbiology

Results of wound swabs

11/2013 Pseudomonas aeruginosa: sensitive to Amikacin, Gentamicin,Tobramycin

8. Photo documentary: Ulcer left leg, lateral malleolus

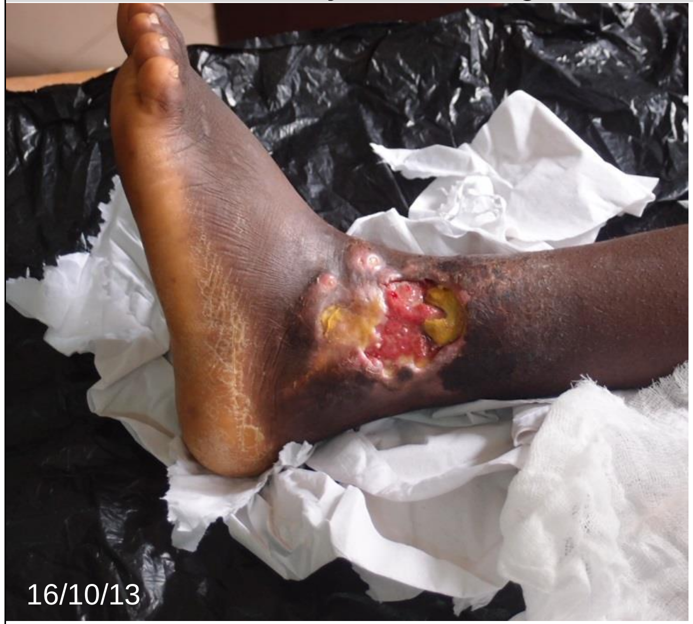
16/10/13

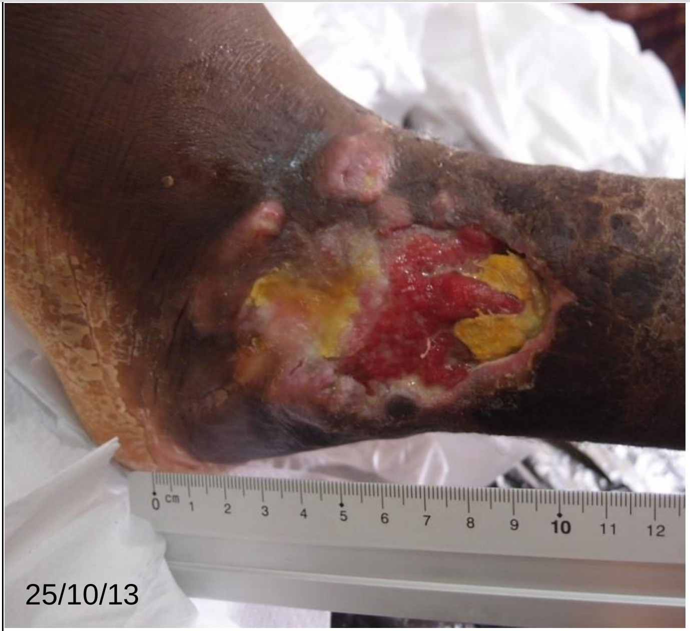
25/10/13

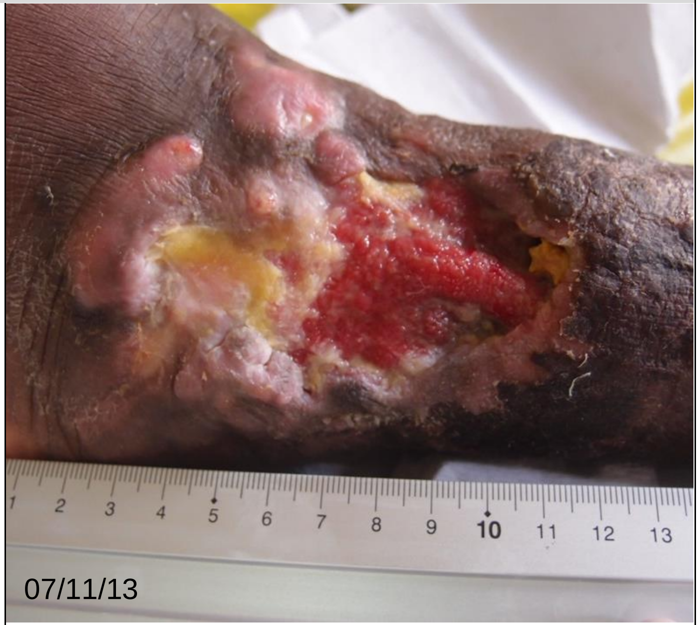
07/11/13

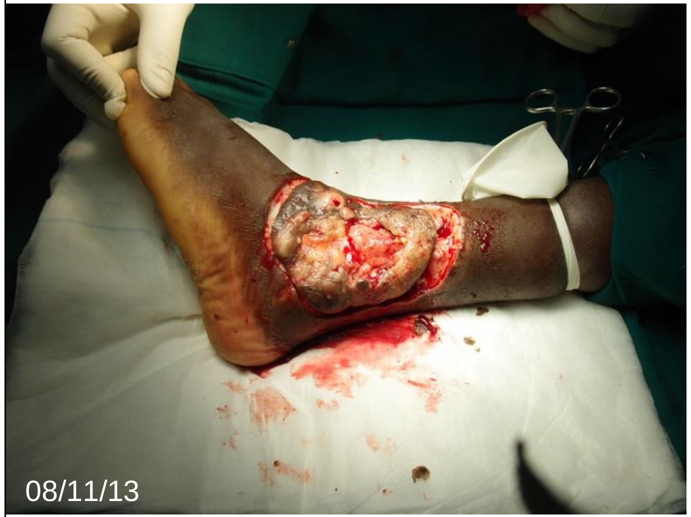
08/11/13

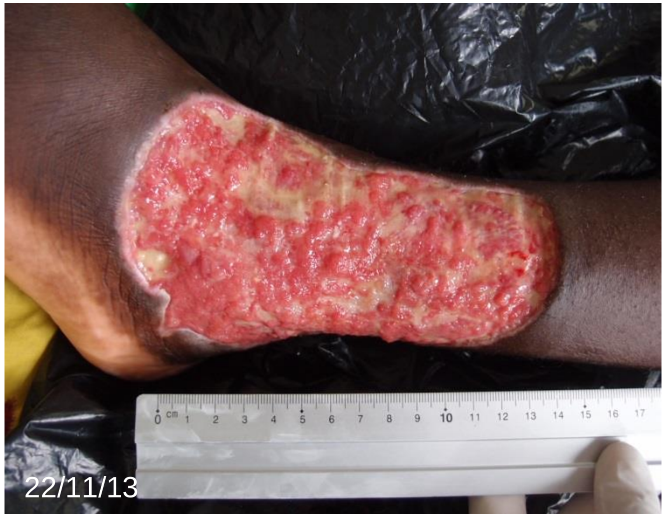
22/11/13

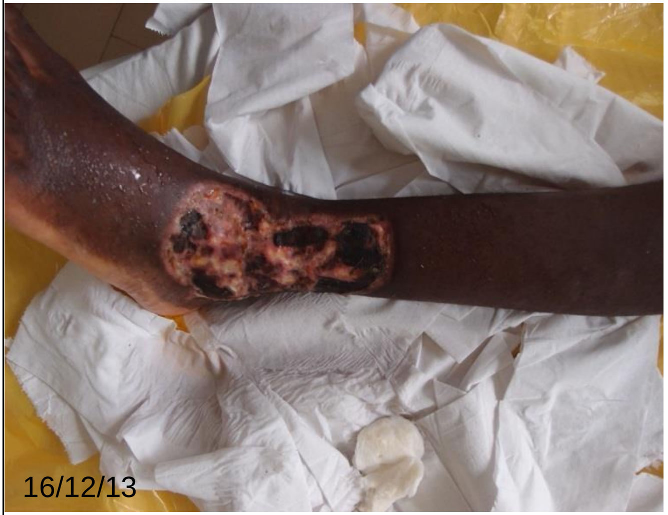
16/12/13

Case Report AMH

Chronic Buruli Ulcer Wounds

Patient No. 002

1. Demographic data

Sex: Female
Age: 27 years

2. Wound description

BU Category III (see Photo documentation)

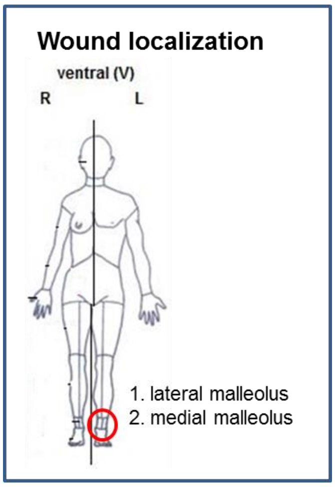

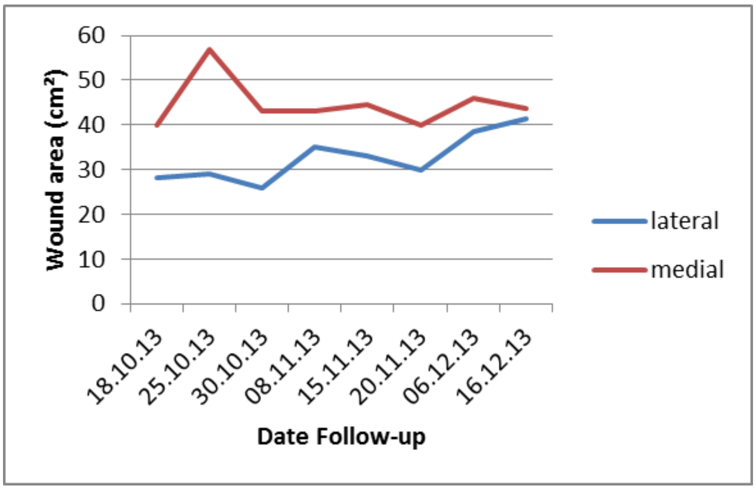

3. Medical History

<u>Secondary diagnoses</u>
04/2013 Cellulites left leg
05/2013 Gastritis, triple treatment *H. pylori*
01/2014 Microcytic hypochromic anaemia
01/2014 Infarction on the left in the temporo-parietal region adjacent the lateral ventricle with right facial paralysis, motoric dysfunction of the right arm
02/2014 two pressure ulcers at gluteal region

<u>Wound History</u>

Wound observed since: medial: 09/2011
lateral: 03/2012

hospitalized at Municipal Hospital Amasaman:
since 09/2012

09-10/2012	BU treatment
11/2012	wound excision on both sides
02/2013	split skin grafting on both sides

4. Physical examination

Body-Mass-Index (BMI) 26,67 kg KG/ m²

All systems normal

Pain assessment: constant, severe pain at wound and wound surrounding (7-9/10)

<u>Movement of ankle joint</u>
(dorsal extension/ plantar flexion)

left	10-0-40
right	20-0-40

5. Current Medication

Streptomycin 100mg, Rifampicin 600mg. 20 doses taken
Diclofenac 50mg + Paracetamol 500 (1-1-1)
Folic acid, Vitamin B complex
Zincofer

6. Laboratory

BU confirmation: 09/2012 ZN (+) PCR(+) for *M. ulcerans*

**Date**	**24.01.14**	**05.02.14**
Hb [12-16g/dl]	7,4 g/dl	
HCT [37-51]	23,8%	
RBC [4,2- 6,3M/ul]	3,25 M/ul	
MCV [80-97fl]	73,2 fl	
MCH [26-32 pg]	22,8 pg	
MCHC [31-36 g/dl	31,1 g/dl	
WBC [4.1-10.9 K/ul]<br>- Neutrophils [25-75%]<br>- Lymphocytes [20-60%]<br>- Monocytes [2-10%]<br>- Eosinophils [1-6%]<br>- Basophils [0-1%]	7,5 K/ul<br>- 68,6%<br><br>- 27,2%	
PLT [140-440 K/ul]	345 K/ul	
Urea [2,1- 7,1 mmol/l]		4,8 mmol/l
Creatinine [53- 106 umol/l]		102 umol/l
Albumin [35-52 g/l]		56 g/l
T.Protein [60-78 g/l]		106 g/dl
T.Bilirubin [5-21 umol/l]		15,9 umol/l
D.Bilirubin [<3,4 umol/l]		8,5 umol/l
AST [<40U/l]		22 U/l
ALT [<40U/l]		23 U/l
Gamma GT [6-24 U/l]		22 U/l
ALKP [<240U/L]		145 U/l

7. Microbiology

Results of wound swabs

02/2013 *S. aureus*: sensitive to Amikacin, Gentamicin, Tetracylin
11/2013 *Aeromonas salmonicida*: sensitive to Amikacin, Gentamicin, Ceftriaxone, Tobramycin

8. Photo documenation: Ulcer left leg, lateral malleolus

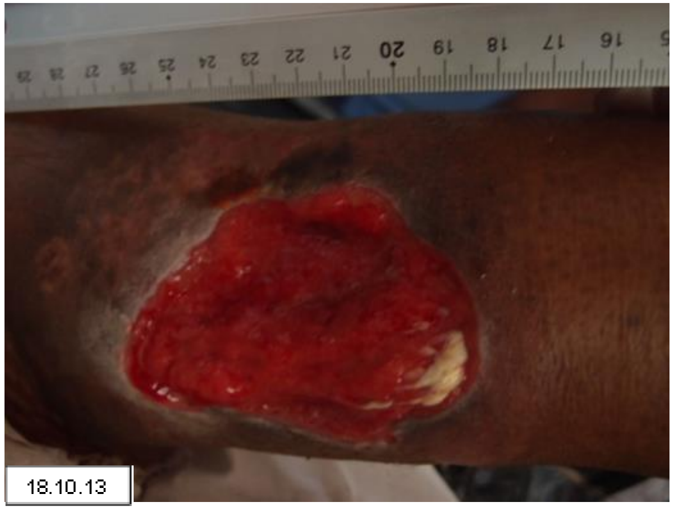

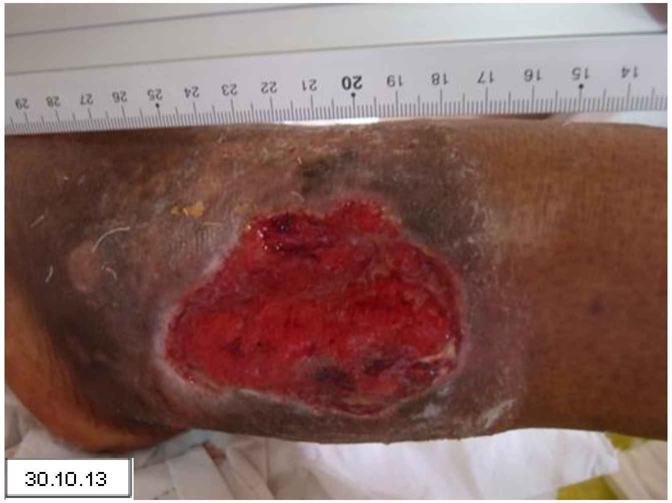

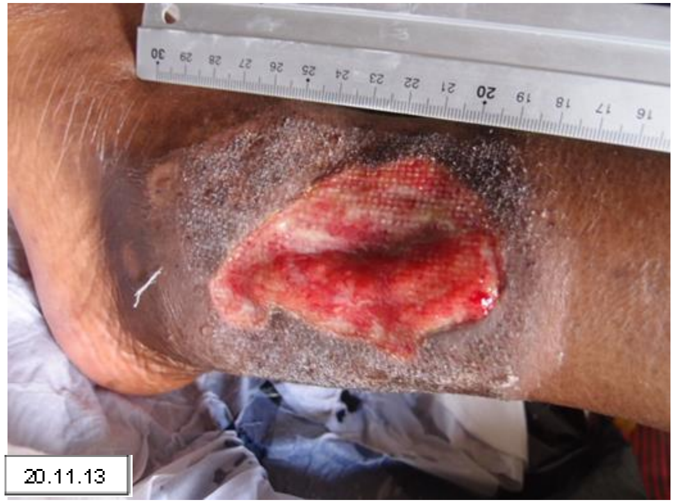

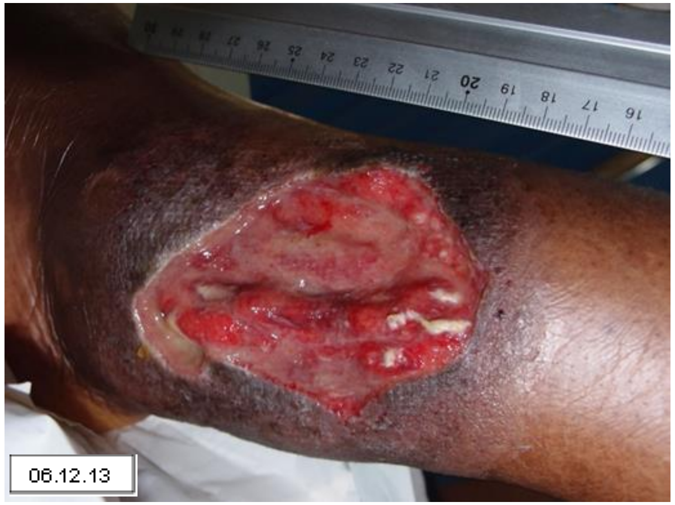

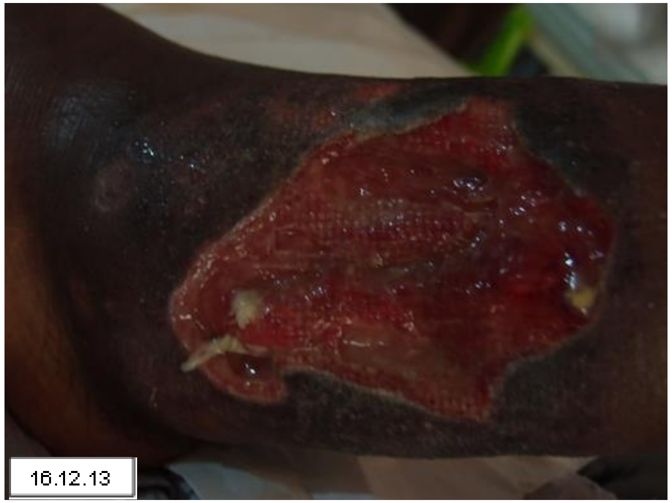

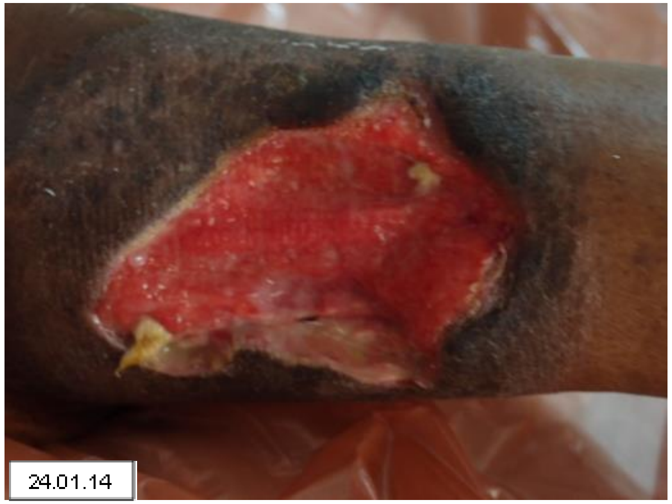

9. Photo documentation: Left leg, medial malleolus

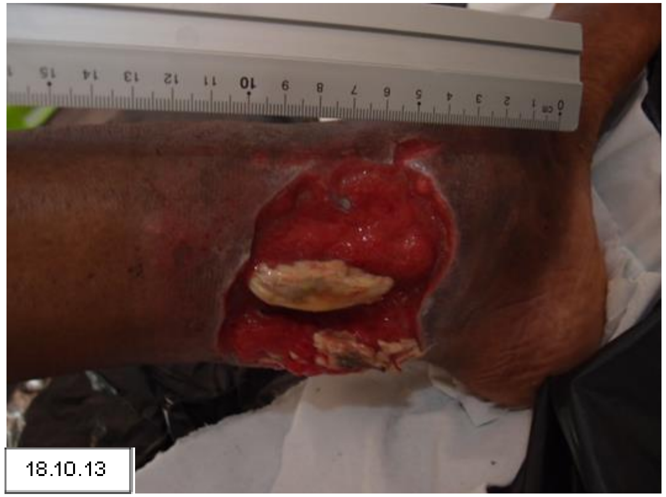

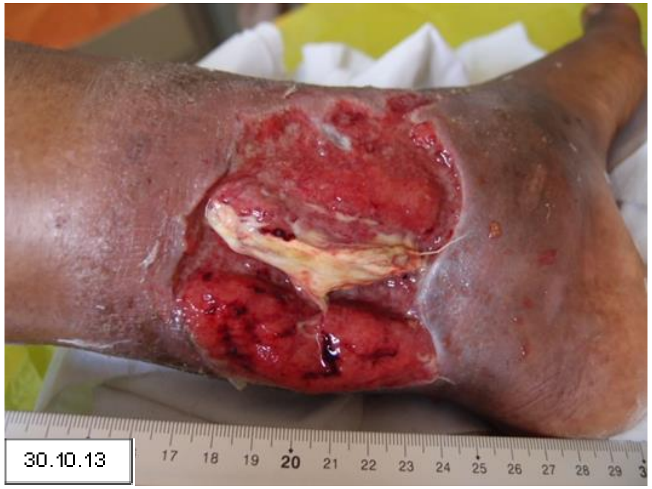

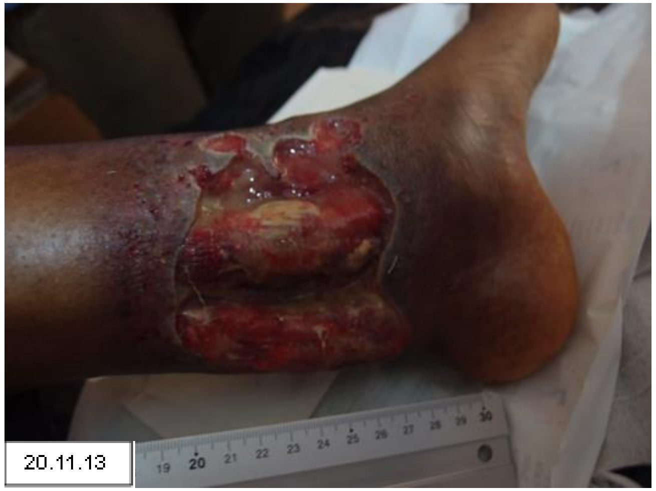

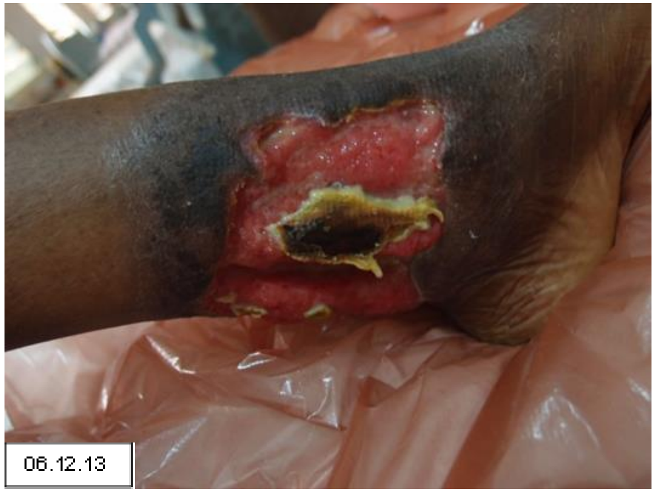

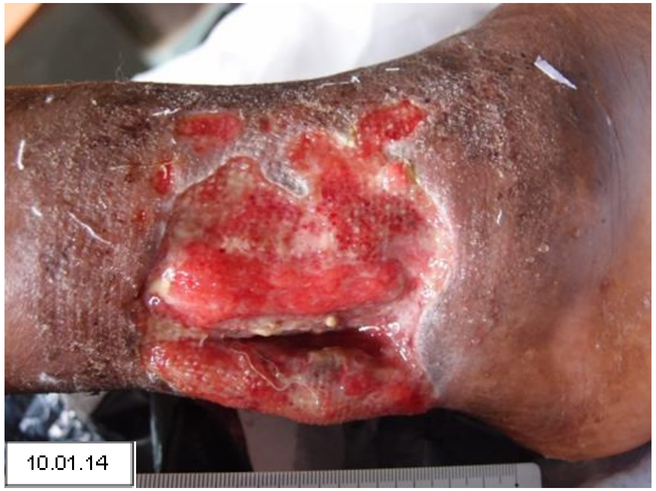

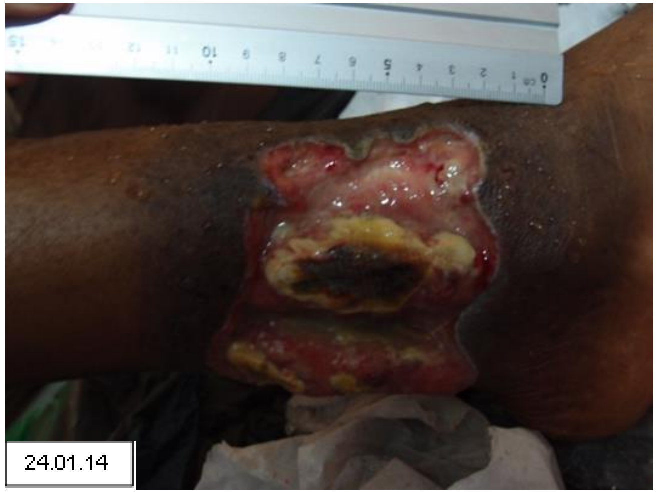

Case Report AMH

Chronic Buruli Ulcer Wounds

Patient No. 003

1. Demographic data

Sex: Female
Age: 56 years

2. Wound description

BU Category III (see Photo documentation)

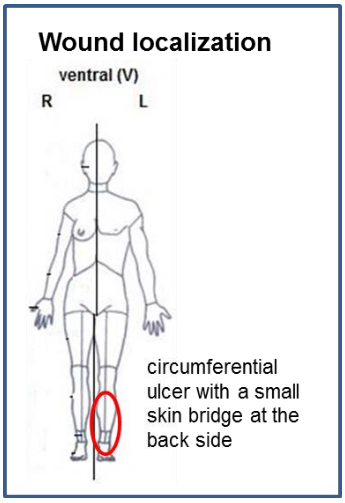

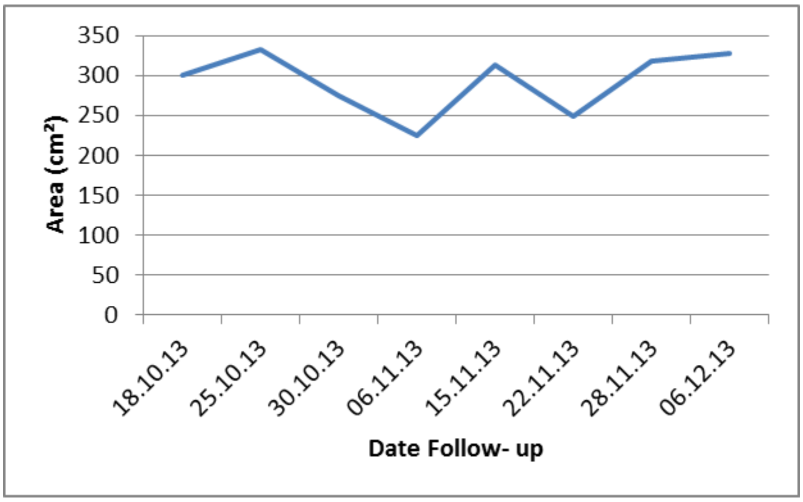

3. Medical History

<u>Wound history</u>

Wound observed since: around 2009

since 07/2013 hospitalized at Municipal Hospital Amasaman

07-08/2013 Antimycobacterial treatment with Rifampicin 600mg, Streptomycin 1000mg (54d)

08/2013 wound excision

<u>Secondary diagnoses</u>

08/2013 tremor on the right hand of unknown reason

4. Physical examination

Body-Mass-Index (BMI) 35,38 kg KG/ m²

All systems normal

Pain assessment: severe pain at wound and wound surrounding especially during dressing (7/10)

5. Current Medication

Diclofenac 50mg + Paracetamol 500 (1-0-0)
Vitamin B complex
Zincofer
Erythromycin 500mg (2-2-2-2) against sec. bacterial wound infection

6. Laboratory

BU confirmation 06/2013 ZN+/ PCR- for *M. ulcerans*

7. Microbiology

Results of wound swabs in the period (06-11/2013)

S. aureus: sensitive to Ceftriaxone
Pseudomonas aeruginosa: sensitive to Amikacin, Gentamicin, Ceftriaxone, Tobramycin

8. **Photo documentation: Ulcer distal left leg** (shows dorsal side of the wound)

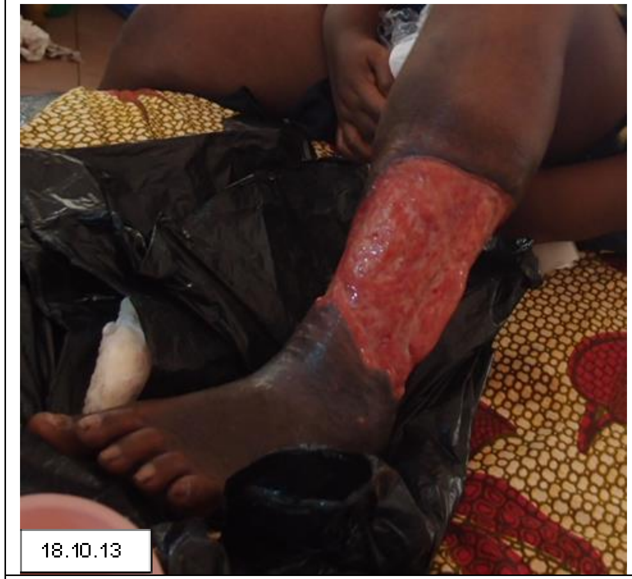

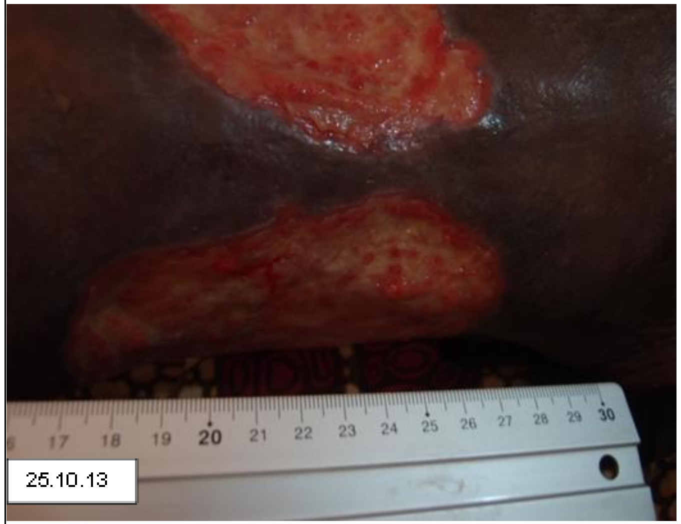

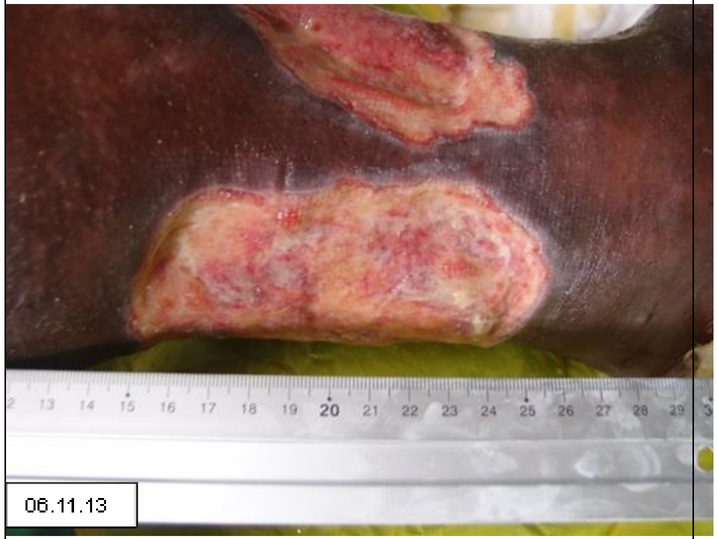

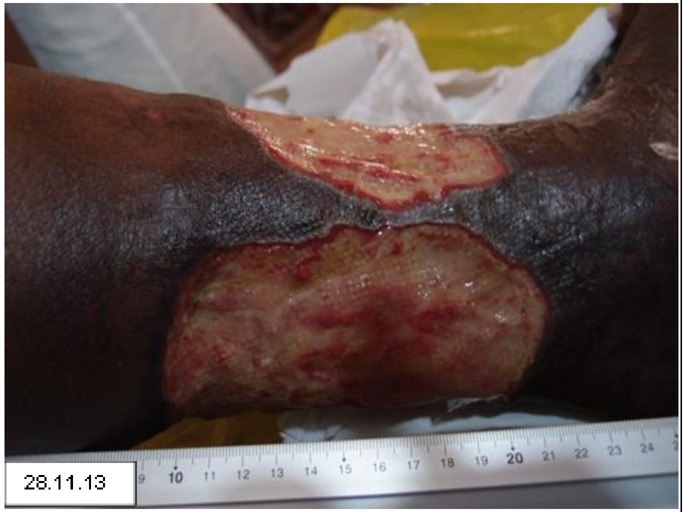

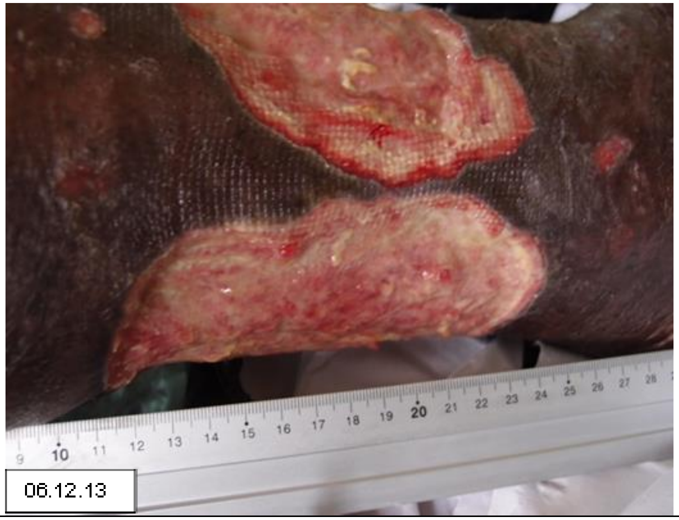

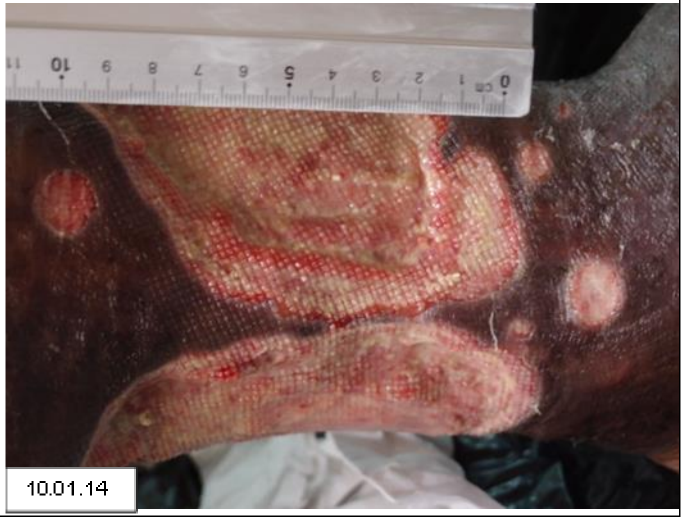

Case Report AMH

Chronic Buruli Ulcer Wounds

Patient No. 004

1. Demographic data

Sex: Female
Age: 38 years

2. Wound description

BU Category III (see Photo documentation)

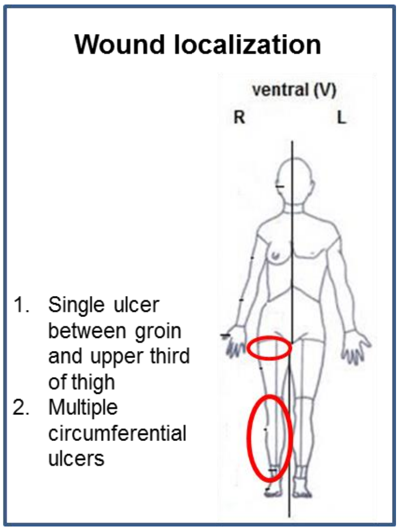

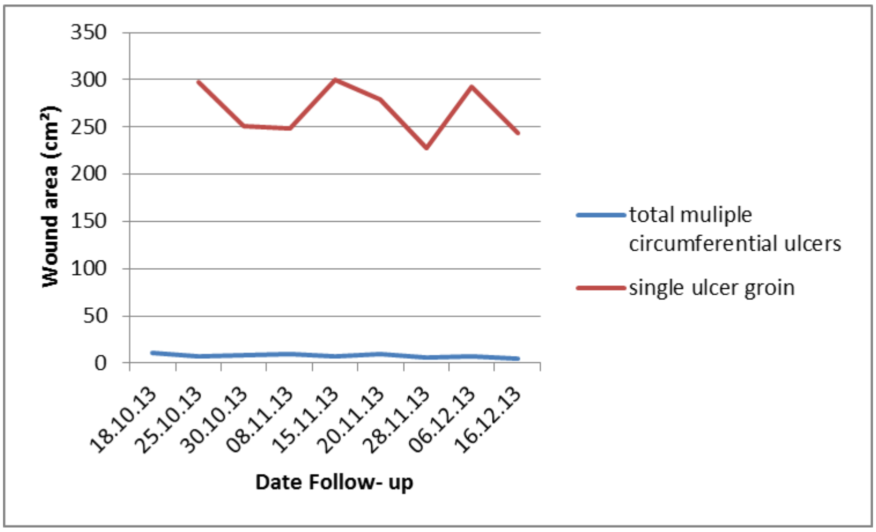

3. Medical History

<u>Wound history</u>

Wound observed since: both 03/2012

since 03/2013 hospitalized at Municipal Hospital Amasaman

03-05/2013 Antimycobacterial treatment
06-07/2013 with Rifampicin 600mg, Streptomycin 1000mg (55+ 30d)
08/2013 multiple wound excision

<u>Secondary diagnoses</u>
HIV positive since several years

4. Physical examination

Body-Mass-Index (BMI) 24,7 kg KG/ m²

All systems normal

Pain assessment: moderate till severe pain on the whole leg (4-7/10)

5. Current Medication

Antiretroviral treatment: Tenofovir/ Lamivudine 300mg, Nevirapine USP 200mg (2-0-1)
Cotrimoxazole (1-0-0)
Multivitamines
Zincofer

6. Laboratory

BU confirmation: 06/2013   ZN+/ PCR+ for *M. ulcerans*

Date	21.08.13	05.11.13
Hb [12-16g/dl]		8,6 g/dl
HCT [37-51%]		29,6%
RBC [4,2- 6,3M/ul]		4,02 M/ul
MCV [80-97fl]		73,6 fl
MCH [26-32 pg]		21,4 pg
MCHC<br>[31-36 g/dl		29,1 g/dl
WBC<br>[4.1-10.9 K/ul]		4,7 K/ul
PLT<br>[140-440 K/ul]		194 K/ul
CD4	185/182	
CD3	1332/1308	

7. Microbiology

Results of wound swabs

Pseudomonas aeruginosa: sensitive to Amikacin, Gentamicin, Ceftriaxone, Tobramycin

8. Photo documentation

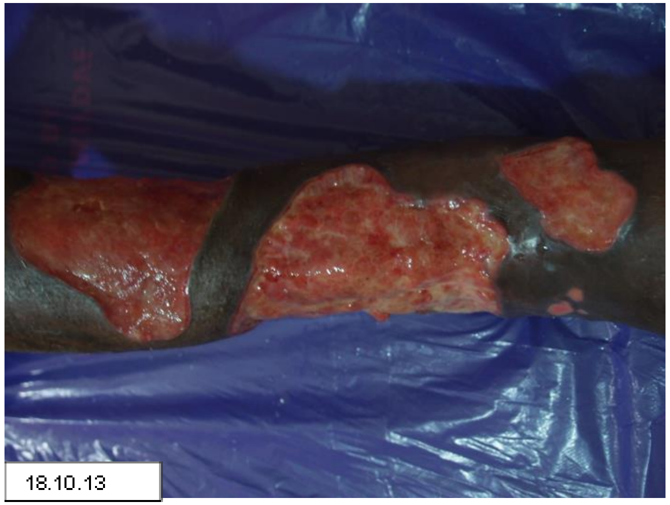

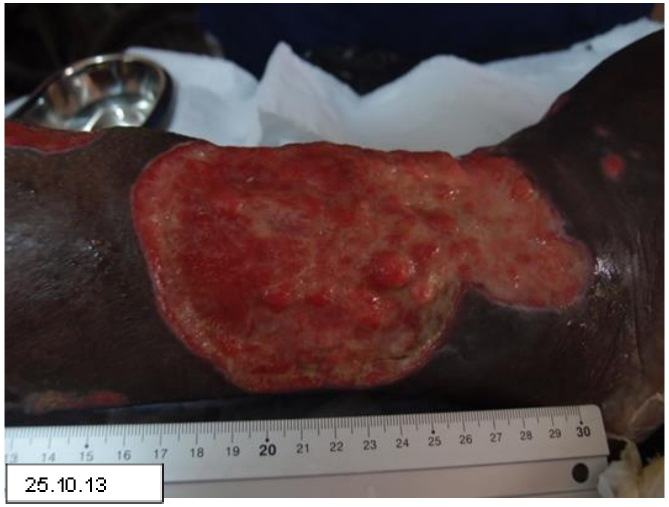

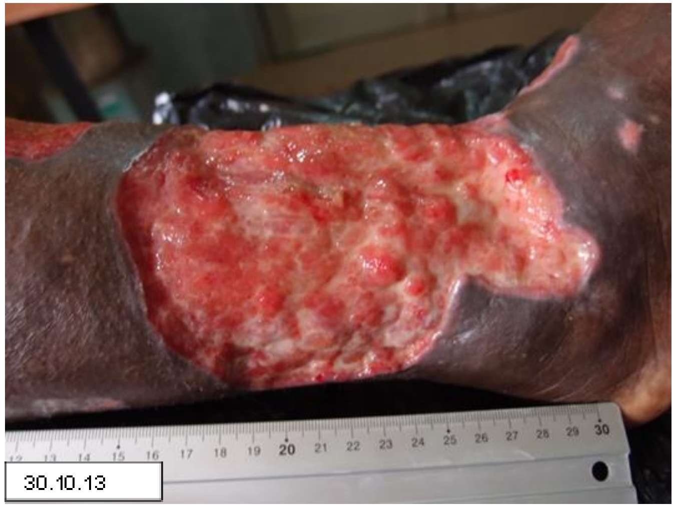

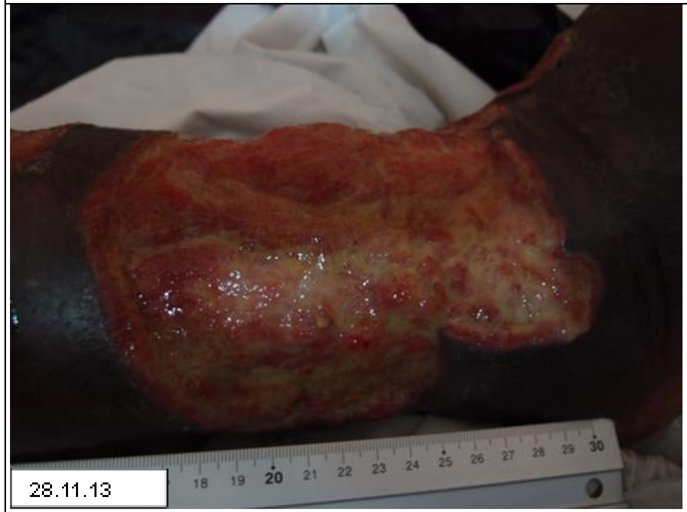

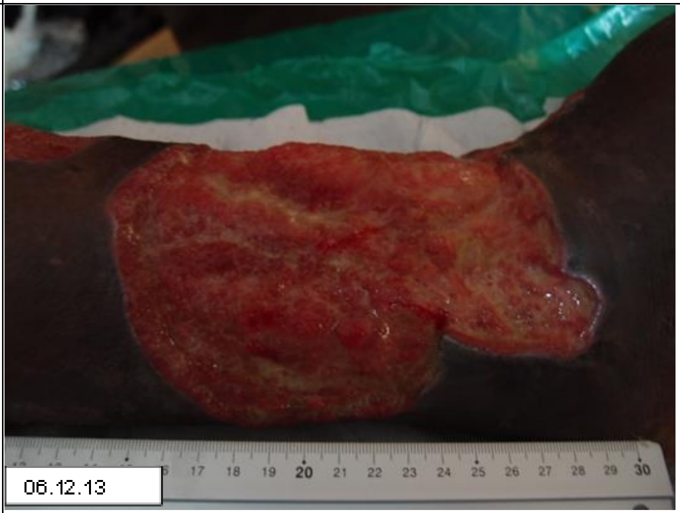

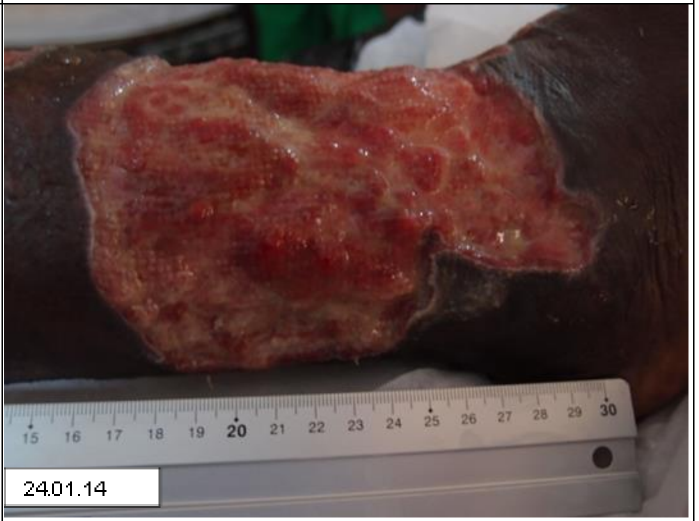

Case Report AMH

Healed Buruli Ulcer Wounds

Patient No. 005

1. Demographic data

Sex: Male
Age: 45 years

2. Wound description

BU Category III (see Photo documentation)

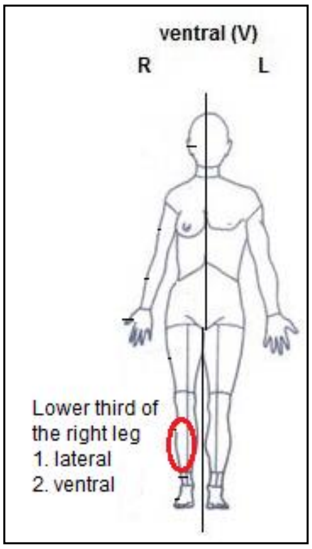

3. Medical History

Wound history

Wound observed since:06/2012

since 05/06/13 hospitalized at Municipal Hospital Amasaman

23/3- 12/5/13 Antimycobacterial treatment with Rifampicin 600mg, Streptomycin 1000mg (55+ 30d)

08/11/2013 split skin grafting

Secondary diagnoses
Diabetes mellitus

4. Physical examination

Body-Mass-Index (BMI) 33.6 kg KG/ m^2

All systems normal

5. Current Medication

Metformin 1000mg

6. Laboratory

BU confirmation: ZN (-) PCR (+) for *M. ulcerans*

7. Photo documentation: Ulcer right leg, lateral

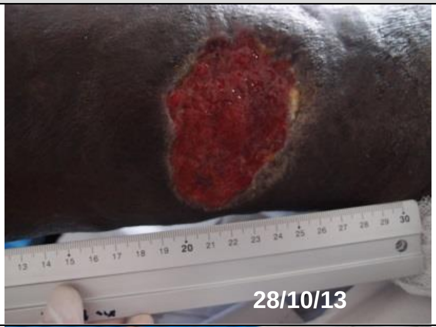

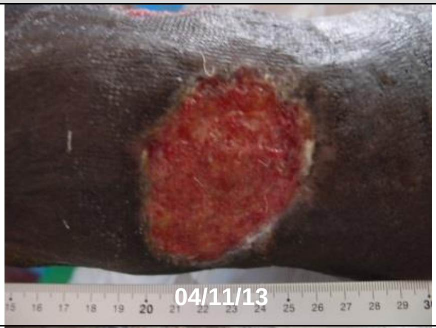

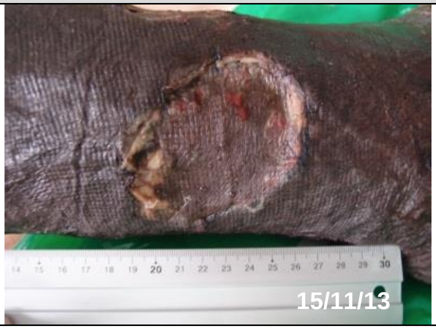

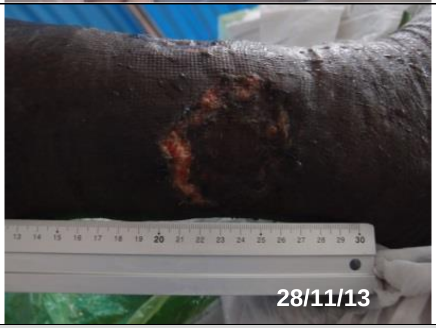

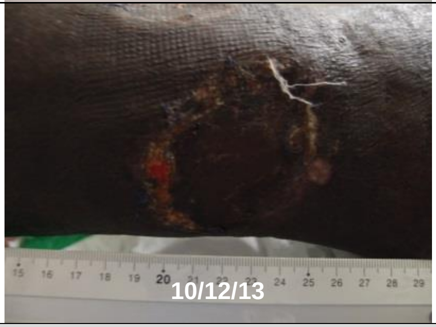

Ulcer right leg, ventral

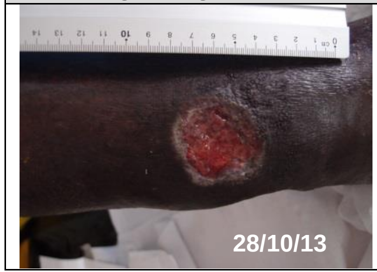

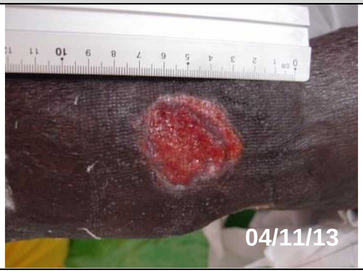

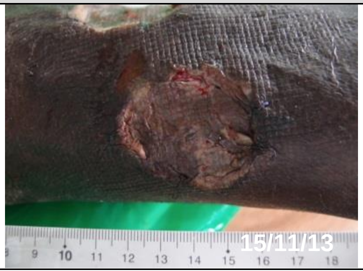

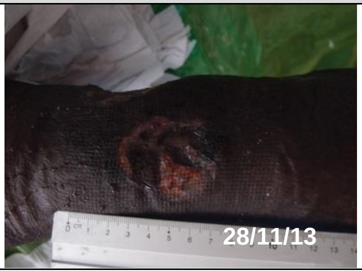

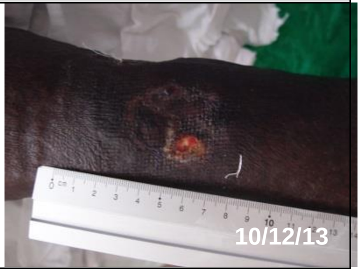

Case Report AMH

Healed Buruli Ulcer Wounds

Patient No. 006

1. Demographic data

Sex: Male
Age: 55 years

2. Wound description

BU Category III (see Photo documentation)

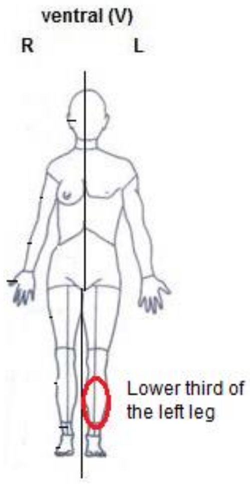

3. Medical History

<u>Wound history</u>

Wound observed since: 2011

since 03/06/2013 hospitalized at Municipal Hospital Amasaman

08/11/2013 wound excision and SSG

<u>Secondary diagnoses</u>
Varicose veins

4. Physical examination

Body-Mass-Index (BMI) 22.96 kg KG/ m^2

All systems normal

5. Current Medication

Streptomycin 1000mg, Rifampicin 600mg. 56 doses taken

6. Laboratory

BU confirmation: ZN (-) / PCR (+) for *M. ulcerans*

7. Photo documentation: Ulcer right leg, lateral

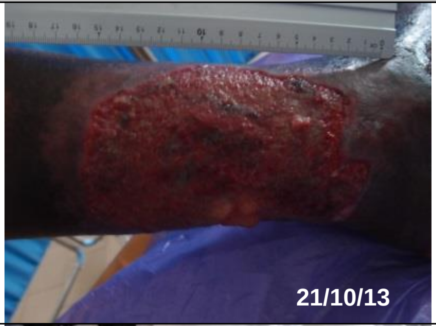

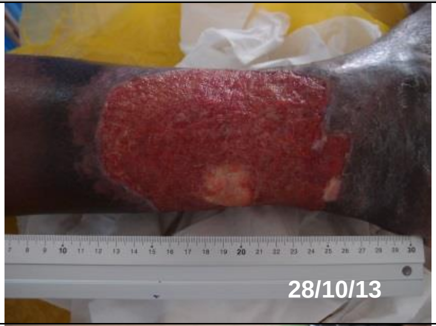

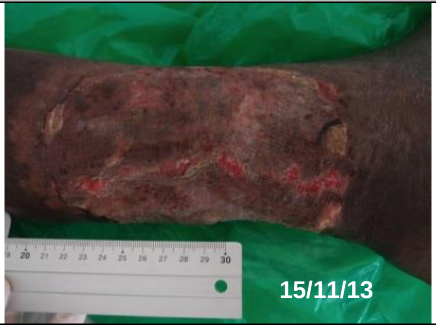

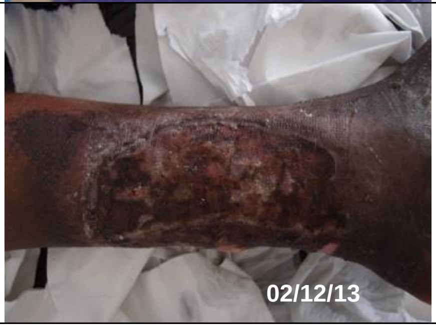

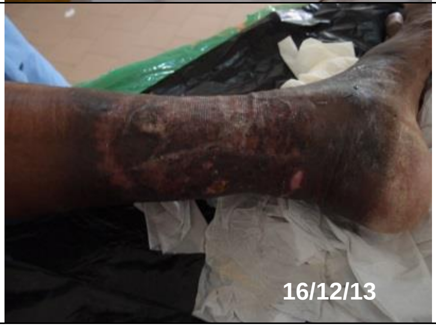

Case Report AMH

Chronic Buruli Ulcer Wounds

Patient No. 007

1. Demographic data

Sex: Male
Age: 66 years

2. Wound description

BU Category II (see Photo documentation)

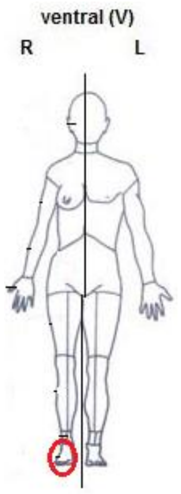

3. Medical History

<u>Wound history</u>

Wound observed since: 1 year

since 13/12/2012 hospitalized at Municipal Hospital Amasaman

05- 07/2013 Antimycobacterial treatment with Rifampicin 600mg, Streptomycin 1000mg (56 doses taken)
multiple wound excision

<u>Secondary diagnoses</u>
Hypertension

4. Physical examination

Body-Mass-Index (BMI) 22.23 kg KG/ m²

All systems normal

5. Current Medication

Zincofeer
Amlodipine 10mg (1x1)

6. Laboratory

BU confirmation: ZN (-) / PCR (+) for *M. ulcerans*

7. Photo documentation	
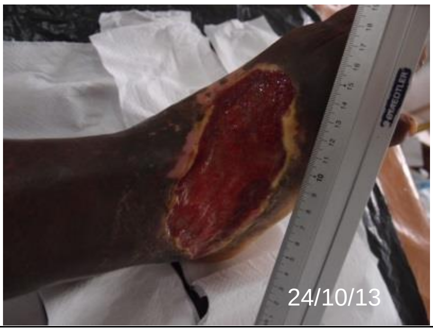	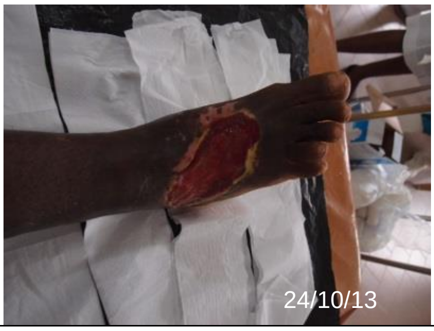

Case Report AMH

Chronic Buruli Ulcer Wounds

Patient No. 008

1. Demographic data

Sex: Male
Age: 7 years

2. Wound description

BU Category III (see Photo documentation)

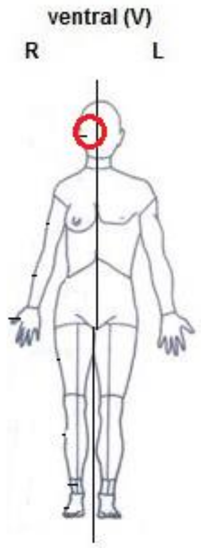

3. Medical History

Wound history

Wound observed since: 1 year

since 09/07/2013 hospitalized at Municipal Hospital Amasaman

10/07-02/09/2013 Antimycobacterial treatment with Rifampicin 300mg, Streptomycin 500mg

Secondary diagnoses

Asthma

4. Physical examination

Body-Mass-Index (BMI) 13.89 kg KG/ m²

All systems normal

5. Laboratory

BU confirmation: ZN (-) / PCR (+) for *M. ulcerans*

6. Photo documentation		
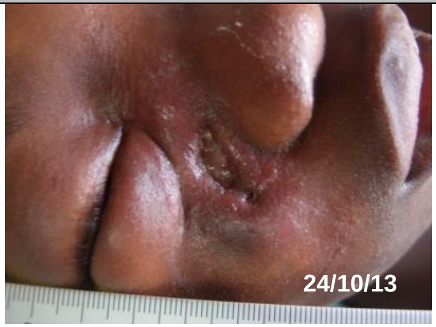	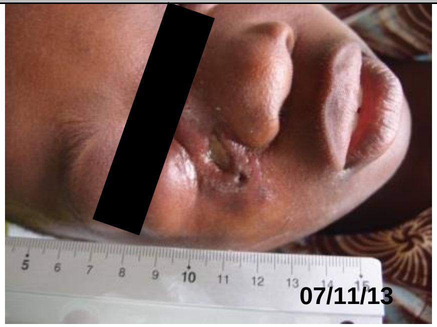	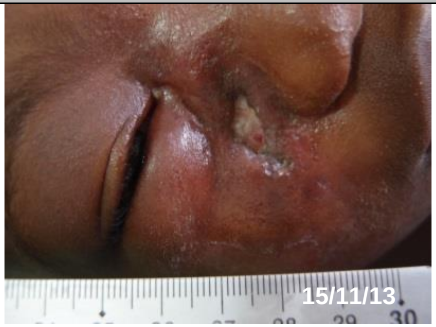
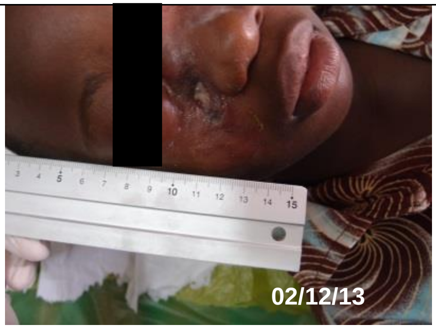	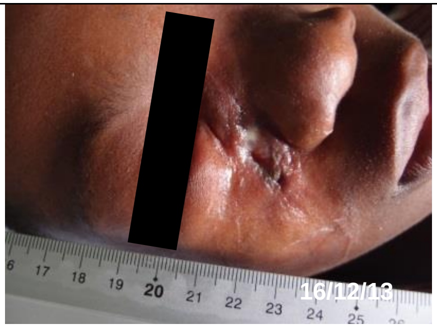	

Case Report AMH

Chronic Buruli Ulcer Wounds
Patient No. 009

1. Demographic data

Sex: Male
Age: 28 years

2. Wound description

BU Category III (see Photo documentation)

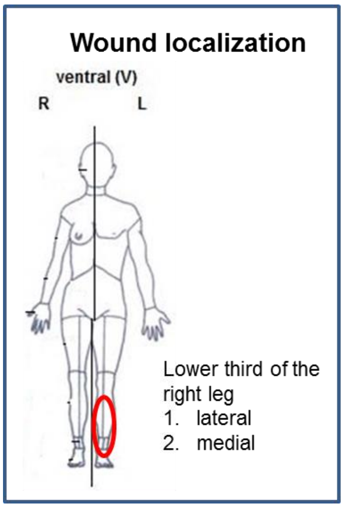

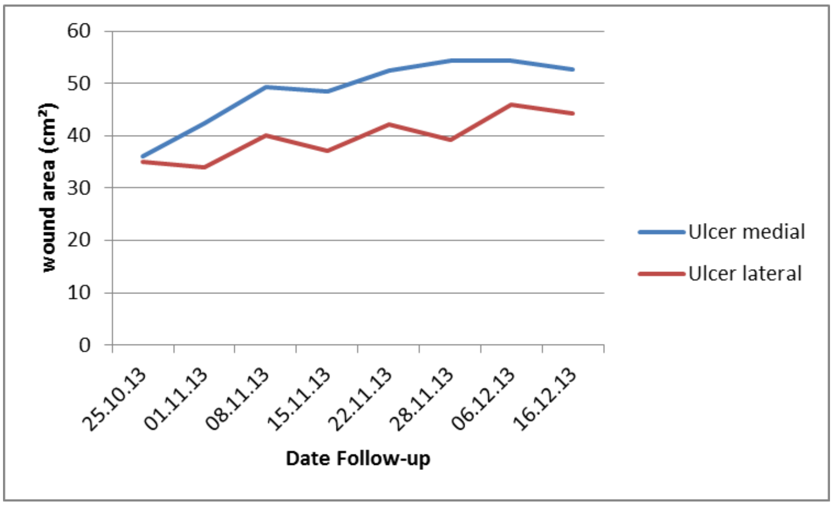

3. Medical History

<u>Wound history</u>

Wound observed since: medial: around 2004
lateral: around 2005
since 09/2009 hospitalized at Municipal Hospital Amasamam

09-11/2009	Antimycobacterial treatment with Rifampicin 600mg, Streptomycin 1000mg (56d)
01//2010	1. split skin grafting on both sides
11/2010	2. split skin grafting on both sides
07/2012	wound debridement
11/2012	3. split skin grafting on both sides
12/2013	Wound biopsy for histopathology <u>Result:</u> ulcerated skin with infiltration of the dermis by mixed inflammatory infiltrates mainly of neutrophils, lymphocytes and plasma cells. Prominent capillaries seen. No AFB's or fungal organism seen, no malignancy seen.
12/2013	discharge of hospital, attending as outpatient

<u>Secondary diagnoses</u>
Arterial hypertension (according to medical history)

4. Physical examination

Body-Mass-Index (BMI) 19,59 kg KG/ m²

All systems normal

Pain assessment: severe pain at wound and wound surrounding (7/10) especially during the night

5. Current Medication

No medication

6. Laboratory

No PCR results available

**Date**	**17.12.12**
Hb [12-16g/dl]	10,7 g/dl
HCT [37-51]	
RBC [4,2- 6,3M/ul]	
MCV [80-97fl]	
MCH [26-32 pg]	
MCHC [31-36 g/dl	

7. Microbiology

No microbiological results available

8. Photo documentation: Ulcer left leg, medial malleolus

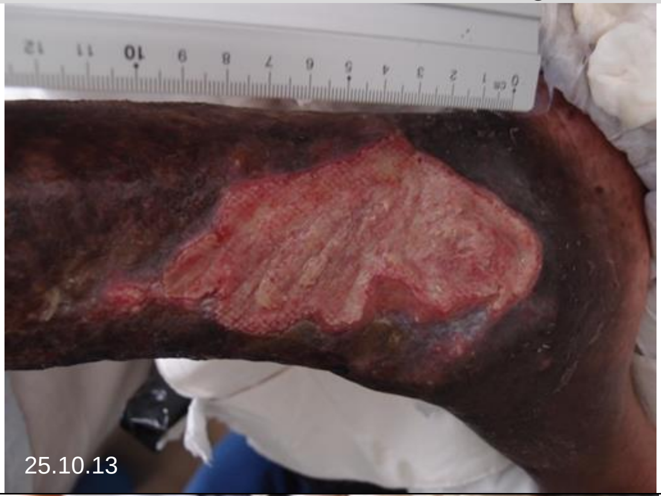
25.10.13

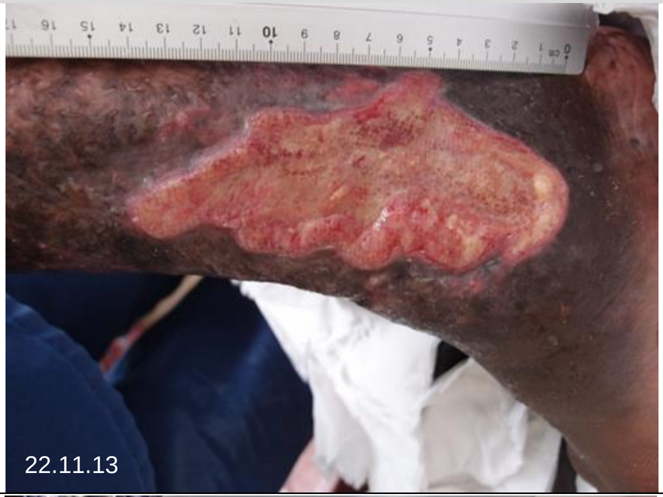
22.11.13

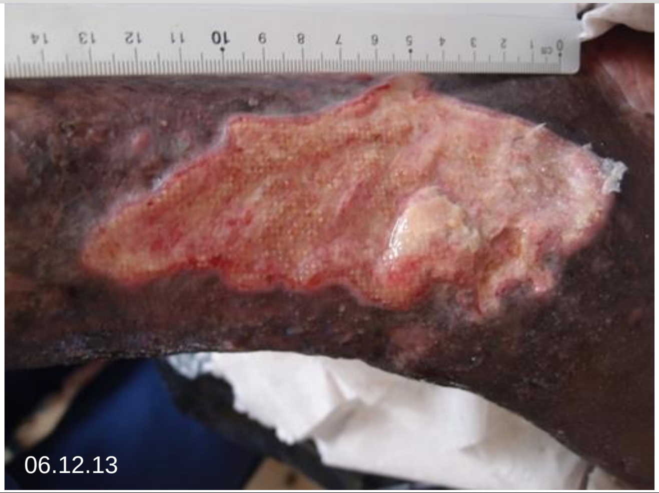
06.12.13

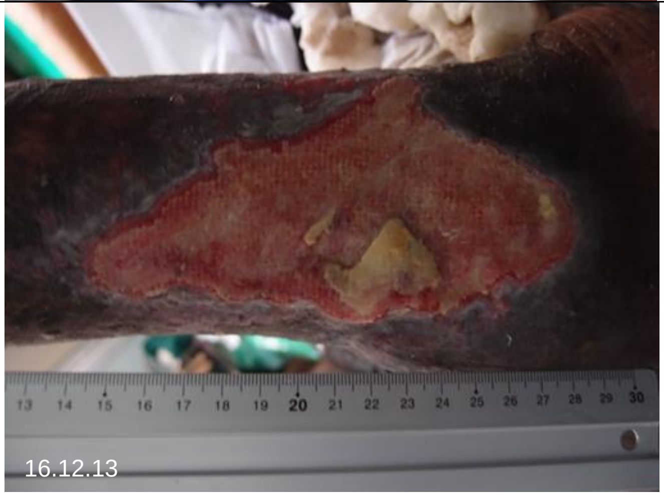
16.12.13

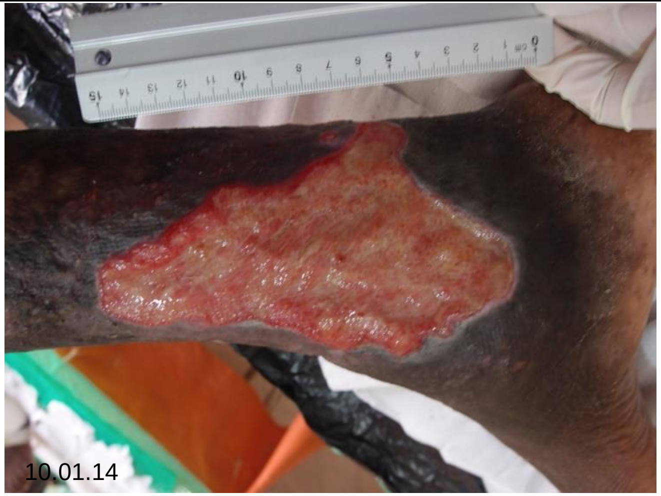
10.01.14

10.01.14

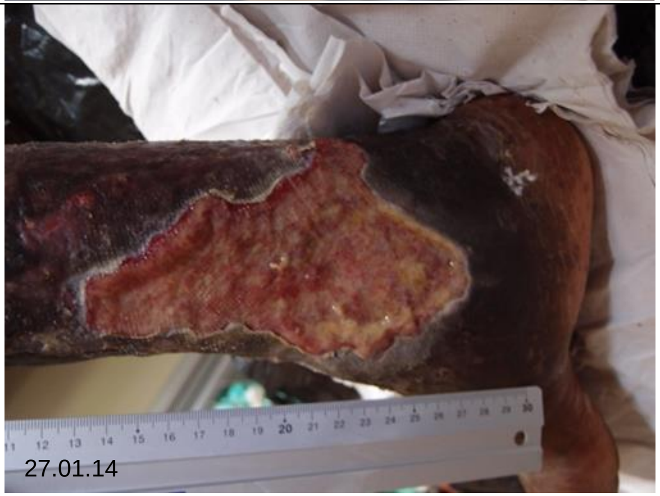
27.01.14

9. Photo documentation: Ulcer left leg, lateral malleolus

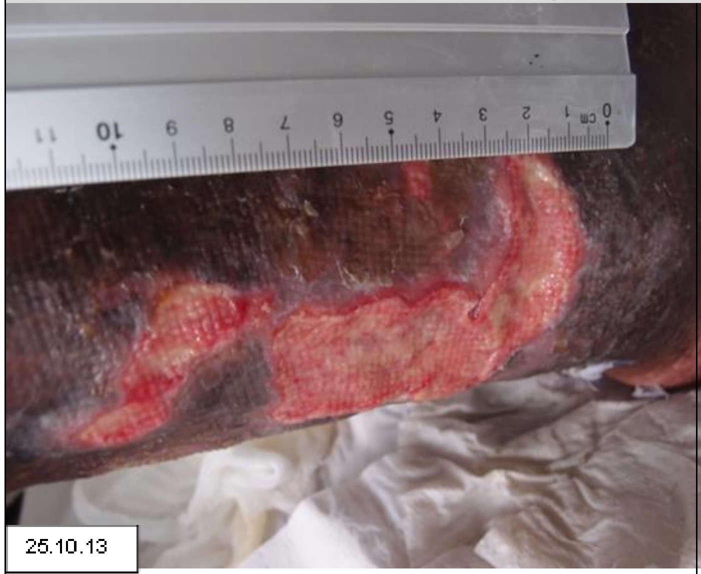

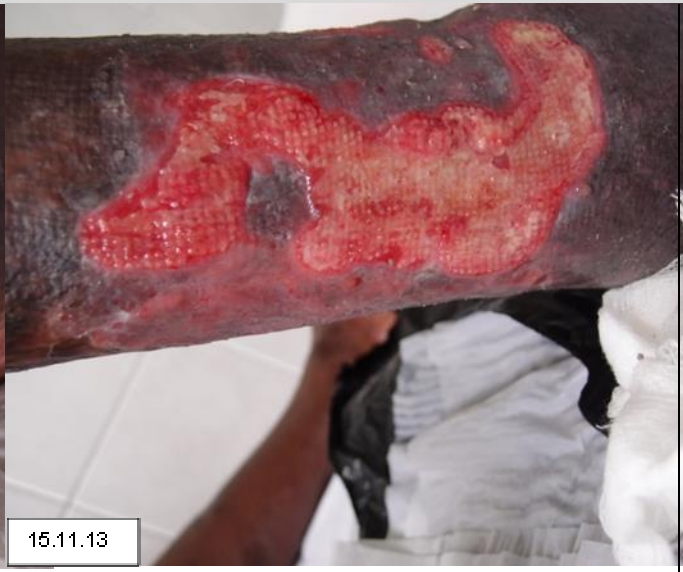

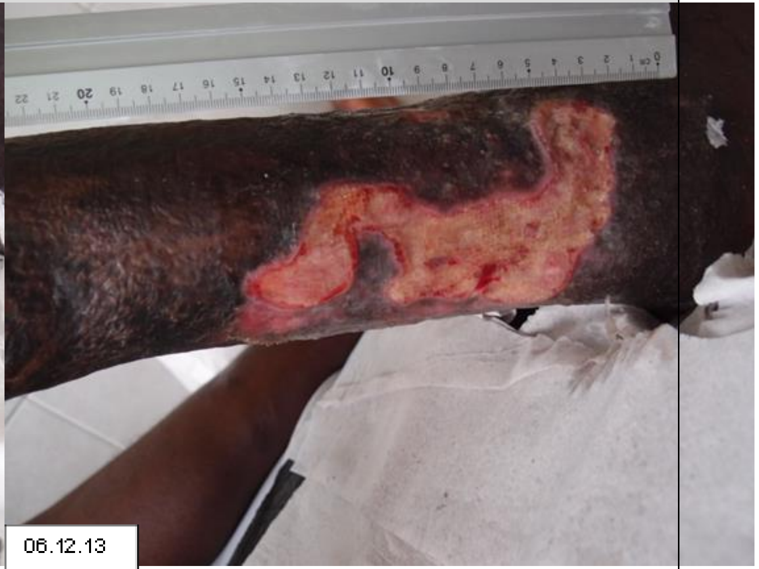

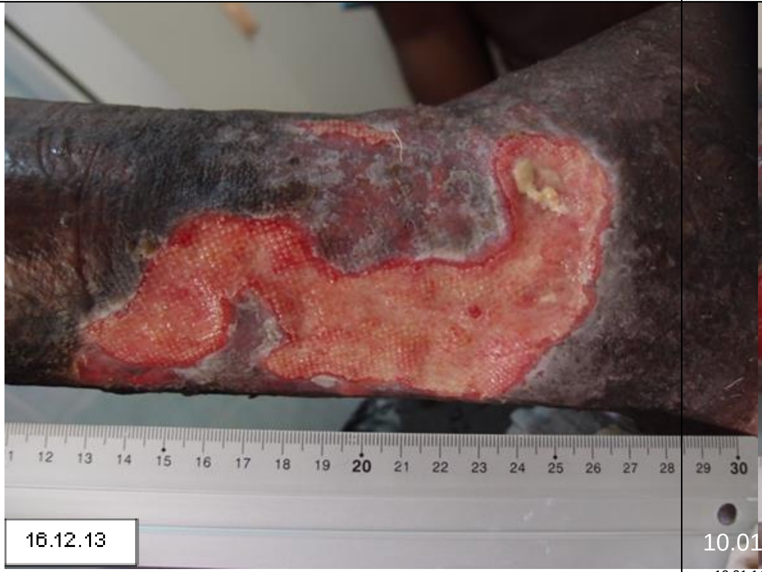

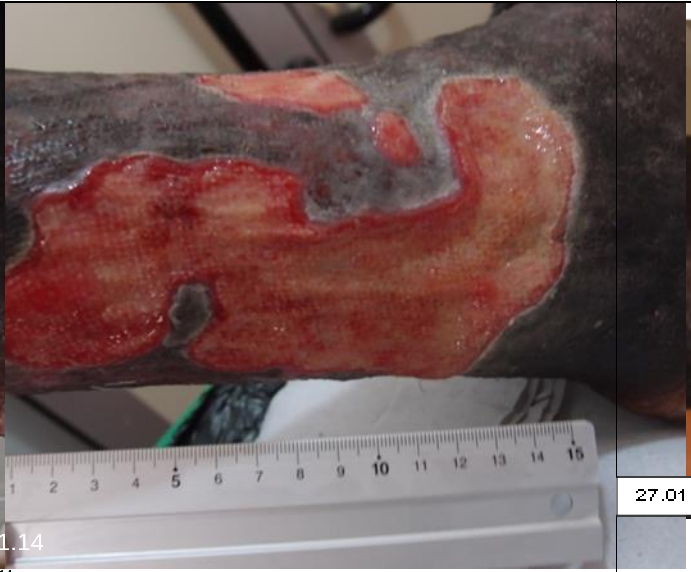

10.01.14

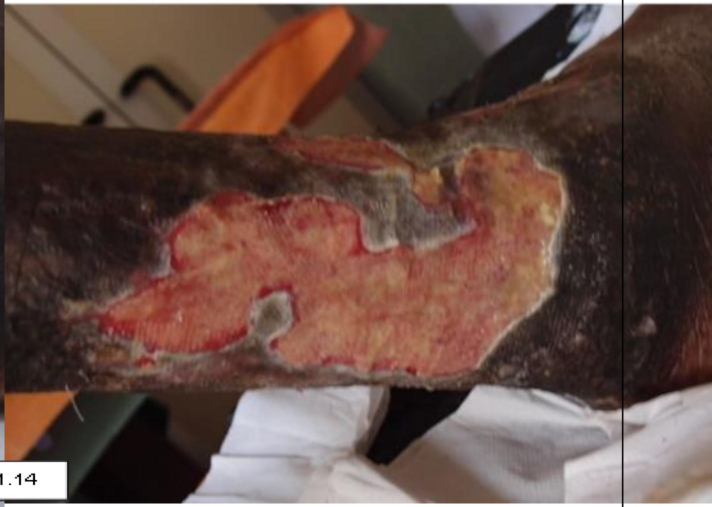

Case Report AMH

Chronic Buruli Ulcer Wounds

Patient No. 010

1. Demographic data

Sex: Male
Age: 31 years

2. Wound description

BU Category III (see Photo documentation)

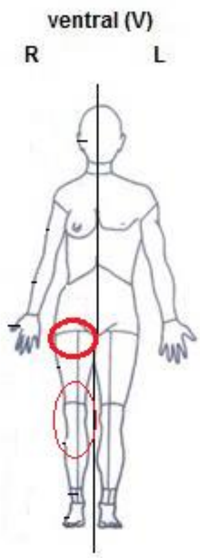

3. Medical History

Wound history

Wound observed since: 13 years

since 08/07/2013 hospitalized at Municipal Hospital Amasaman

05/2013 Amputation lower leg
Diagnosis: squamous cell carcinoma

12/2013 Patient died

Secondary diagnoses
Epilepsy

4. Physical examination

Body-Mass-Index (BMI) 19.81 kg KG/ m²

All systems normal

5. Current Medication

Streptomycin 1000mg, Rifampicin 600mg. 56 doses taken

6. Laboratory

BU confirmation: ZN(-) / PCR(+) for *M. ulcerans*

7. Photo documentation	
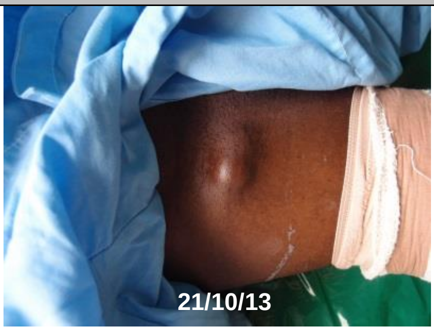	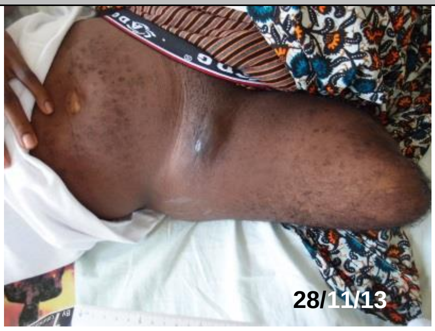
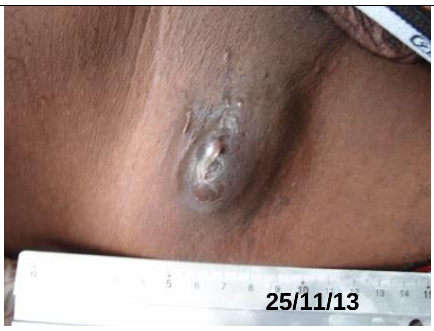	

Case Report AMH

Chronic Buruli Ulcer Wounds
Patient No. 011

1. Demographic data

Sex: Male
Age: 52 years

2. Wound description

BU Category III (see Photo documentation)

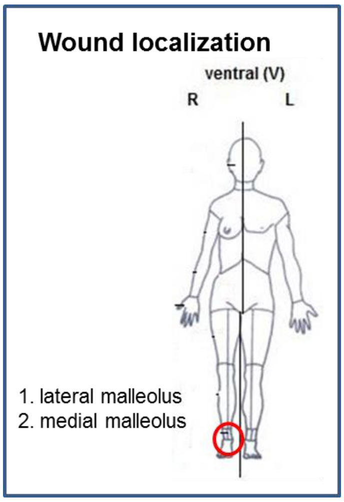

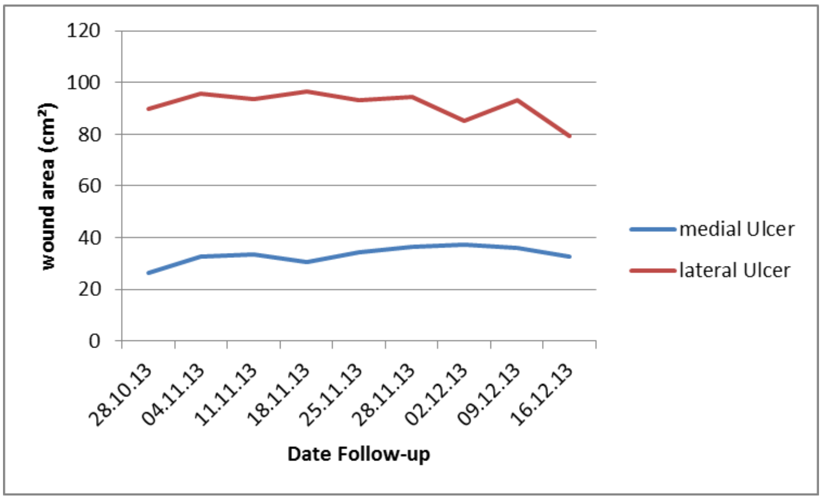

3. Medical History

<u>Wound history:</u>

Wound observed since: medial: 2008
lateral: 2009

since 05/2011 hospitalized at Municipal Hospital Amasaman

05-07/2011 Antimycobacterial treatment with Rifampicin 600 mg, Streptomycin 1000 mg (57d)
09-10/2011 Antimycobacterial treatment with Rifampicin 600 mg, Streptomycin 1000 mg (28d)
12/2011 wound excision on both sides
01/2012 biopsy ulcer for histopathology
Result: chronic non-specific ulcer
11/2012 wound debridement both sides
11/2012 split-skin grafting both sides
12/2013 biopsy ulcer for histopathology
No results available
12/2014 discharge of hospital attending as outpatient

<u>Secondary diagnoses</u>
since several years mild hear impairment
07/2011 arterial hypertension
11/2013 Gastritis, *Heliobacter pylori* triple therapy

4. Physical examination

Body-Mass-Index (BMI) 27,13 kg KG/ m²

All systems normal

Pain assessment: severe pain at wound and wound surrounding (7-8/10) especially in the evening and during the night

5. Current Medication

Zinc and Vitamin supplements
Bendroflumethiazide 2,5 mg (1-0-0)

6. Laboratory

BU confirmation: 05/2011 ZN+/ PCR- for *M. ulcerans*

**Date**	**30.05.12**
Hb [12-16g/dl]	8,4 g/dl

7. Microbiology

Results of wound swabs (2011-2013)

Proteus mirabilis: sensitive to Cefuroxime
Koagulase neg. staphylococii: sensitive to Amikacin

8. Photo documentation: Ulcer right leg, lateral malleolus

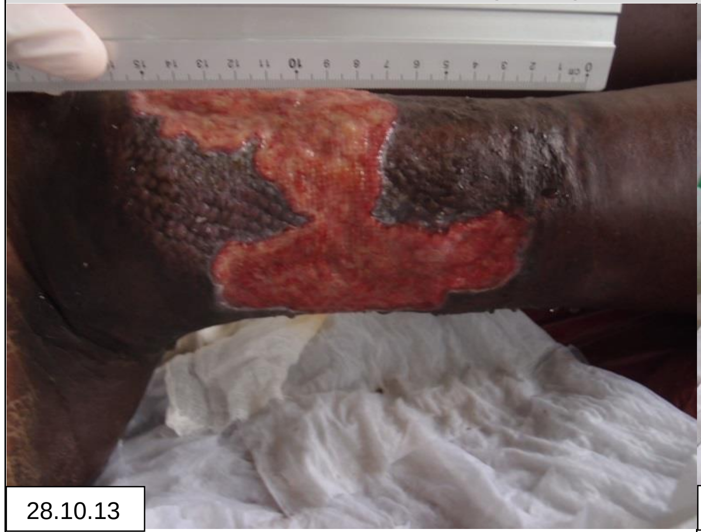

28.10.13

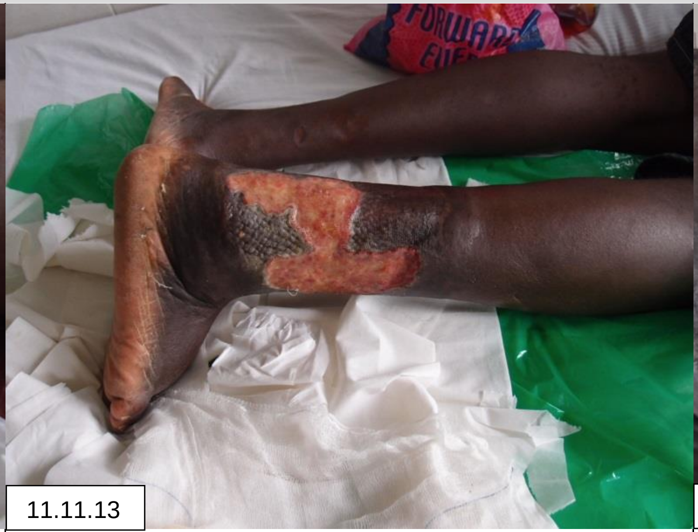

11.11.13

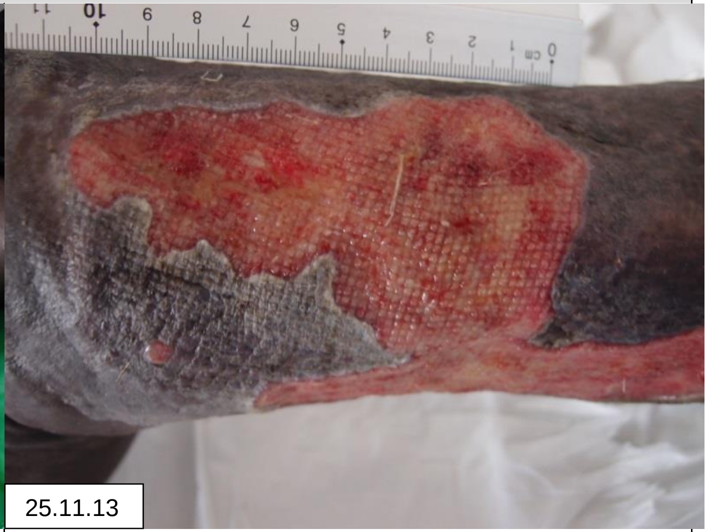

25.11.13

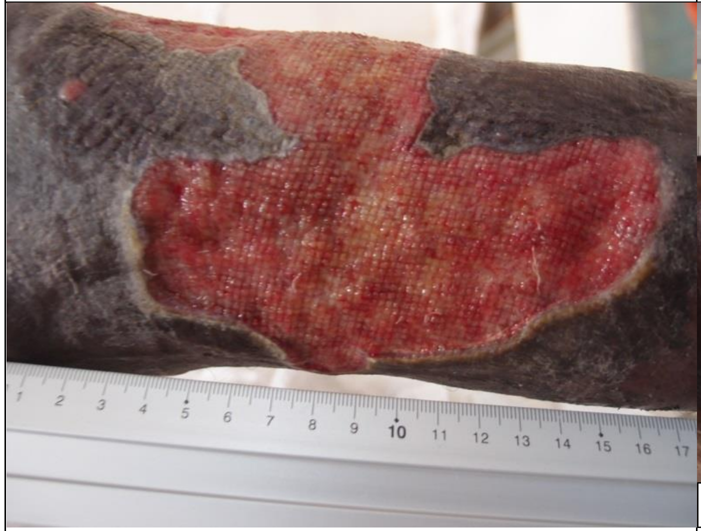

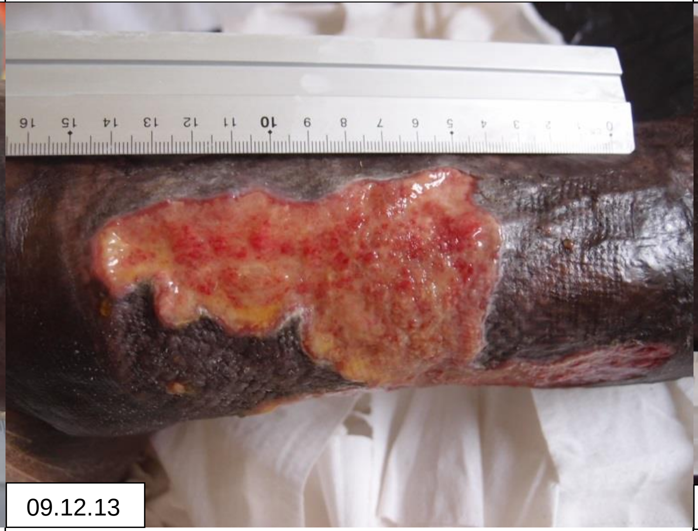

09.12.13

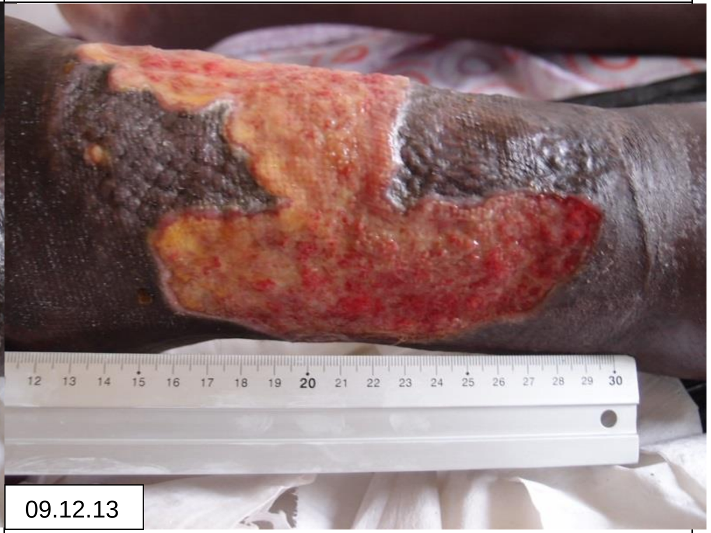

09.12.13

9. Photo documentation: Ulcer right leg, medial malleolus

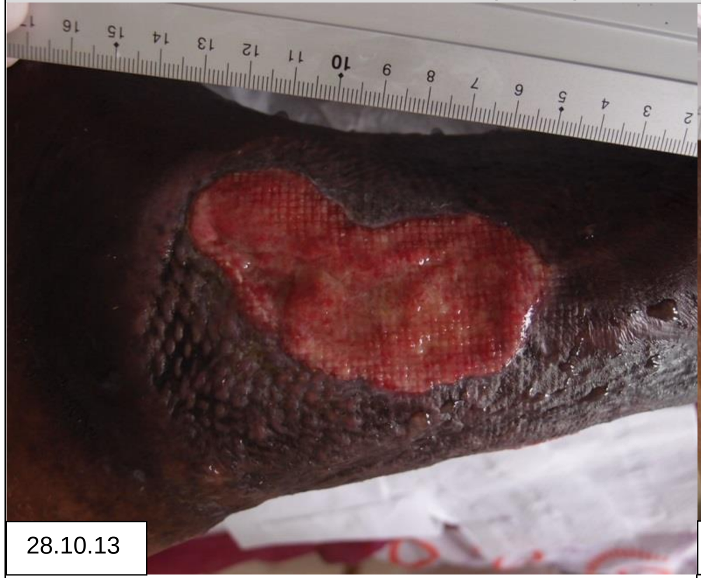

28.10.13

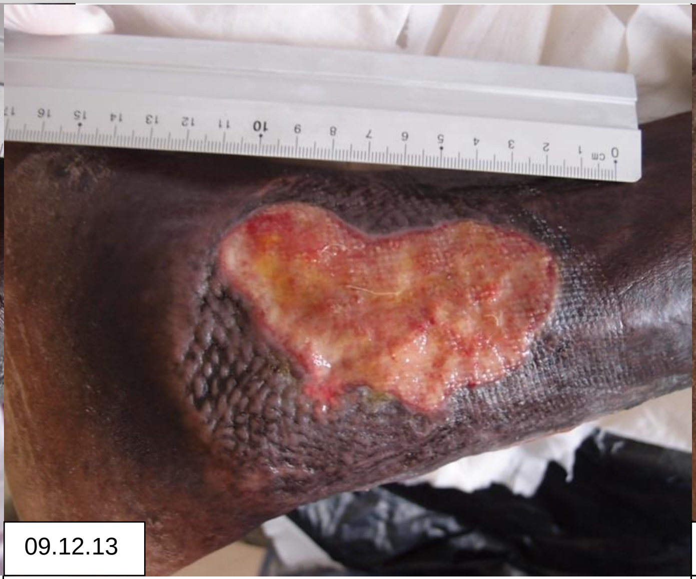

09.12.13

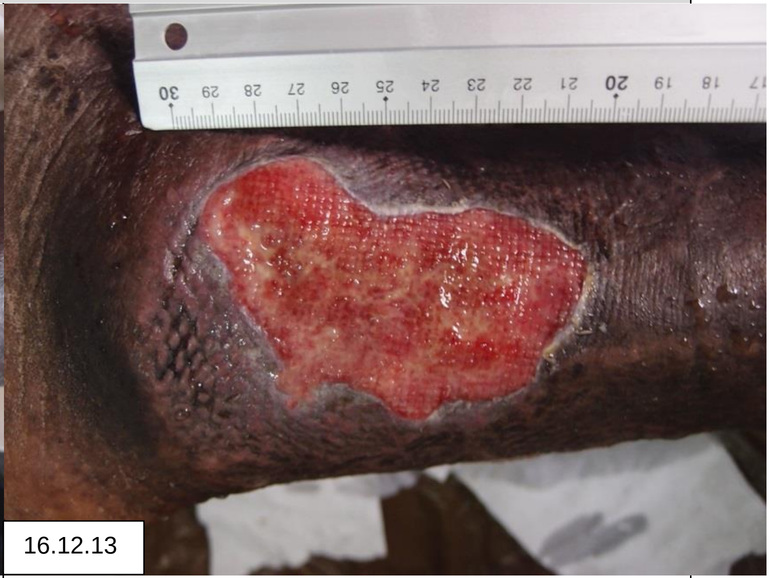

16.12.13

Case Report AMH

Healed Buruli Ulcer Wounds

Patient No. 012

1. Demographic data

Sex: Male
Age: 27 years

2. Wound description

BU Category III (see Photo documentation)

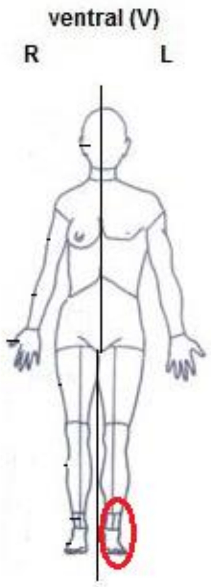

3. Medical History

Nil of significance

Wound history

Wound observed since: 10 years

since 27/08/2013 hospitalized at Municipal Hospital Amasaman

08/2013 Split- skin grafting

4. Physical examination

Body-Mass-Index (BMI) 18.51kg KG/ m²

All systems normal

5. Current Medication

Streptomycin 1000mg, Rifampicin 600mg. 56 doses taken

6. Laboratory

BU confirmation: ZN(-) / PCR(+) for *M. ulcerans*

7. Photo documentation

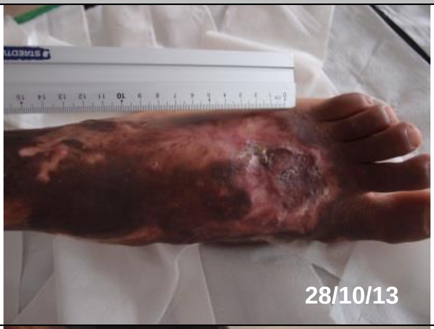

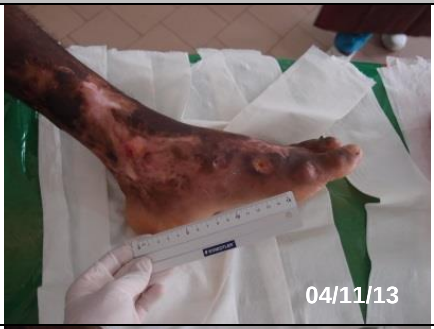

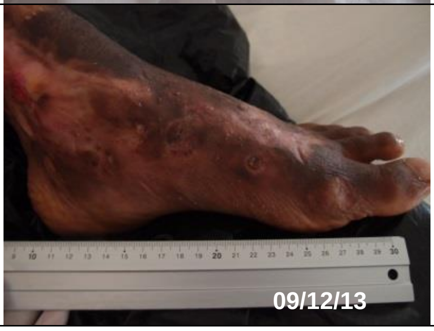

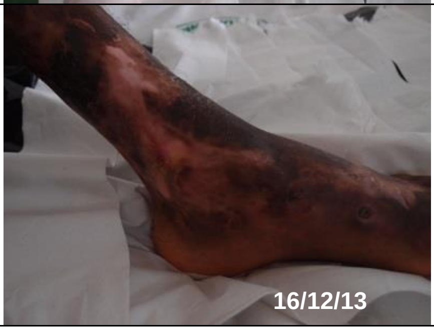

Case Report AMH

Chronic Buruli Ulcer Wounds

Patient No. 013

1. Demographic data

Sex: Male
Age: 19 years

2. Wound description

BU Category III (see Photo documentation)

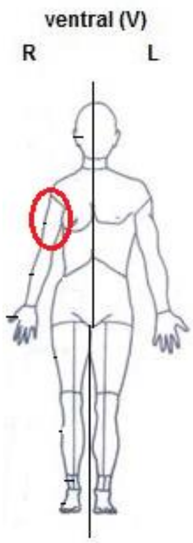

14.11.13
X- ray;

Diagnosis:
chronic
osteomyelitis

3. Medical History

Nil of signifcance

<u>Wound history</u>

Wound observed since: 2011

since 02/09/2013 hospitalized at Municipal Hospital Amasaman

08-10/2013 Antimycobacterial treatment with Rifampicin 600mg, Streptomycin 1000mg

11/2013 Chronic osteomyelitis

Patient referred to tertiary care hospital

4. Physical examination

Body-Mass-Index (BMI) 16.79 kg KG/ m²

All systems normal

5. Laboratory

BU confirmation: ZN(+) / PCR(+) for *M. ulcerans*

6. Photo documentation

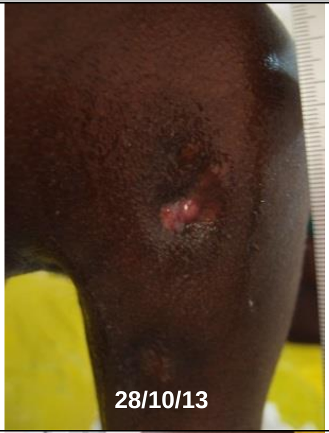

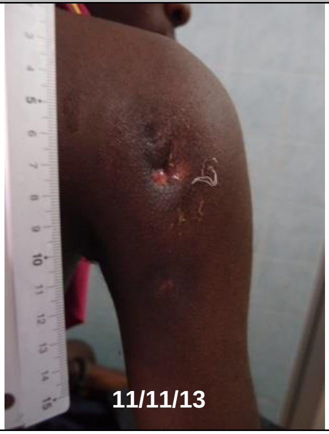

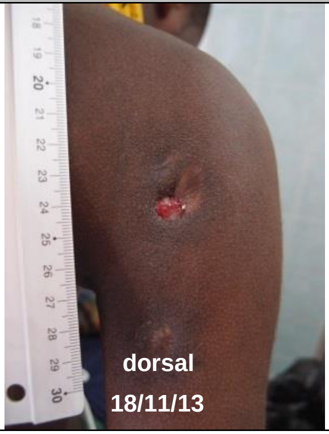

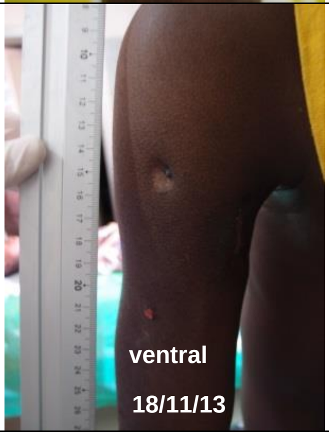

Case Report AMH

Healed Buruli Ulcer Wounds
Patient No. 014

1. Demographic data

Sex: Male
Age: 8 years

2. Wound description

BU Category III (see Photo documentation)

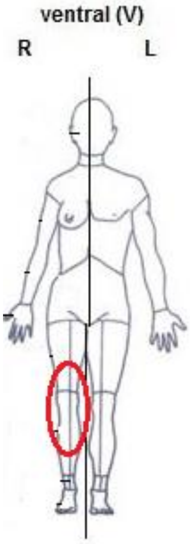

3. Medical History

Nil of significance

<u>Wound history</u>

Wound observed since: 2010

since 07/02/2013 hospitalized at Municipal Hospital Amasaman

Complication: knee joint contraction

4. Physical examination

Body-Mass-Index (BMI) 14,72 kg KG/ m²

All systems normal

5. Current Medication

Streptomycin 500mg, Rifampicin 300mg. 56 doses taken

6. Laboratory

BU confirmation: ZN (-) / PCR (+) for *M. ulcerans*

7. Photo documentation	
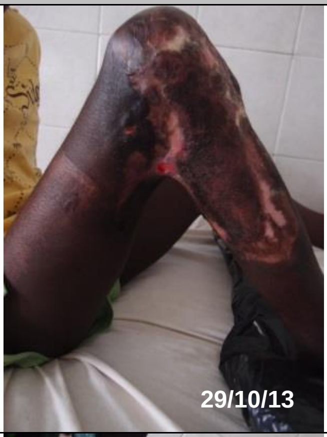	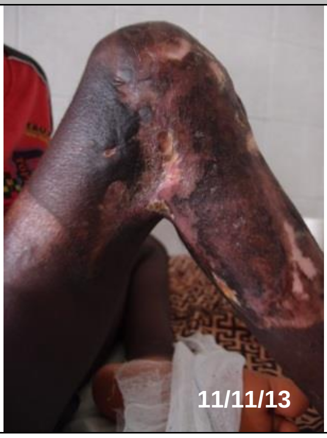
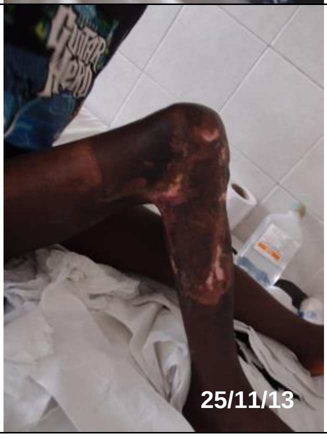	

Case Report AMH

Chronic Buruli Ulcer Wounds
Patient No. 015

1. Demographic data

Sex: Male
Age: 8 years

2. Wound description

BU Category III (see Photo documentation)

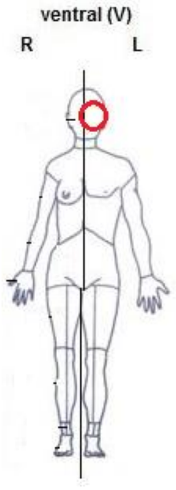

3. Medical History

Nil of significance

<u>Wound history</u>

Wound observed since: 2011

Since 15/05/2103 hospitalized at Municipal Hospital Amasaman

05-07/2013 Antimycobacterial treatment with Rifampicin 300mg, Streptomycin 500mg (56d)

12/2013 diagnosed bone necrosis, referred to hospital of tertiary health care

4. Physical examination

Body-Mass-Index (BMI) 13.02 kg KG/ m²

All systems normal

5. Current Medication

Folic acid
Multivitamin

6. Laboratory

BU confirmation: ZN(+) / PCR(+) for *M. ulcerans*

1. Photo documentation

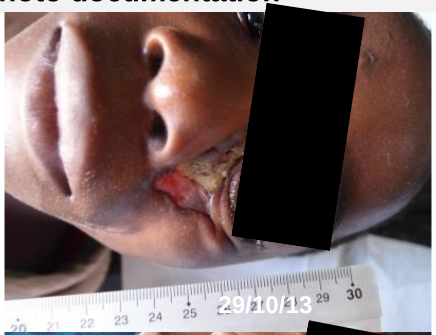

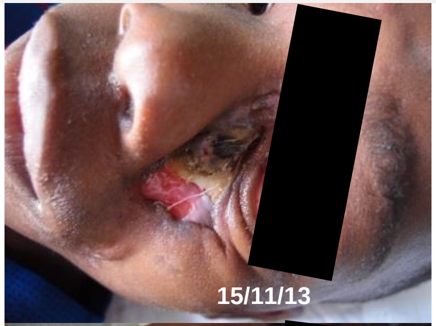

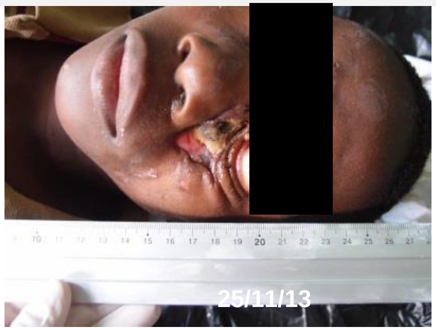

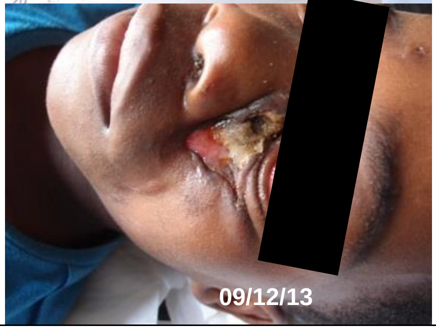

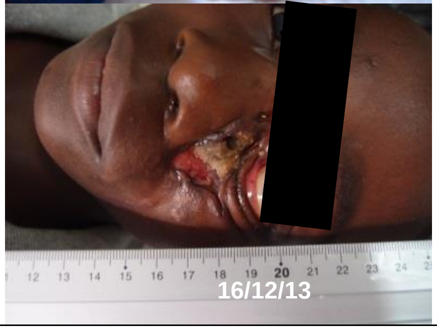

Case Report AMH

Healed Buruli Ulcer Wounds
Patient No. 016

1. Demographic data

Sex: Female
Age: 25 years

2. Wound description

BU Category I (see Photo documentation)

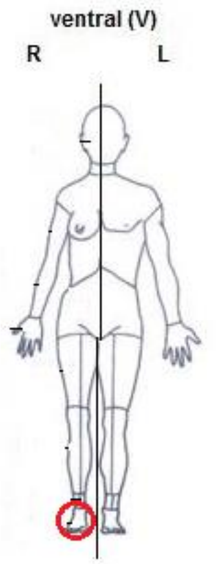

3. Medical History

Nil of significance

<u>Wound history</u>

Wound observed since:10/2012

since 17/07/2013 hospitalized at Municipal Hospital Amasaman

08/11/2013 wound excision

12/2013 split skin grafting

01/ 2014 wound helaed

4. Physical examination

Body-Mass-Index (BMI) 23.01 kg KG/ m²

All systems normal

5. Current Medication

Streptomycin 1000mg, Rifampicin 600mg. 56 doses taken

6. Laboratory

BU confirmation: ZN(+) / PCR(+) for *M. ulcerans*

7. Photo documentation

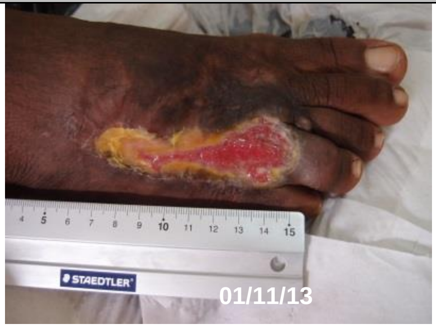

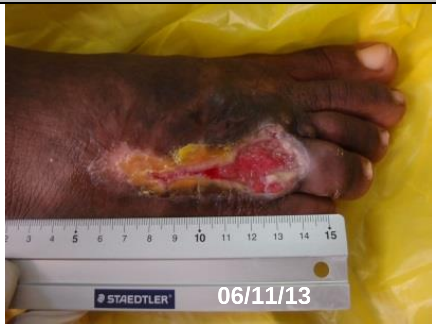

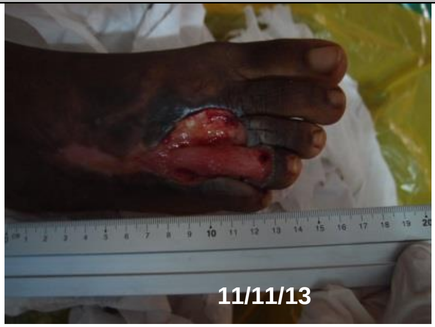

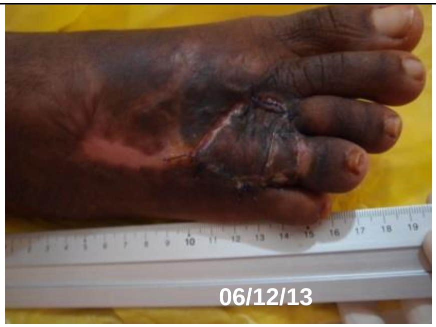

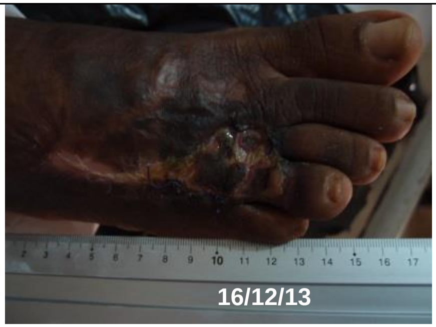

Case Report AMH

Chronic Buruli Ulcer Wounds

Patient No. 017

1. Demographic data

Sex: Female
Age: 73 years

2. Wound description

BU Category III (see Photo documentation)

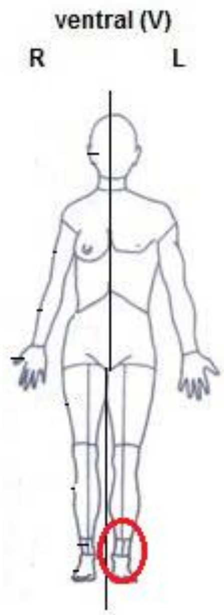

3. Medical History

<u>Wound history</u>

Wound observed since: 3 years

since 28/08/2013 hospitalized at Municipal Hospital Amasaman

29/08- 23/10/2013 Antimycobacterial treatment with Rifampicin 600mg, Streptomycin 1000mg (56d)

<u>Secondary diagnoses</u>
Hypertension

4. Physical examination

All systems normal

5. Current Medication

Zinkofer

6. Laboratory

BU confirmation: ZN (-) / PCR (+) for *M. ulcerans*

7. Photo documentation

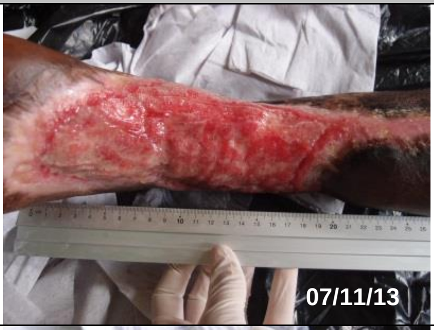

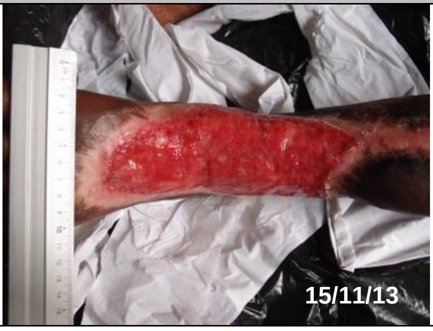

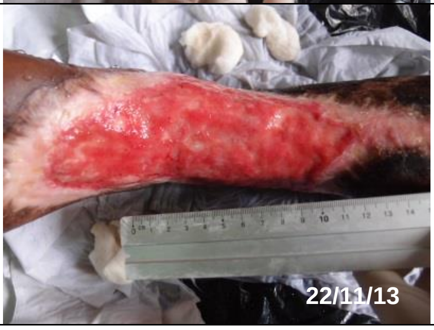

Case Report AMH

Healed Buruli Ulcer Wounds
Patient No. 018

1. Demographic data

Sex: Female
Age: 72 years

2. Wound description

BU Category I (see Photo documentation)

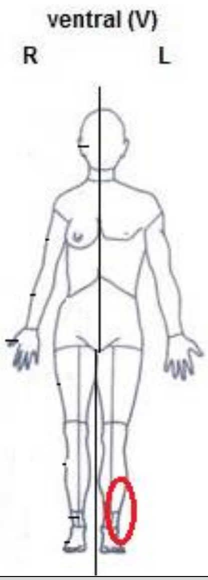

3. Medical History

<u>Wound history</u>

Wound observed since: 8 months

since 06/11/2013 treated as outpatient at Municipal Hospital Amasaman

11-12/2013 Antimycobacterial treatment with Rifampicin 600mg, Streptomycin 1000mg (56d)

01/2014 wound excision

<u>Secondary diagnoses</u>
Asthma

4. Physical examination

Body-Mass-Index (BMI) 42.06 kg KG/ m^2

All systems normal

5. Current Medication

Amlodipine 5mg (1x1), Vitamin B complex
Salbutamol, Prednisolon 5mg

6. Laboratory

BU confirmation: ZN(+) / PCR(+) for *M. ulcerans*

7. Photo documentation

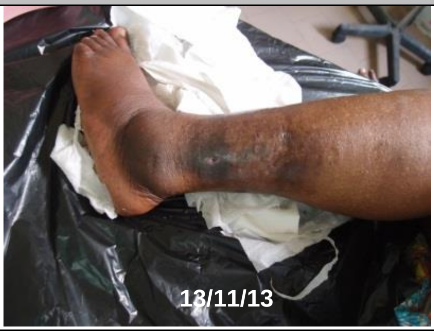

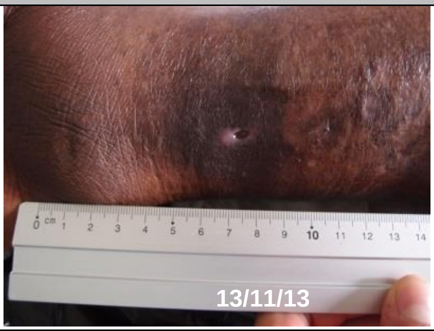

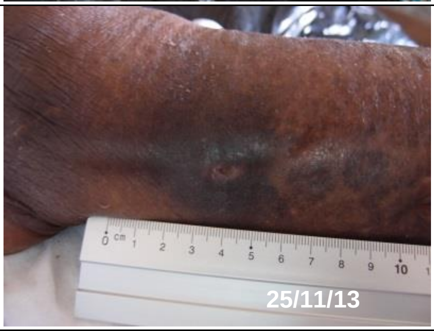

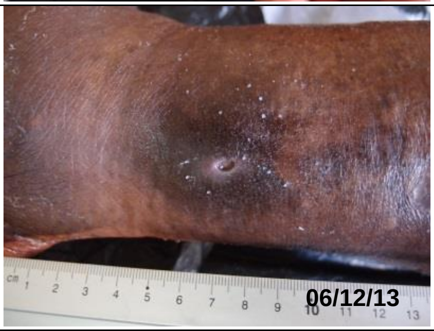

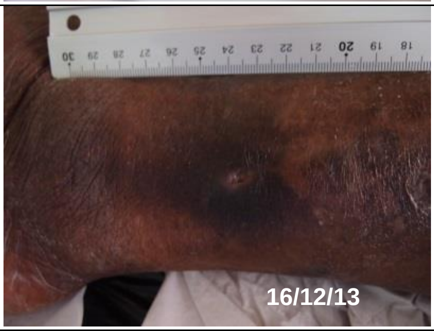

Case Report AMH

Chronic Buruli Ulcer Wounds
Patient No. 019

1. Demographic data

Sex: Female
Age: 45 years

2. Wound description

BU Category III (see Photo documentation)

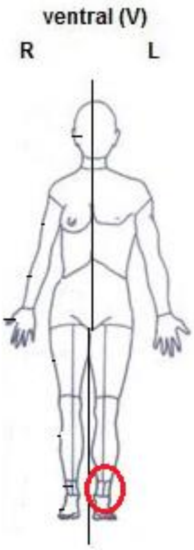

3. Medical History

Nil of significance

<u>Wound history</u>

Wound observed since: 2010

since 25/11/2013 hospitalized at Municipal Hospital Amasaman

11-12/2013 Antimycobacterial treatment with Rifampicin 600mg, Streptomycin 1000mg (56d)

4. Physical examination

All systems normal

5. Current Medication

Vitamin B complex

6. Laboratory

BU confirmation: ZN (-) / PCR (+) for *M. ulcerans*

7. Laboratory

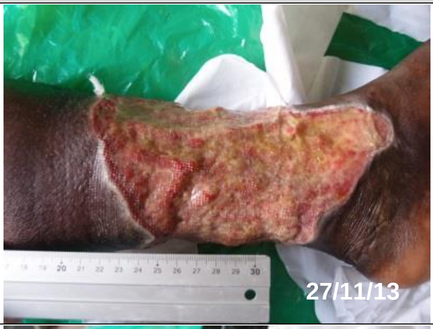

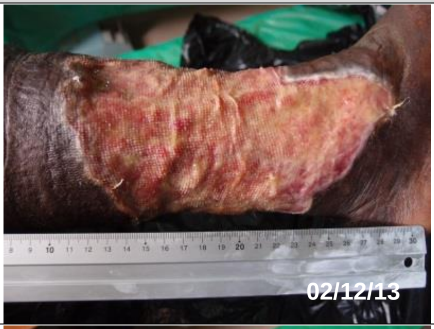

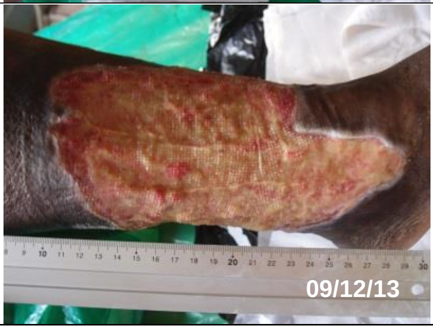

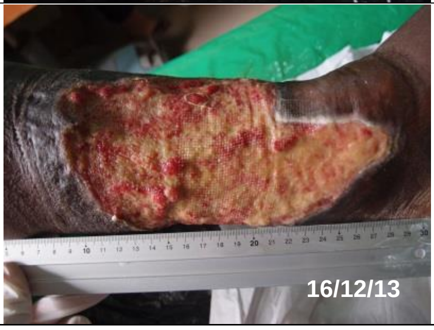

Case Report AMH

Chronic Buruli Ulcer Wounds
Patient No. 020

1. Demographic data

Sex: Male
Age: 69 years

2. Wound description

BU Category III (see Photo documentation)

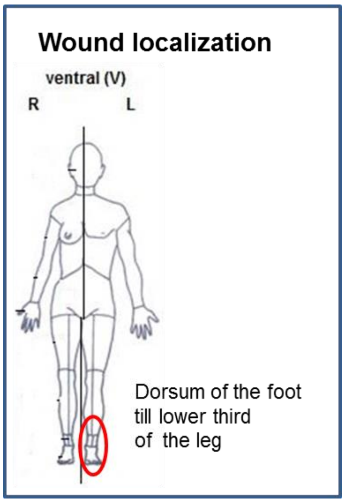

3. Medical History

<u>Wound History</u>

Wound observed since: 07/ 2013

since 09/2013	consultation as an outpatient at the Municipal Hospital Amasaman
10-11/2013	Antimycobacterial treatment with Rifampicin 600 mg, Streptomycin 1000 mg (56d)

<u>Secondary diagnoses</u>
No further diseases known

4. Physical examination

Cardiovascular system: normal, no signs of varicose

All systems normal

Pain assessment: Severe pain at wound in the left leg (7-8/10), unable to walk

5. Current Medication

Diclofenac

6. Laboratory

BU confirmation:
09/2013 ZN- / PCR- for *M. ulcerans*
10/2013 ZN-/ PCR+ for *M .ulcerans*

7. Microbiology

Results of wound swabs

09/2013 mixed culture report (pus cells, G+ rods, G- cocci. G- rods), suspected to be infected
11/2013 *Pseudomonas fluorescence/ putida* (Interpretation of bacterial load: infected)
01/2014 presence of numerous maggots in the wound

8. Photo documentation: Ulcer left leg, lateral malleolus

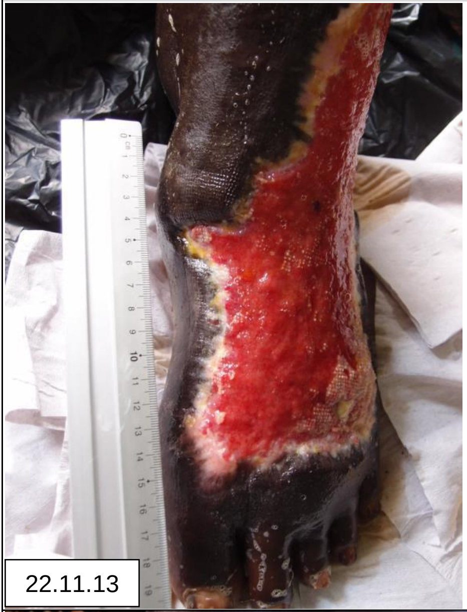

22.11.13

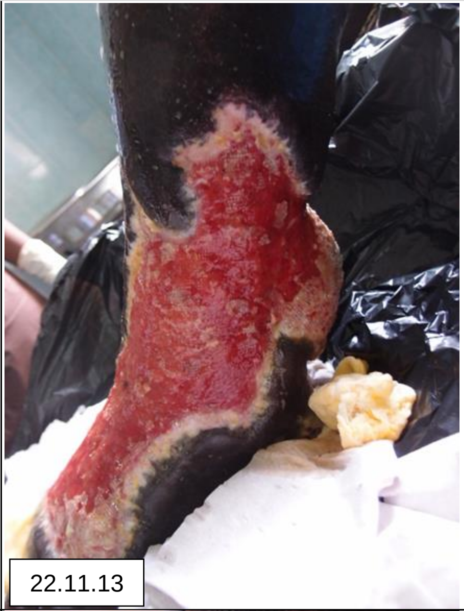

22.11.13

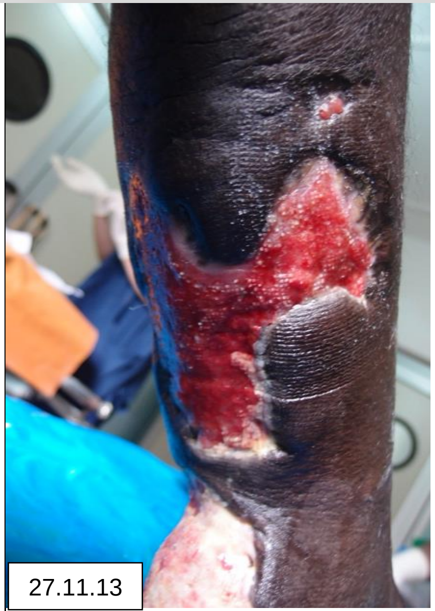

27.11.13

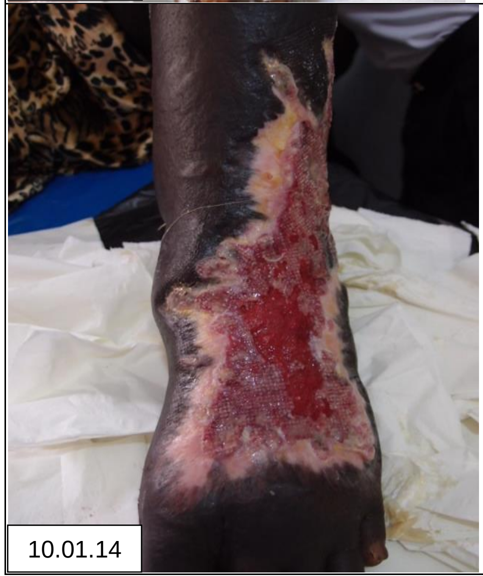

10.01.14

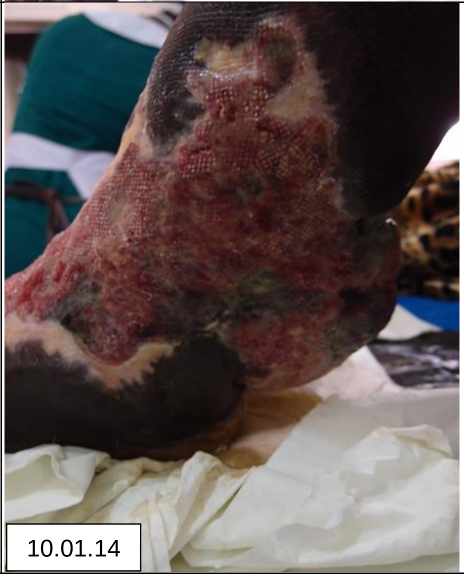

10.01.14

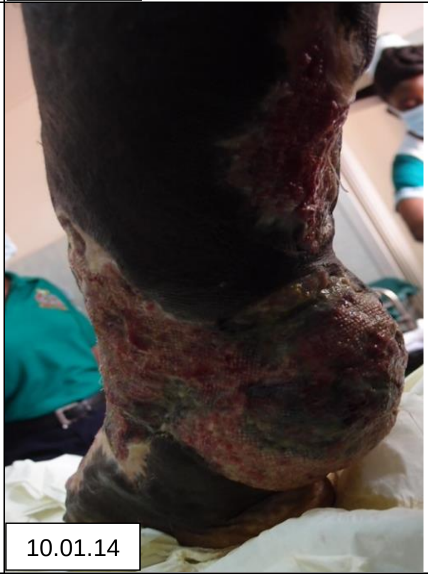

10.01.14

Case Report AMH

Chronic Buruli Ulcer Wounds

Patient No. 021

1. Demographic data

Sex: Male
Age: 10 years

2. Wound description

BU Category I (see Photo documentation)

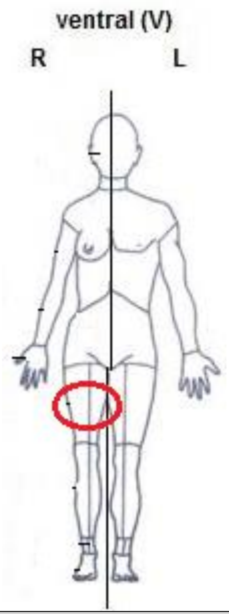

3. Medical History

Nil of significance

<u>Wound history</u>

Wound observed since: 2 years

since 22/11/2013 hospitalized at Municipal Hospital Amasaman

4. Physical examination

Body-Mass-Index (BMI) 13.02 kg KG/ m²

All systems normal

5. Current Medication

Streptomycin 500mg, Rifampicin 300mg. 56 doses taken

6. Laboratory

BU confirmation: ZN(+) / PCR(+) for *M. ulcerans*

7. Photo documentation		
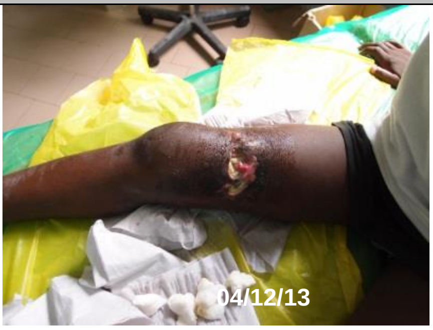	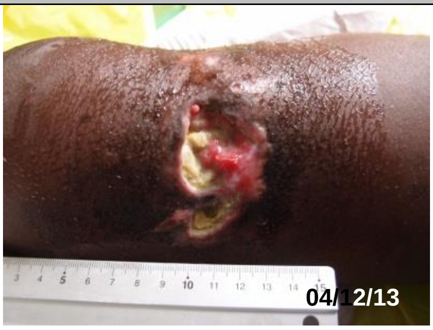	

Case Report AMH

Chronic Buruli Ulcer Wounds

Patient No. 023

1. Demographic data

Sex: Female
Age: 22 years

2. Wound description

BU Category III (see Photo documentation)

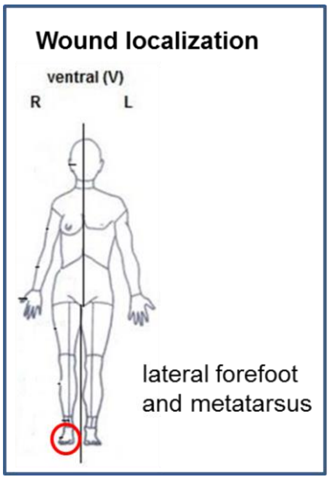

3. Medical History

<u>Wound history</u>

repeated wounds at the same localization:
2001-2004 (healed)
01/2010- 03/2011 (healed)

current wound observed since: 02/2013

since 01/2011	consultation at the health post in Obom as an outpatient
01-03/2011	Antimycobacterial treatment with Rifampicin 600 mg, Streptomycin 1000 mg (58d)
since 02/2014	hospitalized at Municipal Hospital Amasaman
02/2014	wound biopsy for histopathology Result: well differentiated invasive squamous cell carcinoma
02/2014	chest X-ray: "lung fields looks normal" X-ray of foot: 3.5 digits and the metatarsals not present

4. Physical examination

Body-Mass-Index (BMI) 16,73 kg KG/ m²

Cardiovascular system: normal, no signs of varicose

Respiratory system: normal, vesicular sound

Abdomen: soft, liver palpable with 2 cm below costal margin, no spleen palpable

Pain assessment: Constant, severe pain at wound and wound surrounding (7-9/10)

Lymph nodes: right and left inguinal lymph nodes palpable, not painful

Skin/ sclera: skin is very pale and the sclera is affected by jaundice

5. Current Medication

Diclofenac 50 mg tid
Oral Tramadaol 50 mg tid
Oral Fersolate 2x1
Oral Vitamin C 100mg 2x1

6. Laboratory

01/2011 ZN+/ PCR- for *M. ulcerans*

**Date**	**18.12.2013**	**12.02.2014**
Hb [12-16g/dl]	6,1 g/dl	5,5 g/dl
HCT [37-51]	22,3%	17,9%
RBC [4,2- 6,3M/ul]	2,87 M/ul	2,61 M/ul
MCV [80-97fl]	77,7fl	68.6 fl
MCH [26-32 pg]	21,3pg	21,1 pg
MCHC [31-36 g/dl	27,4 g/dl	30,7 g/dl
WBC [4.1-10.9 K/ul]<br>- Neutrophils [25-75%]<br>- Lymphocytes[20-60%]<br>- Monocytes [2-10%]<br>- Eosinophils [1-6%]<br>- Basophils [0-1%]	11,9 K/ul<br>69,0%<br>21,4%	12,8 K/ul<br>64,5%<br>34,4%
PLT [140-440 K/ul]	628K/ul	673 K/ul
Urea [2,1- 7,1 mmol/l]	1,2 mmol/l	
Creatinine [53- 106 umol/l]	72,7 umol/l	
Albumin [35-52 g/l]	26,7 g/dl	
T.Protein [60-78 g/l]	60,3g/dl	
T.Bilirubin [5-21 umol/l]	7,0 umol/l	
D.Bilirubin [<3,4 umol/l]	6,8 umol/l	
AST [<40 U/l]	4,5 U/l	
ALT [<40 U/l]	64,2 U/l	
Gamma Gt [6-24 U/l]	85,2 U/l	
ALKP [<240 U/l]	610,4 U/l	

7. Microbiology

Results of wound swabs

30.10.2013 *Proteus mirabilis*: sensitive to Amikacin, Cefuroxime, Ceftriaxone, Ampicillin,

10. Photo documentation: Ulcer right foot

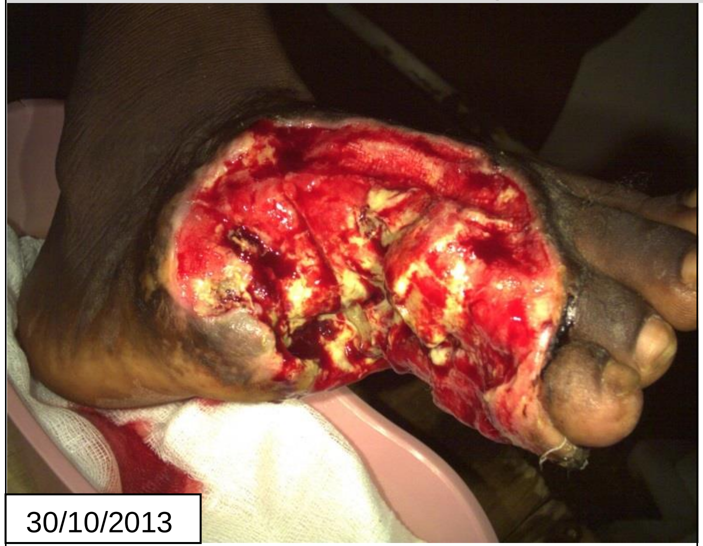

30/10/2013

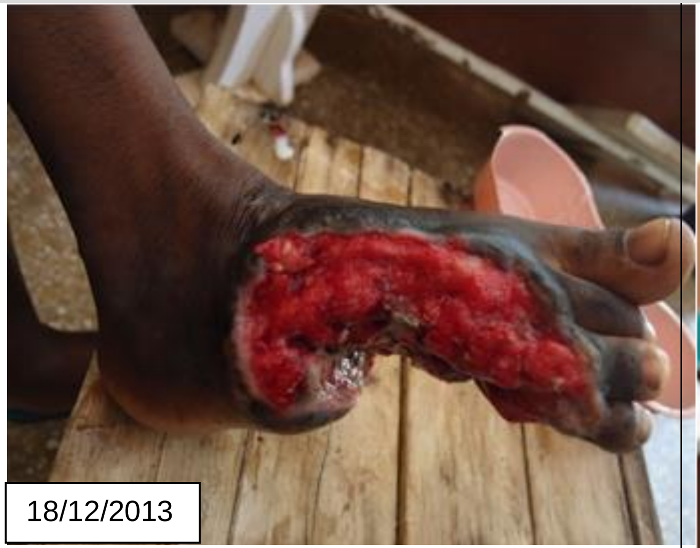

18/12/2013

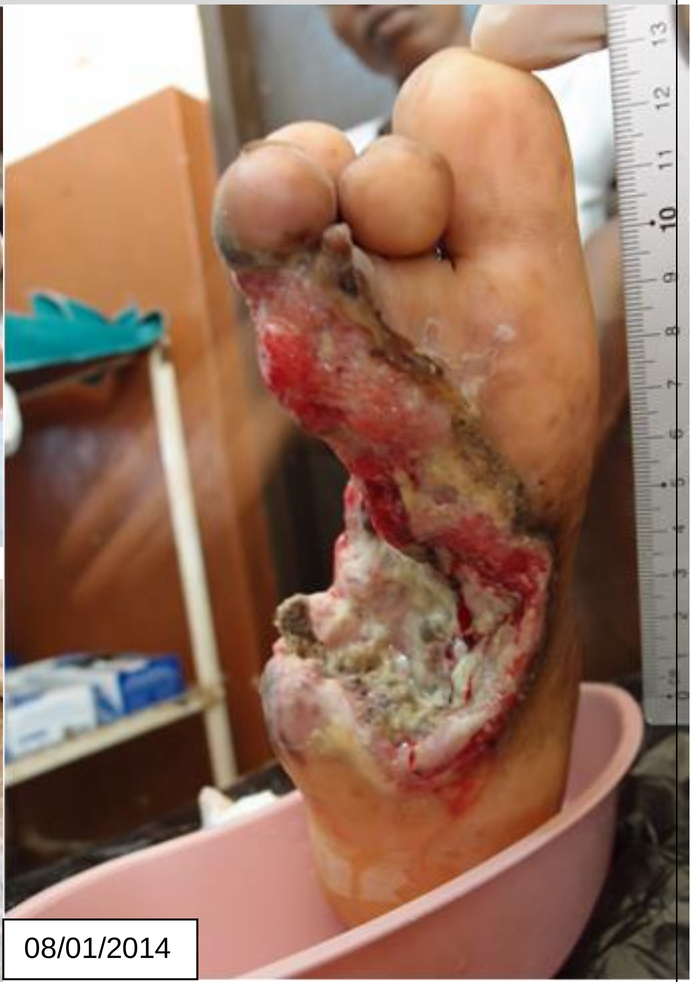

08/01/2014

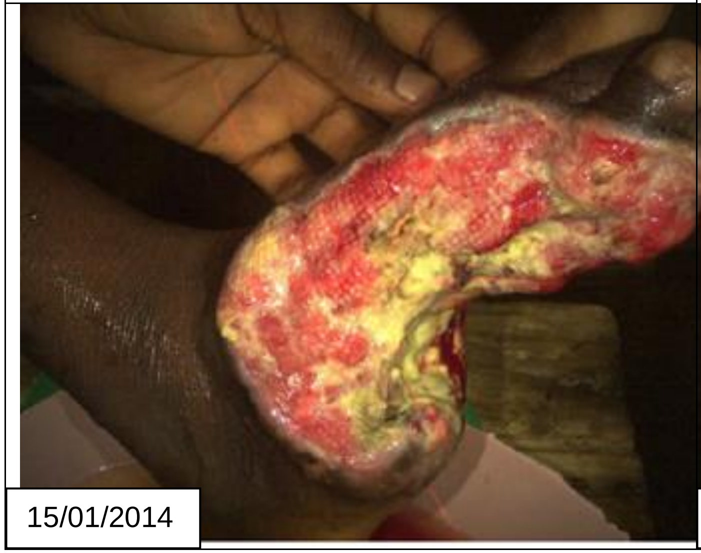

15/01/2014

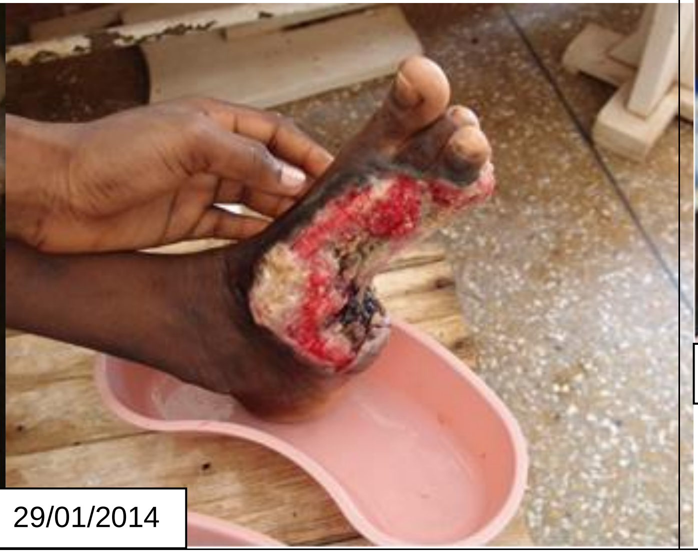

29/01/2014
