# Supplementary material for: Assessing and managing wounds of Buruli ulcer patients at the primary and secondary health care levels in Ghana
Source: PLoS Negl Trop Dis. 2017 Feb 28;11(2):e0005331. doi: 10.1371/journal.pntd.0005331 (PMC5345880; doi:10.1371/journal.pntd.0005331)
Supplement: S1 Ethical clearance — (PDF) [file pntd.0005331.s007.pdf]

GHANA HEALTH SERVICE ETHICAL REVIEW COMMITTEE

In case of reply the number and date of this Letter should be quoted.

My Ref. :GHS-ERC 070713-1
Your Ref. No.

Your Health - Our Concern

Research & Development Division
Ghana Health Service
P. O. Box MB 190
Accra
Tel: +233-302-681109
Fax + 233-302-685424
Email: nanatuesdaykad@yahoo.com

1st October, 2013

Professor Dorothy Yeboah-Manu
Noguchi Memorial Institute for Medical Research
University of Ghana
Accra

ETHICAL CLEARANCE - ID NO: GHS-ERC: 07/07/13

The Ghana Health Service Ethics Review Committee has reviewed and given approval for the implementation of your Study Protocol titled:

"Diagnosis and systematic follow up of buruli ulcer patients in a district hospital and in a health centre"

This approval requires that you submit an Inception and Mid-term reports of the study to the Ethical Review Committee (ERC) for continuous review. The ERC may observe or cause to be observed procedures and records of the study during and after implementation.

Please note that any modification of the project must be submitted to the ERC for review and approval before its implementation.

You are also required to report all serious adverse events related to this study to the ERC within seven days verbally and fourteen days in writing.

You are requested to submit a final report on the study to assure the ERC that the project was implemented as per approved protocol. You are also to inform the ERC and your mother organization before any publication of the research findings.

Please always quote the protocol identification number in all future correspondence in relation to this protocol

SIGNED..

PROFESSOR FRED BINKA
(GHS-ERC - CHAIRMAN)

Cc: The Director, Research & Development Division, Ghana Health Service, Accra
