# Supplementary material for: Assessing and managing wounds of Buruli ulcer patients at the primary and secondary health care levels in Ghana
Source: PLoS Negl Trop Dis. 2017 Feb 28;11(2):e0005331. doi: 10.1371/journal.pntd.0005331 (PMC5345880; doi:10.1371/journal.pntd.0005331)
Supplement: S1 Wound documentation Recapture Study — (PDF) [file pntd.0005331.s008.pdf]

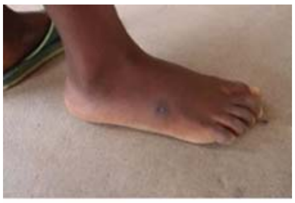
Lesion 1

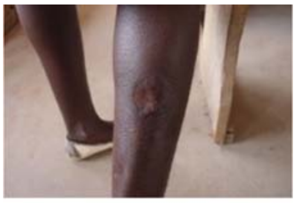
Lesion 2

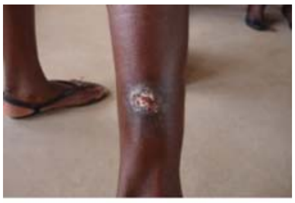
Lesion 3A

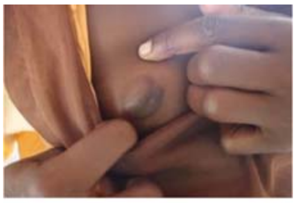
Lesion 3B

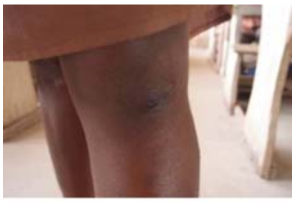
Lesion 4A

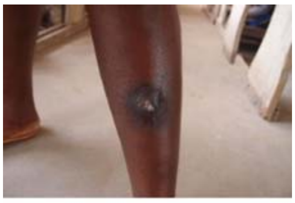
Lesion 4B

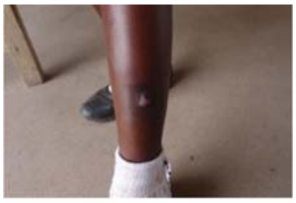
Lesion 5

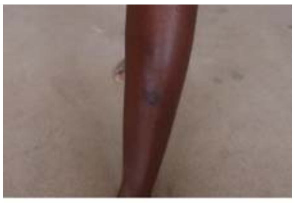
Lesion 6

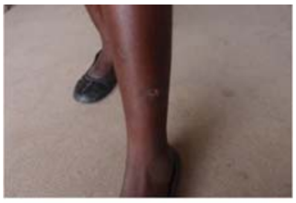
Lesion 7

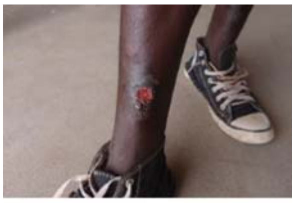
Lesion 8

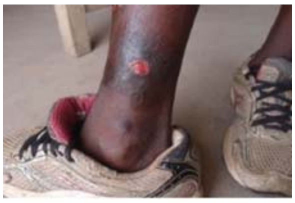
Lesion 9

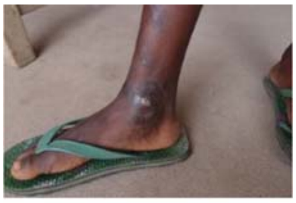
Lesion 10

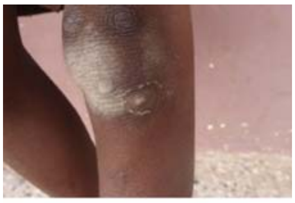
Lesion 11

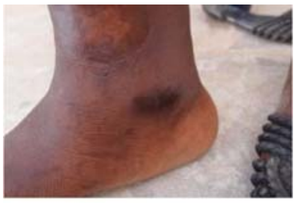
Lesion 12

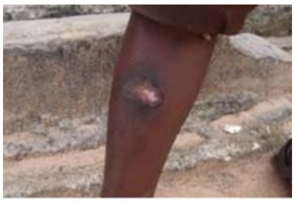
Lesion 13

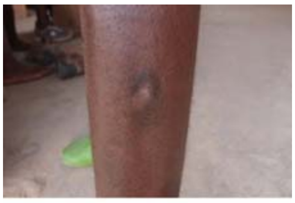
Lesion 14

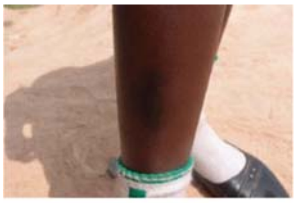
Lesion 15

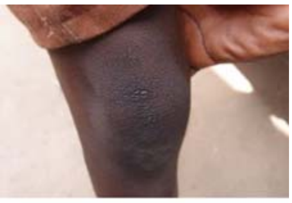
Lesion 16

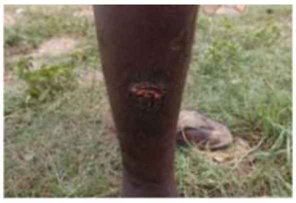
Lesion 17

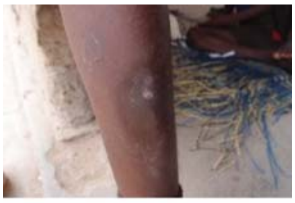
Lesion 18

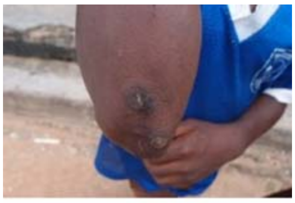
Lesion 19

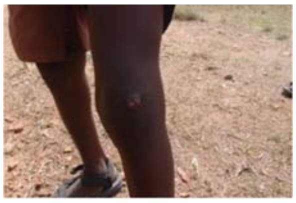
Lesion 20

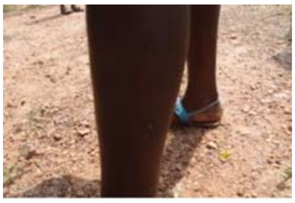
Lesion 21

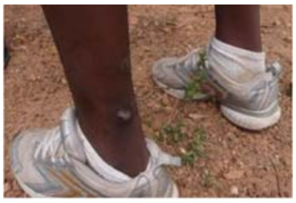
Lesion 22

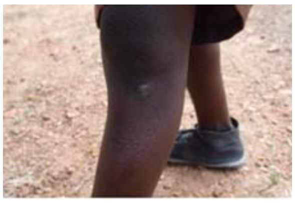
Lesion 23

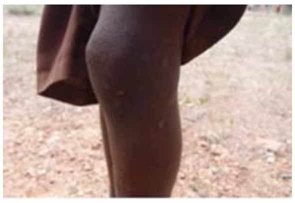
Lesion 24

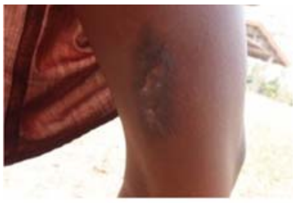
Lesion 25

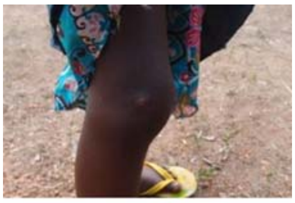
Lesion 26

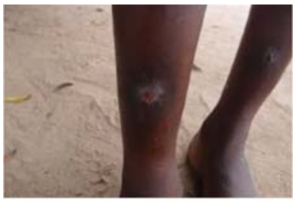
Lesion 27

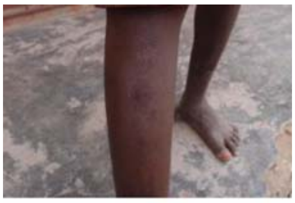
Lesion 28

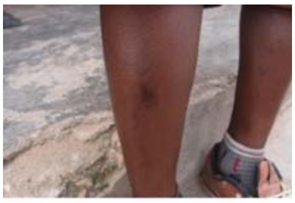
Lesion 29

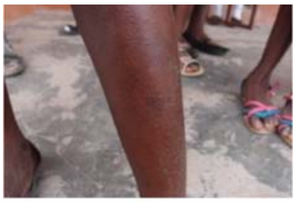
Lesion 30

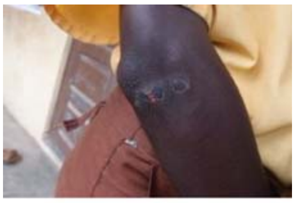
Lesion 31

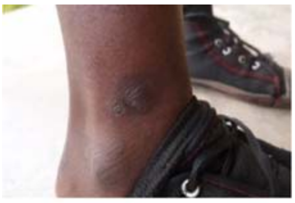
Lesion 32

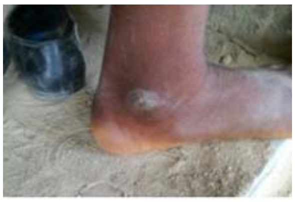
Lesion 33

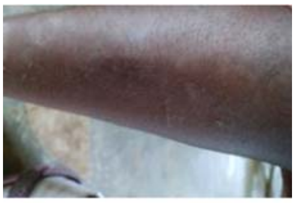
Lesion 34
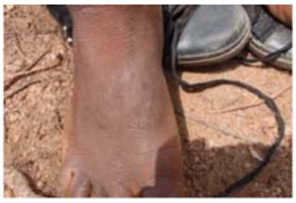
Lesion 35
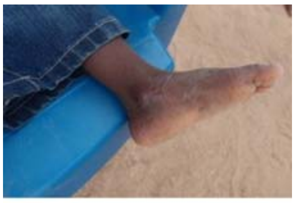
Lesion 36
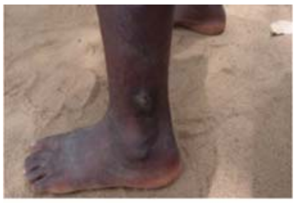
Lesion 37
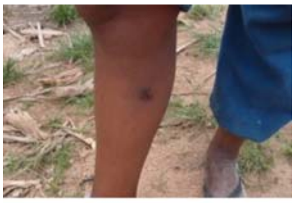
Lesion 38
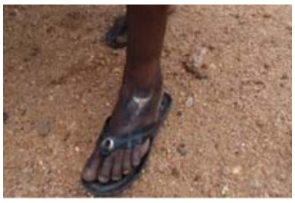
Lesion 39
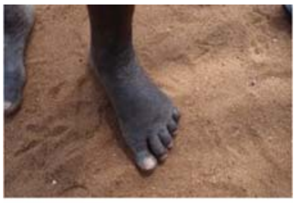
Lesion 40
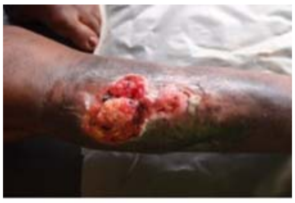
Lesion 41
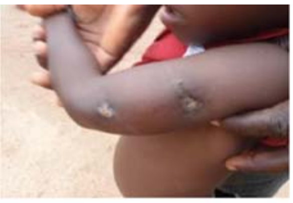
Lesion 42
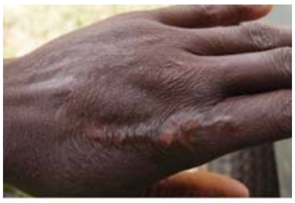
Lesion 43
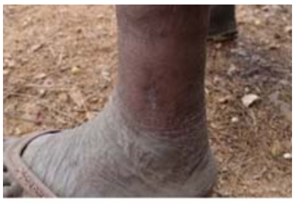
Lesion 44
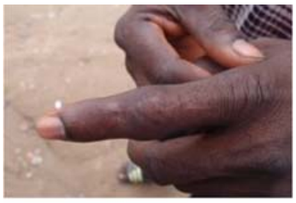
Lesion 45
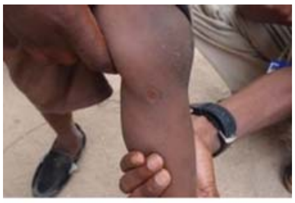
Lesion 46
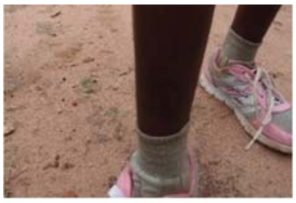
Lesion 47
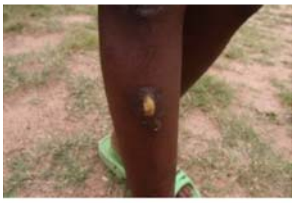
Lesion 48
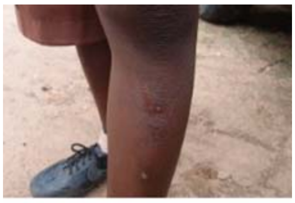
Lesion 49
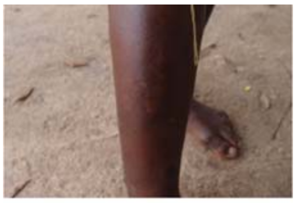
Lesion 50
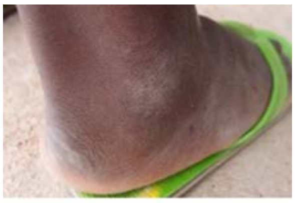
Lesion 51
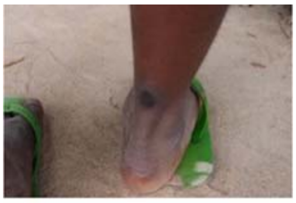
Lesion 52
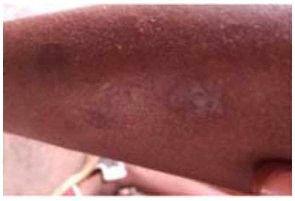
Lesion 53
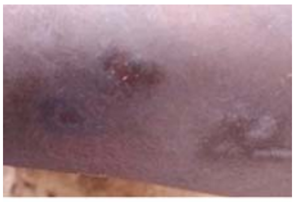
Lesion 54
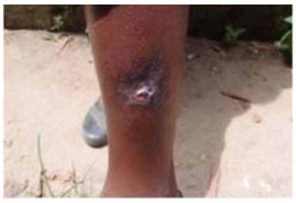
Lesion 55
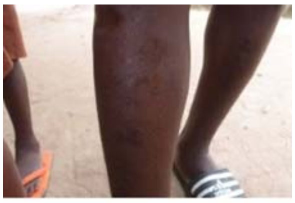
Lesion 56
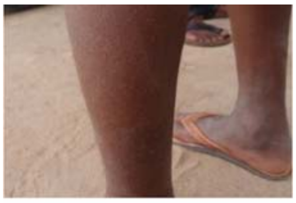
Lesion 57
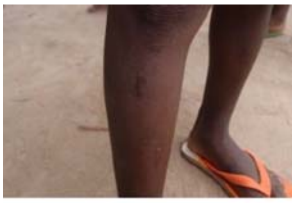
Lesion 58
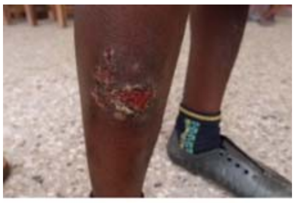
Lesion 59
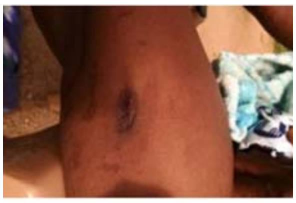
Lesion 60
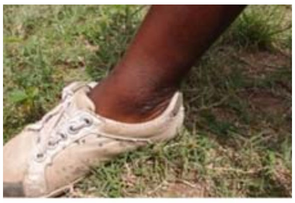
Lesion 61
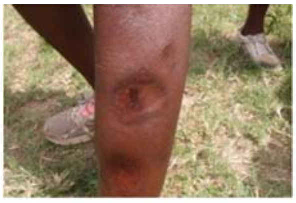
Lesion 62
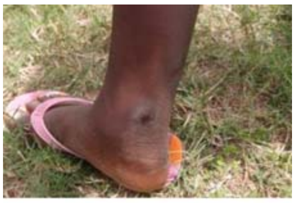
Lesion 63
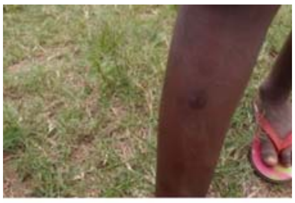
Lesion 64
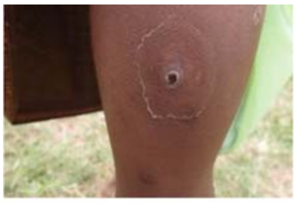
Lesion 65
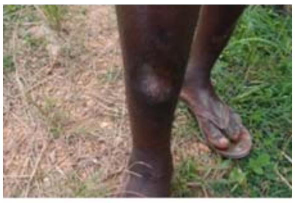
Lesion 66
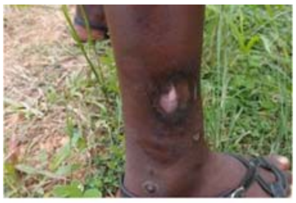
Lesion 67
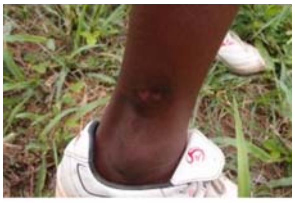
Lesion 68

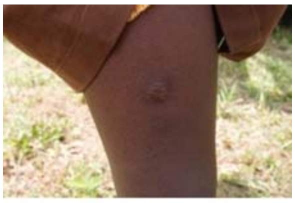
Lesion 69

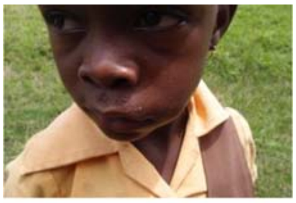
Lesion 70

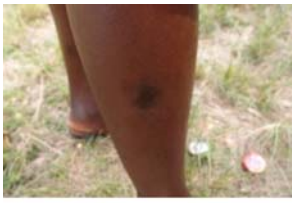
Lesion 71

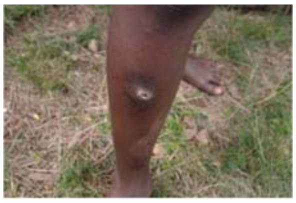
Lesion 72

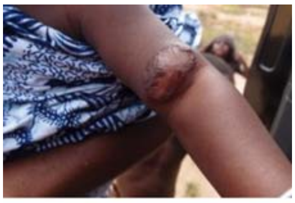
Lesion 73

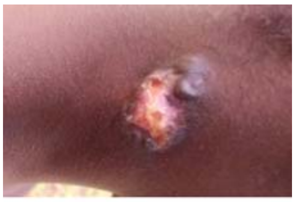
Lesion 74

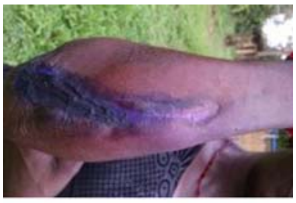
Lesion 75

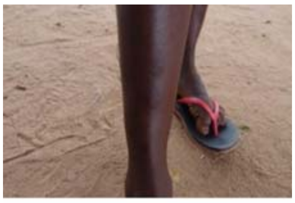
Lesion 76

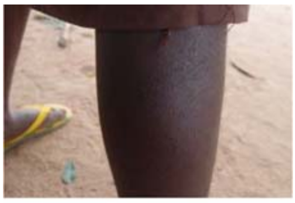
Lesion 77

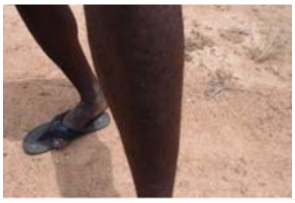
Lesion 78

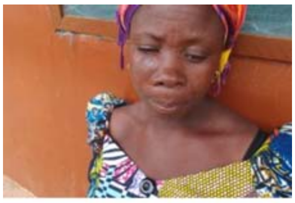
Lesion 79

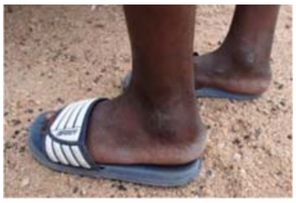
Lesion 80
